# *Bicharracosaurus dionidei,* gen. et sp. nov., a new macronarian (Dinosauria, Sauropoda) from the Late Jurassic Cañadón Calcáreo Formation of Argentina and the problematic early evolution of macronarians

**DOI:** 10.7717/peerj.20945

**Published:** 2026-04-16

**Authors:** Alexandra Reutter, José Luis Carballido, Guillermo José Windholz, Diego Pol, Oliver W.M. Rauhut

**Affiliations:** 1 Department of Earth and Environmental Sciences, Ludwig-Maximilians-Universität München, Munich, Germany; 2 Museo Paleontológico Egidio Feruglio, Trelew, Argentina; 3 Consejo Nacional de Investigaciones Científicas y Técnicas, Buenos Aires, Argentina; 4 Instituto de Investigación en Paleobiología y Geología, Universidad Nacional de Río Negro, General Roca, Río Negro, Argentina; 5 Museo Argentino de Ciencias Naturales Bernardino Rivadavia, Buenos Aires, Argentina; 6 Bayerische Staatssammlung für Paläontologie und Geologie, Staatliche Naturwissenschaftliche Sammlungen Bayerns, Munich, Germany; 7 GeoBioCenter, Ludwig-Maximilians-Universität München, Munich, Germany

**Keywords:** Sauropoda, Macronaria, Brachiosauridae, Late Jurassic, Gondwana, Phylogeny, Phylogenetic incongruence

## Abstract

Our understanding of Late Jurassic sauropod faunas heavily relies on the fossil record of the northern hemisphere. During the last two decades, paleontological fieldwork in the Oxfordian-Kimmeridgian Cañadón Calcáreo Formation of Argentina has yielded several sauropod remains. Here we present a new taxon, *Bicharracosaurus dionidei* gen. et sp. nov., represented by a partial vertebral column and a fragment of the ilium. Anatomical as well as histological evidence suggest that the new specimen represents an adult individual. The posterior dorsals of *Bicharracosaurus* show great similarity with isolated mid- to posterior dorsal vertebrae previously referred to a diplodocid (MPEF-PV 1324) from the same formation. Some characters that supported a diplodocid position of the isolated vertebrae (pleurocoels with anteroventral fossa, dorsal margin of the pleurocoel angular and at the level of the neural canal) are also present in *Bicharracosaurus*. The phylogenetic position of *Bicharracosaurus* was tested using two recent datasets that include a large sample of basal eusauropods and basal neosauropods. The overall evidence supports a position of *Bicharracosaurus* within Macronaria with several analyses and diagnostic characters suggesting brachiosaurid affinities. Given the incomplete nature of the isolated vertebrae MPEF-PV 1324, their position is unstable across several analyses, but they show close affinities with either *Bicharracosaurus* or Diplodocidae. Despite the similarities between *Bicharracosaurus* and *Tehuelchesaurus benitezii*, a macronarian from the same formation, only in some of the phylogenetic results these two species were recovered as closely related, whereas in most analyses, *Tehuelchesaurus* formed a clade with *Janenschia robusta* as basal macronarians or non-neosauropod eusauropods. In addition, several diagnostic characters of *Bicharracosaurus* are absent in *Tehuelchesaurus* and vice versa. Our results also show that other putative macronarian taxa have incongruent positions depending on the dataset, even when controlling for taxonomic scope hindering our understanding of the early evolution of the clade.

## Introduction

Sauropoda is one of the main dinosaur lineages that represents one of the most important groups of herbivorous vertebrates during the Mesozoic. Sauropods first appeared in the latest Triassic and survived until the end of the Cretaceous (Upchurch, Barrett & Dodson, 2004). The group was taxonomically diverse and included the largest terrestrial animals that ever lived (Bonaparte & Coria, 1993; Sander & Clauss, 2008; Sander et al., 2011; Carballido et al., 2017b). Within Sauropoda, a number of early-branching clades that flourished during the Jurassic were largely replaced by the derived Neosauropoda towards the end of this period (Upchurch, Barrett & Dodson, 2004). Neosauropoda split early in their evolution into two major clades, the Diplodocoidea and the Macronaria, but, although this principal subdivision of Neosauropoda has been generally accepted since it was first proposed in the late 1990s (Wilson & Sereno, 1998; Upchurch, 1998), there is still considerable debate about the referral of many taxa, especially from the Jurassic, to either of these subclades, or to Neosauropoda in general (Carballido et al., 2017b; Xu et al., 2018; González Riga et al., 2018; Mannion et al., 2019b; Moore et al., 2020; Upchurch et al., 2021; Ren et al., 2021; Moore et al., 2023; Ren et al., 2023).

**Figure 1:**
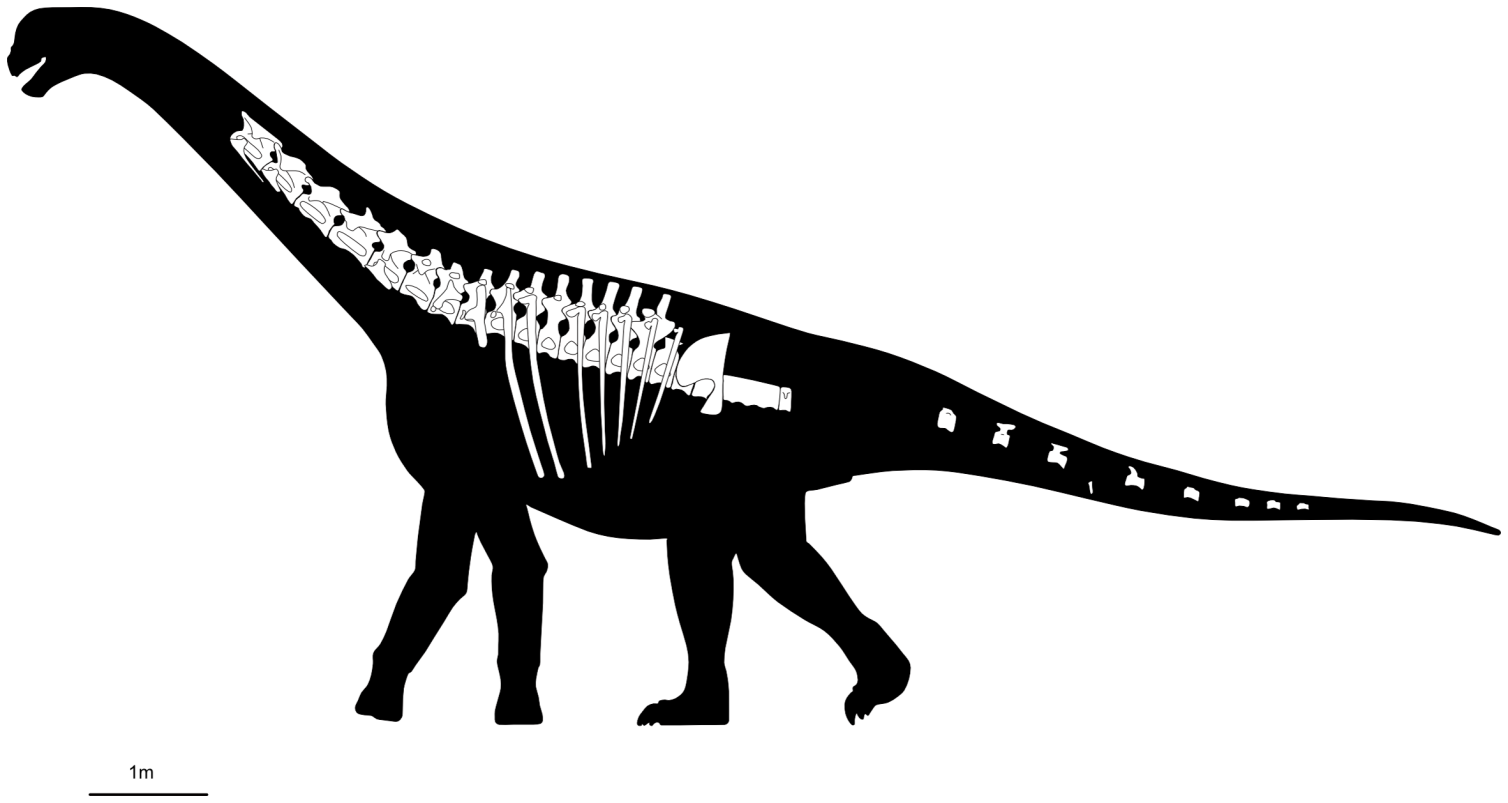
Reconstruction of *Bicharracosaurus dionidei* (MPEF-PV 1730). Skeletal reconstruction showing preserved elements. Some ribs are depicted here as left elements for visual purposes. Image source credit: Silhouette based on *Camarasaurus* by Scott Hartman: https://www.phylopic.org/images/bae62f3c-acac-410d-b5c6-b6d1d3e2ef59/camarasaurus-lentus, CC-BY-BC-sa/3.0.

The origin of Neosauropoda can be traced back at least to the Middle and probably to the end of the Early Jurassic (Alifanov & Averianov, 2003; Carballido et al., 2017a; Xu et al., 2018; Ren et al., 2023). Since terrestrial rocks from this period are scarce worldwide, our understanding of this evolutionary event and the unfolding of neosauropod diversity is mainly based on the Late Jurassic fossil record from the northern hemisphere (see Rauhut & López-Arbarello, 2008). The Tendaguru Formation of Tanzania and the Cañadón Calcáreo Formation of Argentina are the only Gondwanan formations from that time that have yielded several articulated or closely associated sauropod remains. Sauropods from the latter unit include the putative basal macronarian *Tehuelchesaurus benitezii* (Rich et al., 1999; Carballido et al., 2011b), the dicraeosaurid *Brachytrachelopan mesai* (Rauhut et al., 2005), fragmentary remains of a possible brachiosaurid (Rauhut, 2006), and isolated vertebral centra preliminarily reported as the first diplodocid from this unit and thus from the Late Jurassic of South America in general (Rauhut, Carballido & Pol, 2015). Here, we present a new macronarian from the Late Jurassic Cañadón Calcáreo Formation of Argentina that reveals a great similarity with these supposed diplodocid vertebrae, but presents several traits that are different from *Tehuelchesaurus*, allowing us to erect a new genus and species (Fig. 1). Furthermore, we use two recent phylogenetic matrices, those of Upchurch et al. (2021) and Ren et al. (2023), to test the position of the new taxon. We also highlight the incongruent position of several Jurassic taxa and discuss the possible causes and potential pitfalls of differing results.

### Geological setting

**Figure 2:**
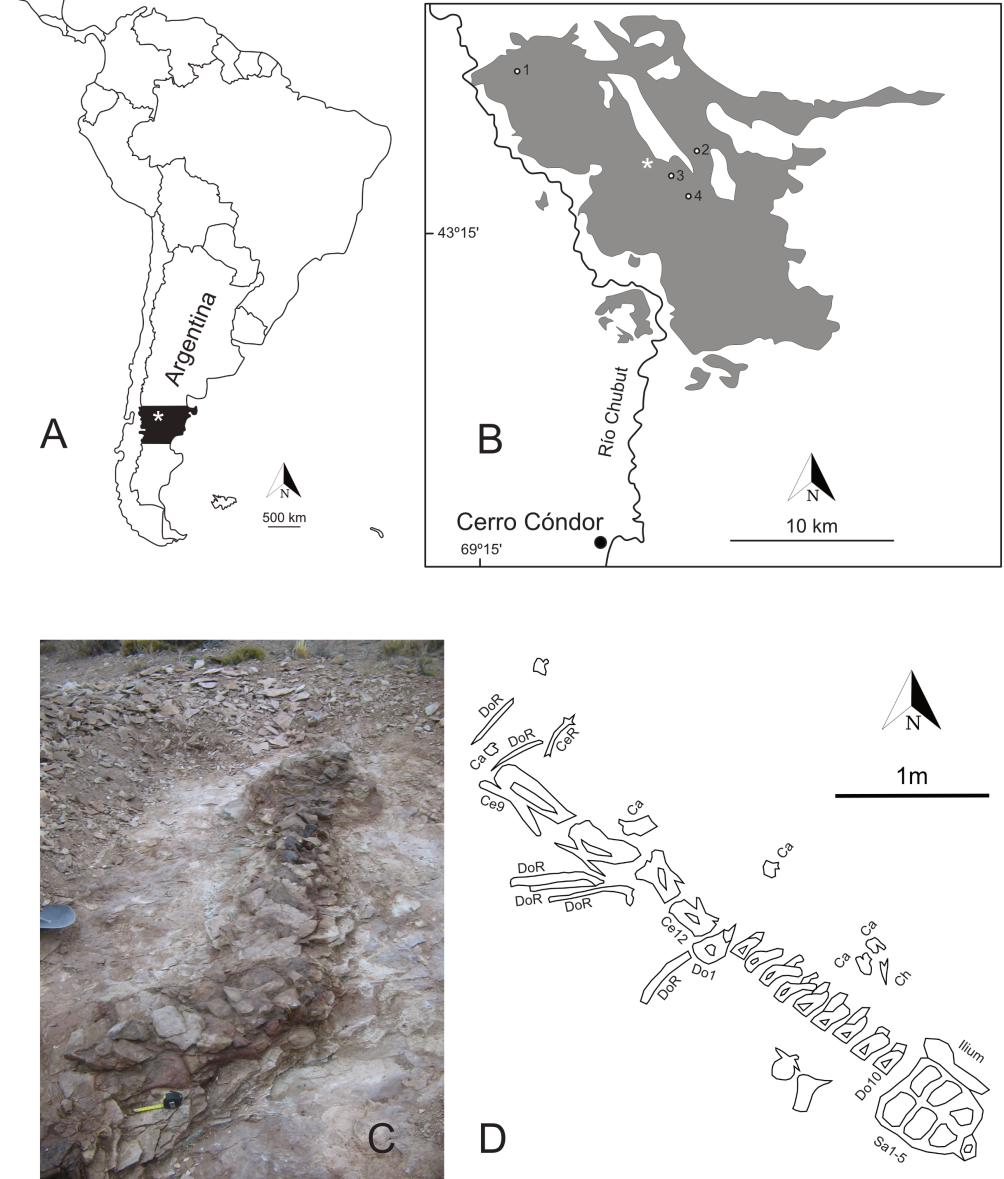
Provenance of *Bicharracosaurus dionidei*. (A) Geographical map showing the position of *Bicharracosaurus* (white asterisk). (B) Geological map (based on Rauhut, Carballido & Pol, 2015) showing the position of *Bicharracosaurus* (white asterisk) from the Cañadón Calcáreo Formation (gray shading), numbers indicate the position of: 1, *Tehuelchesaurus benitezii*; 2, *Brachytrachelopan mesai*; 3, putative brachiosaurid remains (Rauhut, 2006), and 4, putative diplodocid (Rauhut, Carballido & Pol, 2015). (C) Photograph of the quarry (Photo credit: Pablo Puerta) with the sacrum at the top of the image. (D) Quarry map of *Bicharracosaurus* (three additional cervical vertebrae were found closely associated, north-west of the main specimen, a few years later and are not shown in this map). Abbreviations: Ca, caudal vertebra; Ce, cervical vertebra; CeR, cervical rib; Ch, chevron; Do, dorsal vertebra; DoR, dorsal rib; Sa, sacral vertebra.

The Cañadón Calcáreo Formation, exposed in the central to northern parts of Chubut Province of Argentina (Fig. 2A), represents the post-rift infill phase of the Cañadón Asfalto Basin, a large, roughly North-North West to South-South East trending hemigraben structure in central Patagonia (Figari, 2005; Figari et al., 2015). The formation represents a thick succession of mainly fluvial and lacustrine terrestrial sediments. The base of the unit was dated (157.4 Mya) to the Oxfordian-Kimmeridgian boundary (Cohen et al., 2025), using U-Pb chronostratigraphy (Cúneo et al., 2013; Rauhut, Carballido & Pol, 2015).

The specimen described herein comes from the central part of the main outcrop area, east of the Chubut River (Fig. 2B). The layers containing the fossil were semihorizontal. The matrix surrounding the specimen consists of tabular sandstone sheets interbedded with mudstones, consistent with low energy floodplain deposits from high in the depositional sequence of the formation. The main part of the axial series, composed of four cervical vertebrae (MPEF-PV 1730/4-7), ten dorsal vertebrae (MPEF-PV 1730/7-12), and five sacral vertebrae articulated with the fragmentary right ilium (MPEF-PV 1730/13a-c), was found articulated or closely associated with the sacrum exposed at the surface (Figs. 2C, 2D). Three additional cervical vertebrae (MPEF-PV 1730/1-3) were later found in proximity, some two to three meters north-west of the articulated column. Four cervical ribs (MPEF-PV 1730/23-26), ten dorsal ribs (MPEF-PV 1730/29-38), nine caudal vertebrae (MPEF-PV 1730/14-22), one fragmentary chevron (MPEF-PV 1730/28), and several undiagnostic sacral and rib elements were all found disarticulated in proximity of the main body.

It was not possible to confidently identify scavenger marks on the bone surfaces. However, a cast of a theropod tooth was recovered during excavation. It seems likely that the bones represent a single individual that was buried at or close to the place of death. The missing light elements of the body, such as the skull, could have been detached from the main body prior to burial and washed away with water currents, while the heavy elements might have been dispersed by scavengers.

## Materials and Methods

### Nomenclatural acts

The electronic version of this article in Portable Document Format (PDF) will represent a published work according to the International Commission on Zoological Nomenclature (ICZN), and hence the new names contained in the electronic version are effectively published under that Code from the electronic edition alone. This published work and the nomenclatural acts it contains have been registered in ZooBank, the online registration system for the ICZN. The ZooBank LSIDs (Life Science Identifiers) can be resolved and the associated information viewed through any standard web browser by appending the LSID to the prefix http://zoobank.org/. The LSID for this publication is: urn:lsid:http://zoobank.org:pub:A6452246-4BB8-471A-A943-6EA2DD964360. The online version of this work is archived and available from the following digital repositories: PeerJ, PubMed Central SCIE and CLOCKSS.

### Materials

The new specimen (MPEF-PV 1730) was brought to the attention of one of us (OWMR) by the local farmer Dionide Mesa in March 2001, and excavation of the site started in 2002. Most of the specimen was removed from the field in 2011, but additional cervical vertebrae were excavated in 2018. All the material was prepared in the preparation labs of the MPEF, where it is also stored permanently. *Tehuelchesaurus benitezii* (MPEF-PV 1125) and the putative diplodocid vertebrae (MPEF-PV 1324) were also studied in person by all the authors of this manuscript. Observations on other specimens are mentioned accordingly in the text.

### 3D surface scanning and modelling

The new specimen was scanned at the MPEF using the structured light 3D scanners Artec Eva (for the bigger elements) and Artec Space Spider (for elements smaller than 15 cm). The raw data was edited in Artec Studio 17, where the floating geometry was deleted manually, the shots were manually aligned, and the noise reduced. Subsequently, a 3D mesh was created using Sharp Fusion and the texture was projected onto the mesh. The 3D models are available in MorphoSource at: https://www.morphosource.org/concern/media/000774949?locale=en. The reconstruction of the articulated sacrum and ilium was done in Blender 4.2.

### Histology

Recent studies based on bone histology of dorsal rib bones of sauropod dinosaurs have demonstrated their usefulness in inferring the life history and ontogenetic stage of the specimens (*e.g.*, Waskow & Sander, 2014; Waskow & Mateus, 2017; Windholz et al., 2023). To infer the ontogenetic stages of the here studied specimen, histological samples were obtained from two dorsal ribs (MPEF-PV 1730/29 and /35). Both elements were sectioned in their proximal areas at the Paleohistological Laboratory of the MPEF.

### Identification of the first dorsal vertebra

The common definition of dorsal vertebrae is the one by Stannius (1846) (see Tschopp & Mateus, 2017), by which dorsal vertebrae have a common function, enclosing the thoracic cage in connection with the sternum *via* the ribs. However, in vertebrate paleontology, identifying the first dorsal by the first rib that is attached to the sternum (as *e.g.*, Janensch, 1929; Wilson & Upchurch, 2009), requires the preservation of the cervicodorsal vertebrae in articulation with the ribs, or the assignation of ribs to vertebrae. It also requires the ribs to be completely preserved and the assumption that ribs that attach to sternum have a distal expansion. This method is not really insightful in practice, when ribs are not preserved and/or collected (*e.g.*, Wilson & Upchurch, 2009), or their exact association with the vertebrae is uncertain. The use of free or fused ribs, for identifying the cervicodorsal boundary (Hatcher, 1901; Gilmore, 1936) is problematic, as we know that vertebral elements fuse during ontogeny and we do not fully understand patterns of co-ossification (Griffin et al., 2021). The use of a holistic approach based on morphology, taking into account the known changes that occur during the cervicodorsal transition (Tschopp & Mateus, 2017; Upchurch et al., 2021; Taylor, 2022; Moore et al., 2023; Van der Linden et al., 2024), remains subjective, as researchers will inevitably weigh some characteristics more than others. Using only the dorsal displacement of the parapophysis (*e.g.*, Schwarz et al., 2007; Moore et al., 2020) is a more pragmatic and precise approach, as it does not depend on the preservation of the ribs and one can set a clearer boundary. According to this criterion, the first dorsal vertebra is the first vertebra in which the parapophysis is no longer situated at the anteroventral end of the centrum but has moved to the mid-height of the anterior side of the centrum.

### Phylogenetic analyses

The relationships of the new taxon amongst sauropods were studied using the datasets published by Ren et al. (2023), from here on the RE23 matrix, and by Upchurch et al. (2021), from here on the UP21 matrix (Data S1–S2). The RE23 matrix, composed of 400 characters scored for 85 taxa, is a modified version of the Carballido et al. (2011a) dataset that was subsequently modified by Carballido & Sander (2014), Xu et al. (2018) and Ren, Huang & You (2020). Upchurch et al. (2021) used two matrices, one based on Mannion et al. (2019b) and the other based on Moore et al. (2020). Here, we only use the Mannion et al. (2019b) version, with 551 characters scored for 127 taxa. This version is based on the original dataset published by Mannion et al. (2013), which was recently revised and used in several analyses (Upchurch, Mannion & Taylor, 2015; Poropat et al., 2016; Mannion, Allain & Moine, 2017; González Riga et al., 2018; Mannion et al., 2019a). Both selected datasets include a vast array of eusauropods, including several Jurassic taxa, and so are well suited to test the general position of the new taxon within the sauropod tree. Moreover, these data matrices share several Jurassic taxa and show that for some, the general referral to either Macronaria or Neosauropoda is not consistent. More recent versions of the Mannion et al. (2019b) dataset (Poropat et al., 2021; Poropat et al., 2023; Díez Díaz et al., 2025) are focused on Late Cretaceous Somphospondyli and even titanosaurian interrelationships and so are outside the scope of this work.

As in previous iterations of the UP21 matrix, some fragmentary and unstable taxa were pruned *a priori* (*Astrophocaudia slaughteri, Brontomerus mcintoshi, Fukuititan nipponensis, Fusuisaurus zhaoi, Liubangosaurus hei, Malarguesaurus florenciae, Mongolosaurus haplodon, Histriasaurus boscarollii, Rayososaurus agrioensis* and *Vahiny depereti*). In this case, *Australodocus bohetii* was included in the analyses performed here, as it has been recovered within Brachiosauridae (Whitlock, 2011; Mannion et al., 2019b). In both matrices, the ordering of characters was treated as in previous versions. Additionally, we revised 13, inactivated three and added six characters to the RE23 matrix, and revised 14 and added five in the UP21 matrix. In addition to including the new specimen, we also revised the scorings of *Tehuelchesaurus, Janenschia robusta*, *Tendaguria tanzaniensis* and *Wamweracaudia keranjei* in both the RE23 and UP21 datasets. We also revised the scorings of MPEF-PV 1324 in the RE23 matrix and added this taxon to the UP21 dataset, and we revised the scorings for *Australodocus* in the UP21 matrix and added this taxon to the RE23 matrix. The emended version of the RE23 matrix includes 403 active characters scored for 87 taxa, while the UP21 matrix includes 556 characters scored for 119 active taxa. All changes are documented in Table S1.

Recent research on simulated morphological datasets has shown that parsimony analyses outperform or at least perform as well as model-based approaches (Goloboff, Torres & Arias, 2018; Goloboff et al., 2019). Within parsimony analyses, studies with simulated as well as empirical data have shown the superiority of implied weights (IW) (Goloboff, 1993) and extended implied weights (EIW) (Goloboff, 2014), over equal weights (EW) (see Goloboff, Farris & Nixon, 2008; Goloboff, Torres & Arias, 2018; Ezcurra, 2024). Both former methods, IW and EIW, weigh characters against homoplasy. EIW, which also considers the effect of missing entries on homoplasy, has outperformed IW in simulated morphological data. We performed tree searches under EW and EIW, using parsimony analyses in TNT 1.6 (Goloboff & Morales, 2023). Following Ezcurra (2024) and considering the number of terminals, the optimal range of k in the RE23 matrix is between three and 11, and for the UP21 matrix, between six and 13. We have chosen three values for k within these ranges, three as the strictest one (EIW3), eight as a middle value (EIW8) and 13 as the most relaxed value (EIW13). The New Technology search was used until the minimum length was found at least 30 times, using Sectorial Searches, Ratchet, Drifting and Tree Fusing algorithms. The random seed was altered when searches got stuck on a hit below 30. The most parsimonious trees (MPTs) were then subjected to a round of branch swapping using the tree bisection-reconnection (TBR) algorithm, holding up to 100.000 trees. Whenever the strict consensus tree was poorly resolved, the unstable taxa were identified using *IterPCR* (Pol & Escapa, 2009) as implemented in TNT (see Goloboff & Szumik, 2015).

We performed constraint analyses, forcing alternatives positions of the new taxon, only on the EW analyses. To estimate the number of extra steps more accurately, we selected some taxa as floaters. For the selection of floaters, we considered those taxa that were found together with *Bicharracosaurus* and taxa that could potentially be recovered within the clade being constrained, based on the results of the phylogenetic analyses of each matrix respectively. In order to test the significance of the forced topologies, we performed a Templeton Test (Templeton, 1983) as implemented by Carballido et al. (2020).

### Shared taxonomic scope analyses

Since taxon choice has been discussed as a major influence on phylogenetic results (Graybeal, 1998; Heath, Hedtke & Hillis, 2008), we performed parsimony analyses with the method described above on both matrices but taking into account only the shared taxonomic scope (STS) as proposed in Sereno (2009) (Data S3–S4). In other words, we only include taxa that are present in both datasets. The shared taxonomic scope includes only 64 terminals, representing 74% of the terminals in the RE23 dataset and 52% in the UP21 dataset. For the STS analyses, we removed taxa across the entire tree in both matrices, most of which were basal sauropodomorphs in the RE23 matrix and derived macronarians in the UP21 matrix. After the reduction in terminal taxa, all invariant characters were inactivated prior to analysis. The total number of active characters is 364 and 548 scored for 65 taxa in the RE23 and UP21 matrixes respectively. For these analyses, we focus only on EIW method with a value of seven for k (EIW7). Since these datasets are incomplete our focus is not on the position of specific taxa but on the differences between the topologies of both datasets.

### Terminology

We follow the histological nomenclature proposed by Francillon-Vieillot et al. (1990) and de Buffrénil & Quilhac (2021).

Nomenclature and abbreviations for vertebral laminae follow Wilson (1999), Wilson (2012), Apesteguía (2005), Taylor (2009), Salgado & Powell (2010), Carballido et al. (2012), Tschopp & Mateus (2013) and Carballido & Sander (2014) (see Table S2) and for vertebral fossae follow Wilson et al. (2011).

The use of ‘pleurocoel’ herein follows the definition of Carballido & Sander (2014: p. 337): a lateral excavation with well-defined anterior, ventral and dorsal margins. By this definition a pleurocoel is independent from the internal pneumatic structures.

The terms ‘acamerate’, ‘camerate’, and ‘camellate’ refer to the internal pneumatic morphology of the centra as originally proposed by Wedel, Cifelli & Sanders (2000: table 3). In that contribution, the authors defined four extra pneumatic categories, that are not normally used in phylogenetic analyses. Carballido et al. (2015: C139) introduced one of these extra categories ‘polycamerate’, in their character matrix. The authors identified this morphology in the dorsal vertebra of *Padillasaurus leivaensis* and compared it to the one in *Tastavinsaurus sanzi* and *Giraffatitan brancai*. By definition (Wedel, Cifelli & Sanders, 2000), the pneumatic morphology in the presacral vertebrae of *Giraffatitan* (Janensch, 1950: figs. 68-73) should be identified as camerate and not polycamerate, given that there are less than three camellate generations. However, since not all seven categories are normally used, a slight shift in meaning is proposed here to distinguish between camerae without a branching pattern (camerate) and camerae with a branching pattern regardless of the number of cameral generations (polycamerate).

The terms ‘basal’ and ‘early-branching’ are used interchangeably in reference to the following paraphyletic groups: non-Eusauropoda Sauropodomorpha, non-Neosauropoda Eusauropoda, non-Diplodocimorpha and non-Titanosauriformes Neosauropoda; non-Diplodocimorpha Diplodocoidea and non-Titanosauriformes Macronaria.

## Results

### Systematic Paleontology

**Table:** 

DINOSAURIA OWEN, 1842
SAURISCHIA SEELEY, 1887
SAUROPODOMORPHA HUENE, 1932
SAUROPODA MARSH, 1878
EUSAUROPODA UPCHURCH, 1995
NEOSAUROPODA BONAPARTE, 1986B
MACRONARIA WILSON & SERENO, 1998
*Bicharracosaurus* gen. nov.
Type species: *Bicharracosaurus dionidei* sp. nov.

Etymology: From *bicharraco* meaning big animal in informal Spanish and used by Dionide Mesa to refer to the big size of the fossils, and *saurus* (sauros), Greek word for lizard. The epithet *dionidei*, honours Dionide Mesa, who found this specimen and other dinosaur bones on his farm.

Holotype: MPEF-PV 1730 (Fig. 1), partially articulated axial skeleton composed of seven middle to posterior cervical vertebrae (MPEF-PV 1730/1-7), four disarticulated and fragmentary cervical ribs (MPEF-PV 1730/23-26), complete dorsal column composed of ten vertebrae (MPEF-PV 1730/7-12), ten disarticulated dorsal ribs (MPEF-PV 1730/29-38), histological thin-sections of two dorsal ribs (MPEF-PV 1730/29 and /35), the sacrum composed of five articulated vertebral centra with parts of the sacricostal yoke and fused to the preacetabular process of the right ilium (MPEF-PV 1730/13), nine disarticulated caudal vertebrae (MPEF-PV 1730/14-22), one fragmentary chevron (MPEF-PV 1730/28) and several undiagnostic sacral and rib elements.

Locality and horizon: Dionide 3 locality, approximately 24 km north of Cerro Cóndor and east of the Chubut River, Chubut Province, Argentina. Higher parts of the Cañadón Calcáreo Formation, Oxfordian-Kimmeridgian, Late Jurassic (Cúneo et al., 2013).

Referred specimens: MPEF-PV 1324, posterior dorsal vertebral centra (Rauhut, Carballido & Pol, 2015).

Diagnosis: The new genus and species can be diagnosed by three unique autapomorphies (denoted with an asterisk) and the following unique combination of characters: ventral surface of middle cervical centra with an anteroposterior short ridge in the posterior portion laterally bound by paired rounded fossae; ventrally bifurcated postzygodiapophyseal lamina in posterior cervical vertebrae; CDF in middle and posterior cervical vertebrae pierced by a triangular coel*; presence of a vertically oriented accessory lamina within the PRCDF in the posteriormost cervical vertebra; presence of several fossae along the ventral margin (or the PCDL) of the POCDF in middle cervical vertebrae; distal portion of the PODL with a small dorsally oriented protuberance in posteriormost cervical vertebrae*; presence of a prezygapophyseal fossa in middle cervicals; absence of pre-epipophyses in cervical vertebrae; SDF in middle cervical vertebrae pierced by several rounded coels; slightly opisthocoelous posterior dorsal centra; small fossa anteroventral to pleurocoel in middle to posterior dorsal vertebrae; parapophysis dorsal to prezygapophysis in middle and posterior dorsal vertebrae; accessory lamina of the parapophysis in middle to posterior dorsal vertebrae; divided SPDL in anterior dorsal vertebra; accessory lamina of the hyposphene; absence of aliform process in middle and posterior dorsal neural spines; posterior dorsal neural spines transversely narrower than anteroposteriorly long; boat-like articular facets of sacral centra*; second sacral rib with dual contribution from Sa1 and Sa2; ilium with a semi-horizontal preacetabular process.

### Paleohistological characterization and ontogenetic stage

**Figure 3:**
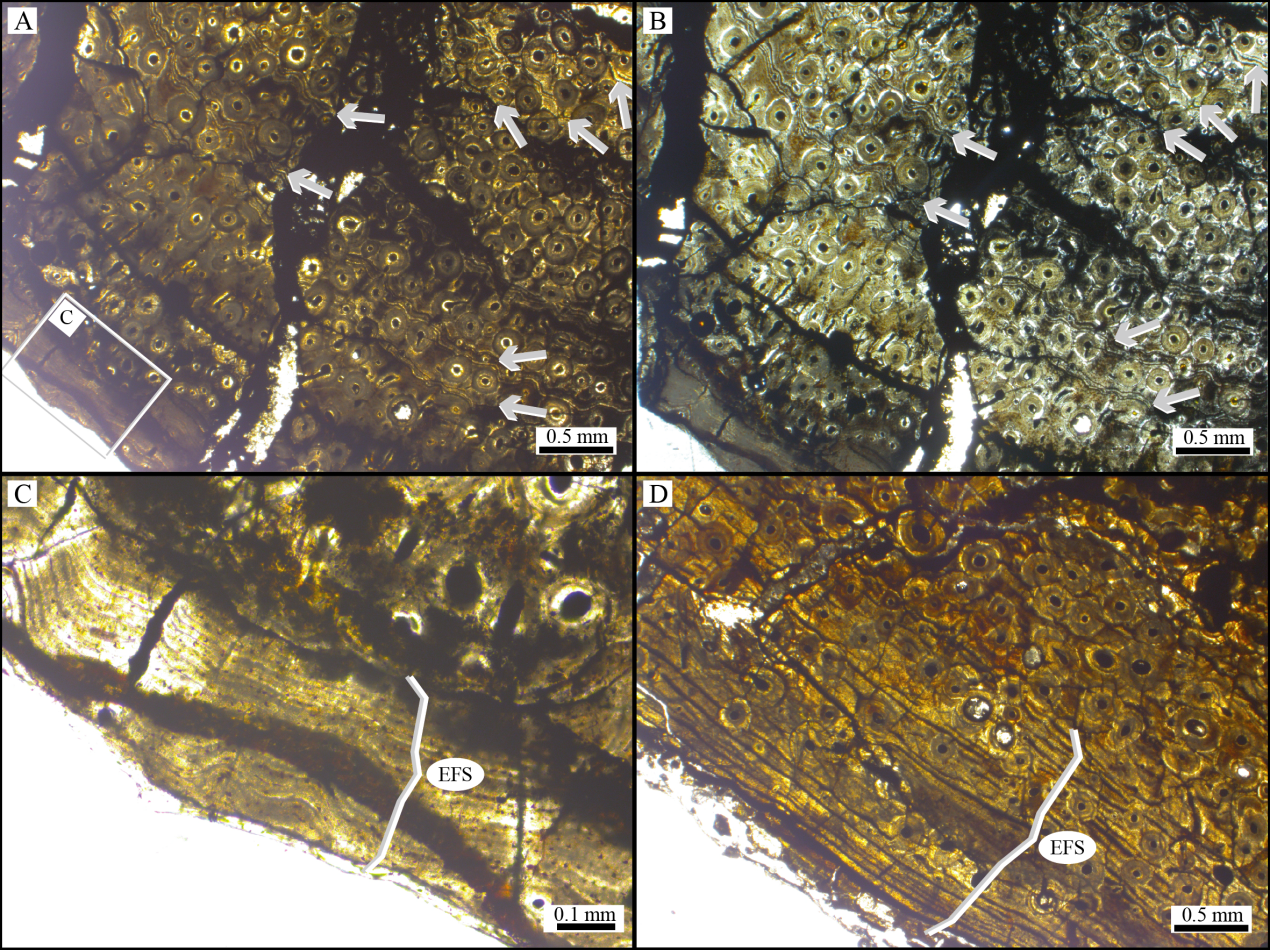
Histological samples of the dorsal ribs of *Bicharracosaurus dionidei*. (A) normal light microscopy and (B) polarized light showing a fibrolamellar bone matrix with at least seven cyclical growth marks (arrowheads) in MPEF-PV 1730/35. (C) MPEF-PV 1730/35 and (D) MPEF-PV 1730/29 detail of external fundamental system (EFS).

Due to histological similarity the thin sections are described together, highlighting differences when necessary. The medullary region of the specimens (in both ribs) is composed of trabecular bone, while the cortical area is composed of compact bone. The compacta exhibit extensive remodelling, with secondary osteons reaching into the most cortical region of the samples (Fig. 3). The primary tissue is fibrolamellar, with mainly longitudinal vascular canals. Osteocyte lacunae exhibit rounded shapes and they are randomly distributed. The sample MPEF-PV 1730/29 has a high degree of remodelling, making it difficult to test for the presence of cyclical growth marks (CGMs). However, MPEF-PV 1730/35 exhibits at least seven CGMs expressed as annuli (Figs. 3A–3B), as observed in most sauropod dinosaurs (*e.g.*, Chinsamy-Turan, 2005; Klein & Sander, 2008; Cerda et al., 2017). By assuming an annual deposition, the CGMs suggest a minimum longevity of seven years for the individual before perishing. Both histological samples of dorsal ribs show, in their subperiosteal area, a poorly vascularized layer with abundant closely spaced CGMs (Figs. 3C–3D) that is interpreted as external fundamental system (EFS). This histological structure, also known as outer circumferential layer, suggests that the specimen here studied would have reached somatic maturation, a typical feature of vertebrates with determinate growth (Chinsamy-Turan, 2005).

In all vertebrae the neurocentral suture is completely closed and not visible anymore. None of the cervical ribs were found in articulation, but articular facets of middle cervicals suggest that these were fused and broken during preservation. Also, sacral vertebrae are fused to one another and to the ribs forming a sacricostal yoke. Even though patterns of fusion or co-ossification events remain ambiguous (Wedel & Taylor, 2013; Griffin et al., 2021), they suggest that the type specimen of *Bicharracosaurus* reached skeletal maturity, in concordance with the histological analysis.

### Description and comparisons

#### Cervical vertebrae

**Figure 4:**
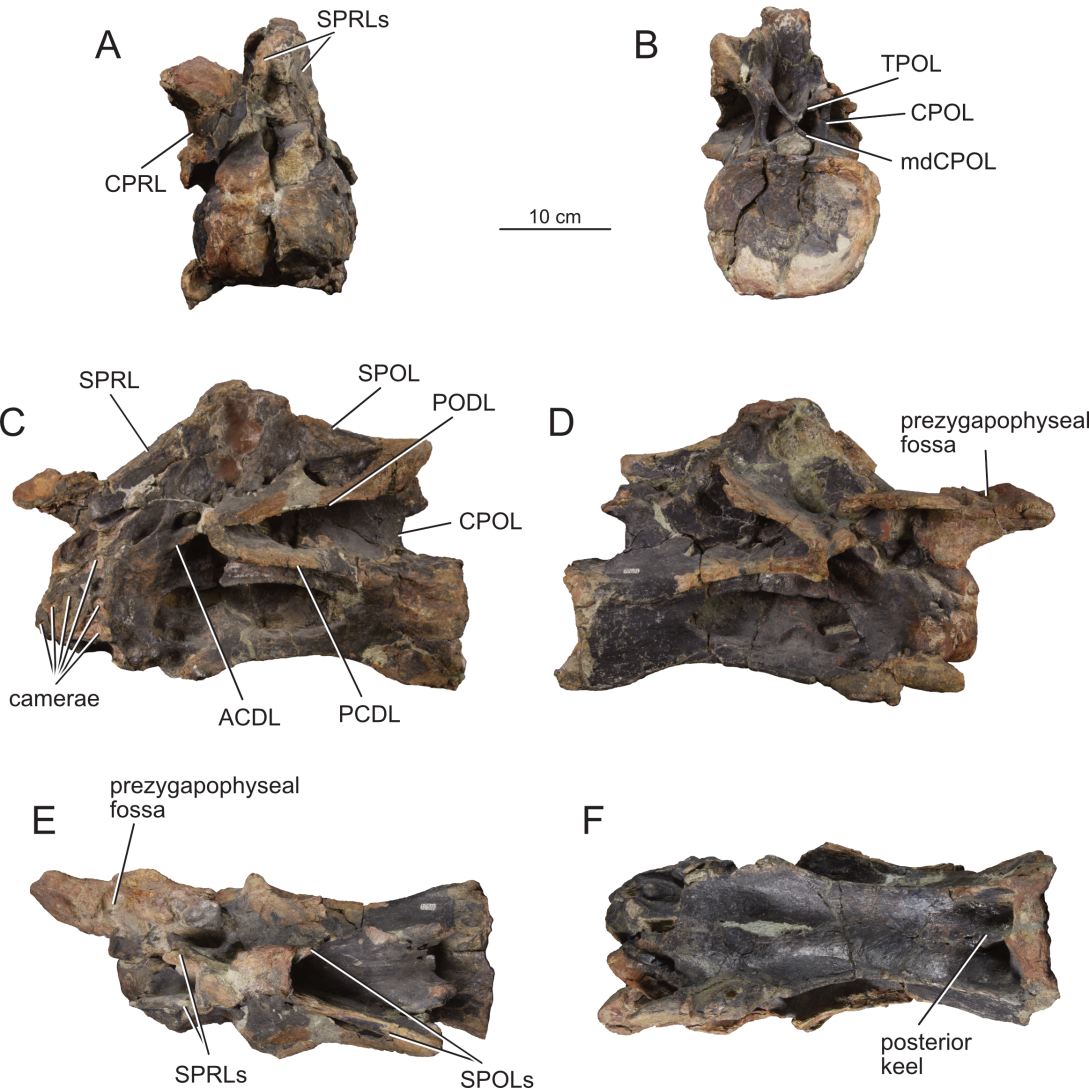
Middle cervical vertebra Ce6 (MPEF-PV 1730/1) of *Bicharracosaurus dionidei*. In (A) anterior; (B) posterior; (C) left lateral; (D); right lateral; (E) dorsal and (F) ventral views. Abbreviations: ACDL, anterior centrodiapophyseal lamina; CPOL, centropostzygapophyseal lamina; CPRL, centroprezygapophyseal lamina; mdCPOL, medial division of the centropostzygapophyseal; PCDL, posterior centrodiapophyseal lamina; PODL, postzygodiapophyseal lamina; SPOL, spinopostzygapophyseal lamina; SPRL, spinoprezygapophyseal lamina; TPOL, interpostzygapophyseal lamina.

**Figure 5:**
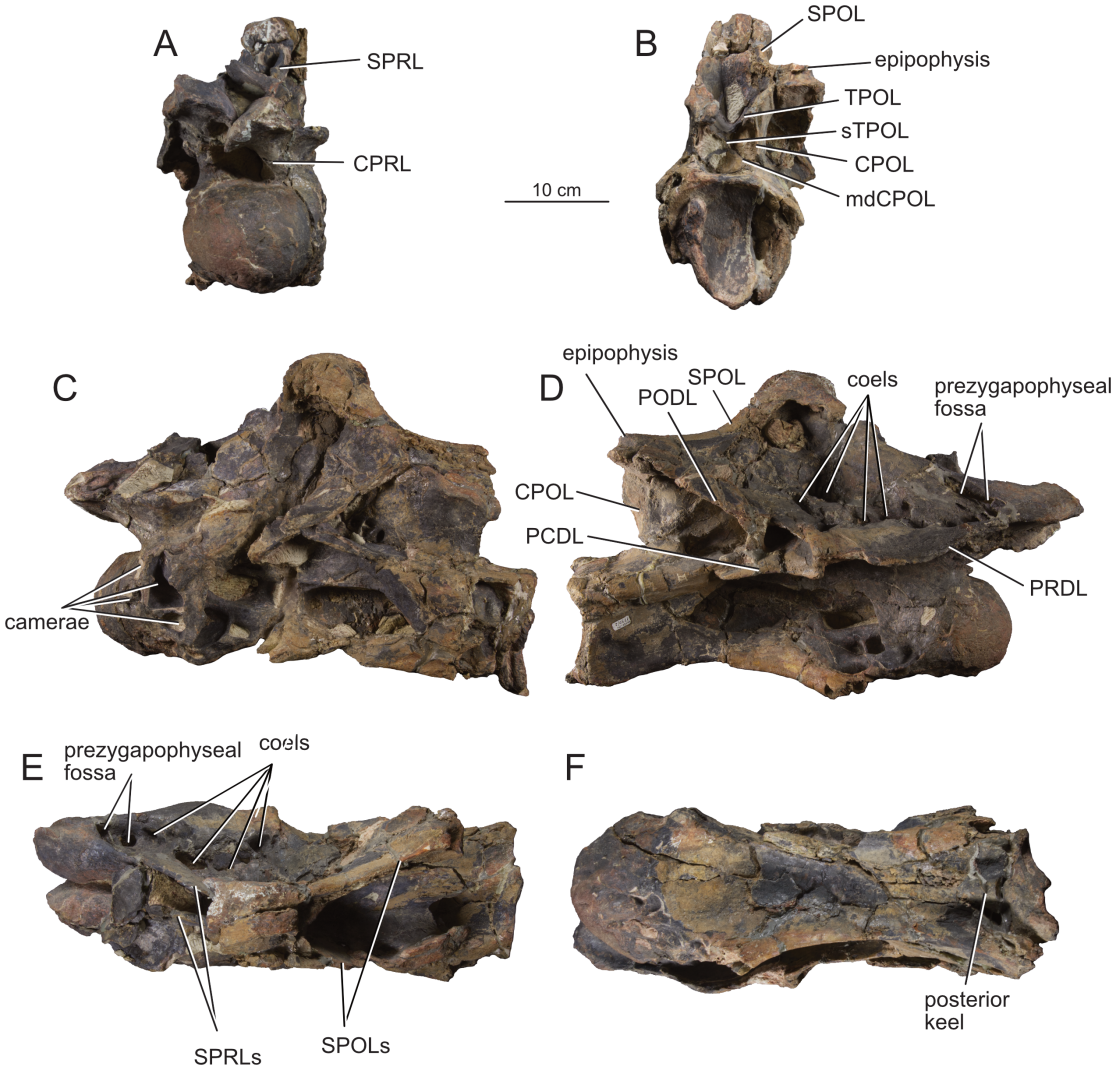
Middle cervical vertebra Ce7 (MPEF-PV 1730/2) of *Bicharracosaurus dionidei*. In (A) anterior; (B) posterior; (C) left lateral; (D); right lateral; (E) dorsal and (F) ventral views. Abbreviations: CPOL, centropostzygapophyseal lamina; CPRL, centroprezygapophyseal lamina; mdCPOL, medial division of the centropostzygapophyseal; PCDL, posterior centrodiapophyseal lamina; PODL, postzygodiapophyseal lamina; PRDL, prezygodiapophyseal lamina; SPOL, spinopostzygapophyseal lamina; SPRL, spinoprezygapophyseal lamina; sTPOL, single interpostzygapophyseal lamina; TPOL, interpostzygapophyseal lamina; TPRL, interprezygapophyseal lamina.

**Figure 6:**
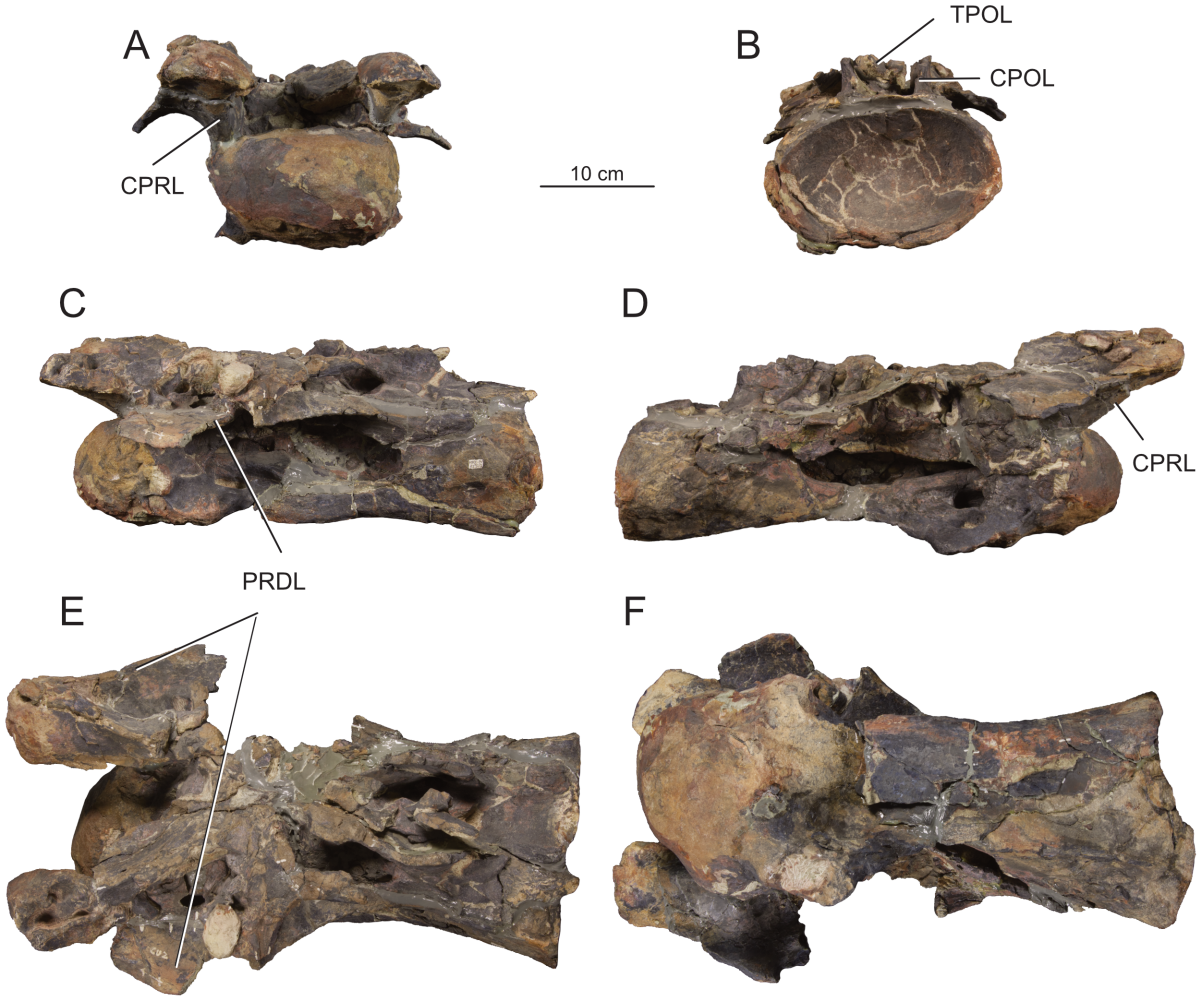
Middle cervical vertebra Ce8 (MPEF-PV 1730/3) of *Bicharracosaurus dionidei*. In (A) anterior; (B) posterior; (C) left lateral; (D); right lateral; (E) dorsal and (F) ventral views. Abbreviations: CPOL, centropostzygapophyseal lamina; CPRL, centroprezygapophyseal lamina; PRDL, prezygodiapophyseal lamina; TPOL, interpostzygapophyseal lamina.

**Figure 7:**
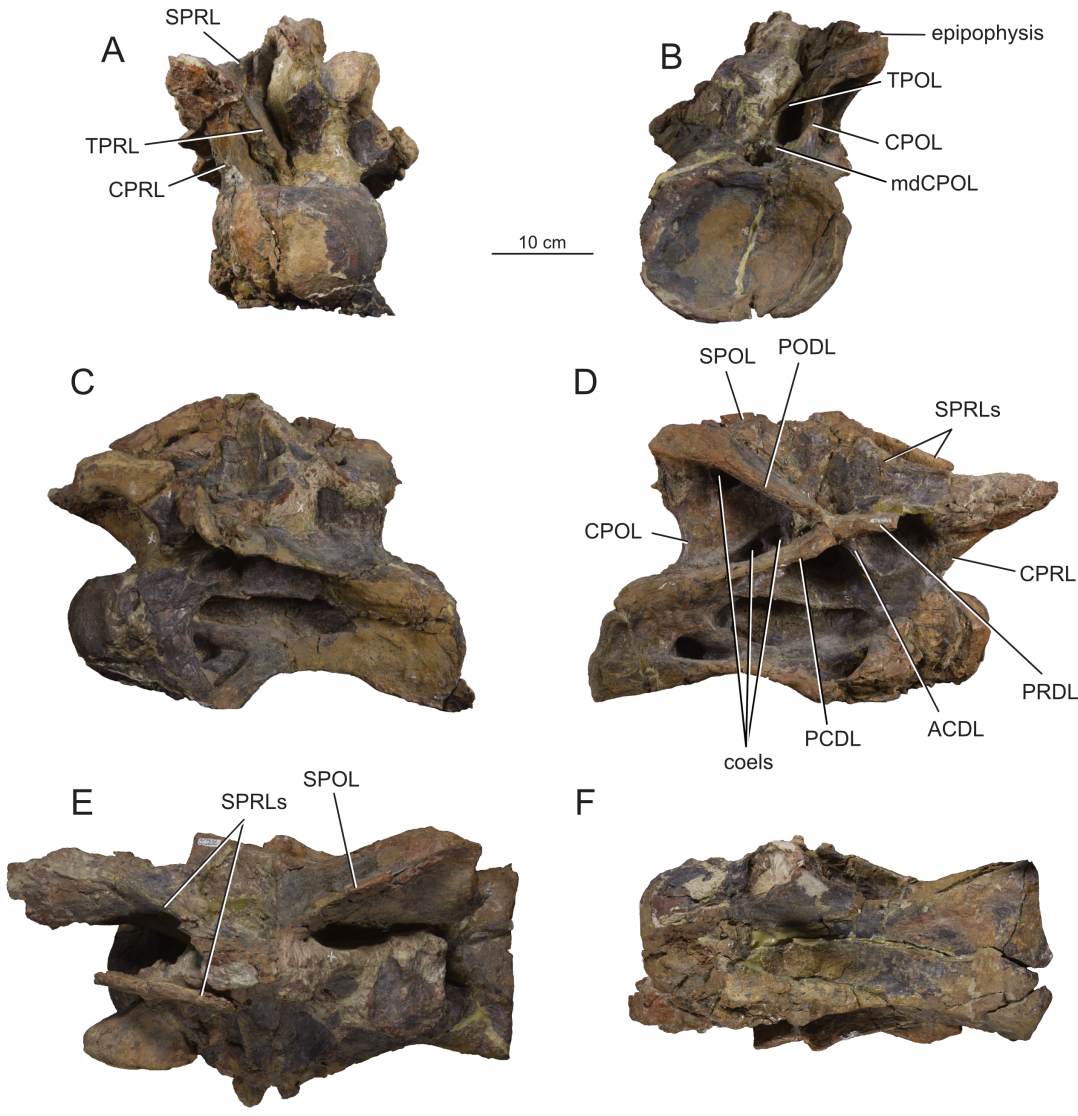
Middle cervical vertebra Ce9 (MPEF-PV 1730/4) of *Bicharracosaurus dionidei*. In (A) anterior; (B) posterior; (C) left lateral; (D); right lateral; (E) dorsal and (F) ventral views. Abbreviations: ACDL, anterior centrodiapophyseal lamina; CPOL, centropostzygapophyseal lamina; CPRL, centroprezygapophyseal lamina; mdCPOL, medial division of the centropostzygapophyseal; PCDL, posterior centrodiapophyseal lamina; PODL, postzygodiapophyseal lamina; PRDL, prezygodiapophyseal lamina; SPOL, spinopostzygapophyseal lamina; SPRL, spinoprezygapophyseal lamina; TPOL, interpostzygapophyseal lamina; TPRL, interprezygapophyseal lamina.

**Figure 8:**
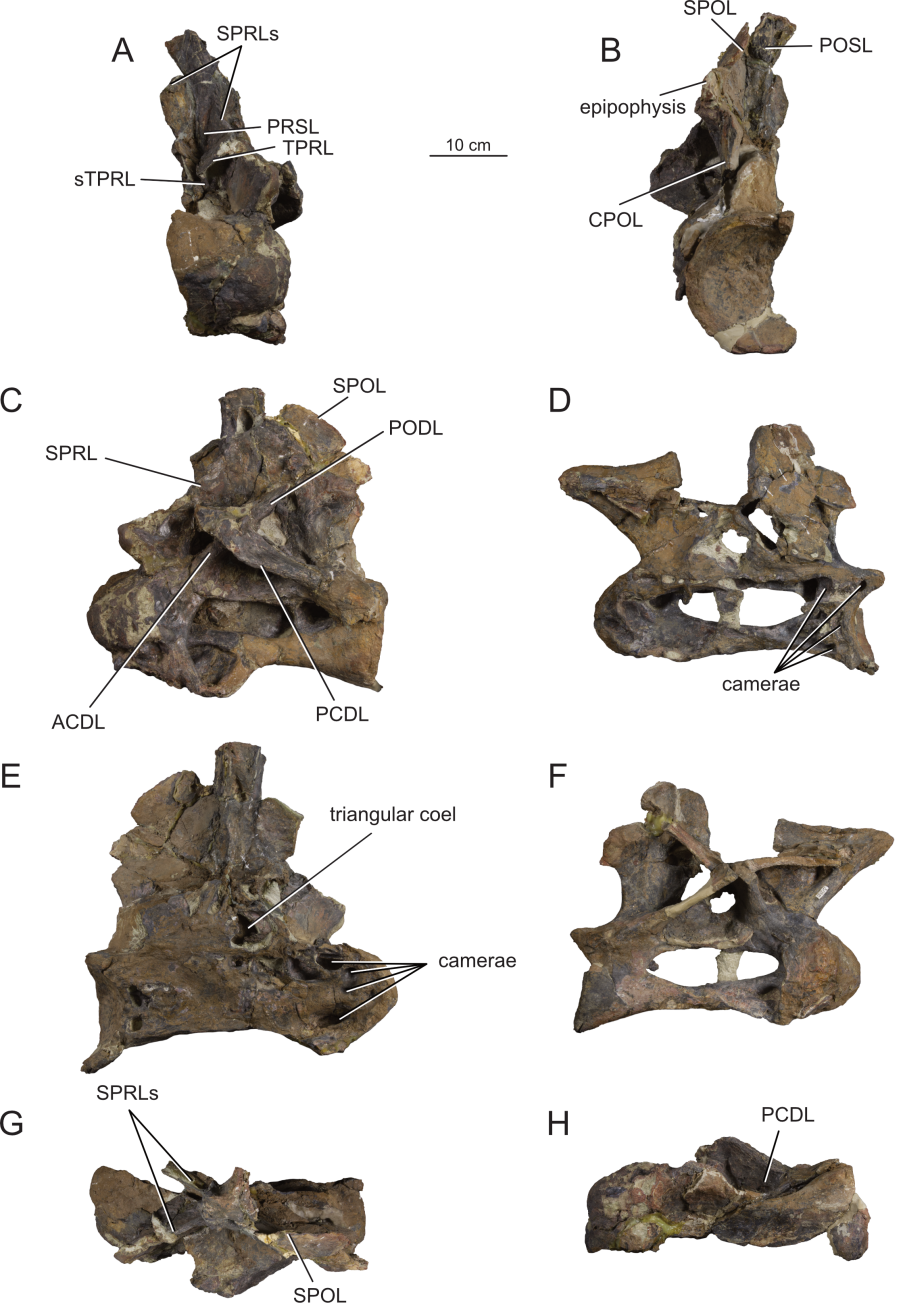
Posterior cervical vertebra Ce10 (MPEF-PV 1730/5) of *Bicharracosaurus dionidei*. In (A) anterior; (B) posterior; (C) left lateral (left half); (D); left lateral (right half); (E) right lateral (left half); (F) right lateral (right half); (G) dorsal and (H) ventral views. Abbreviations: ACDL, anterior centrodiapophyseal lamina; CPOL, centropostzygapophyseal lamina; PCDL, posterior centrodiapophyseal lamina; PODL, postzygodiapophyseal lamina; POSL, postspinal lamina; PRSL, prespinal lamina; SPOL, spinopostzygapophyseal lamina; SPRL, spinoprezygapophyseal lamina; sTPRL, single interprezygapophyseal lamina; TPRL, interprezygapophyseal lamina.

**Figure 9:**
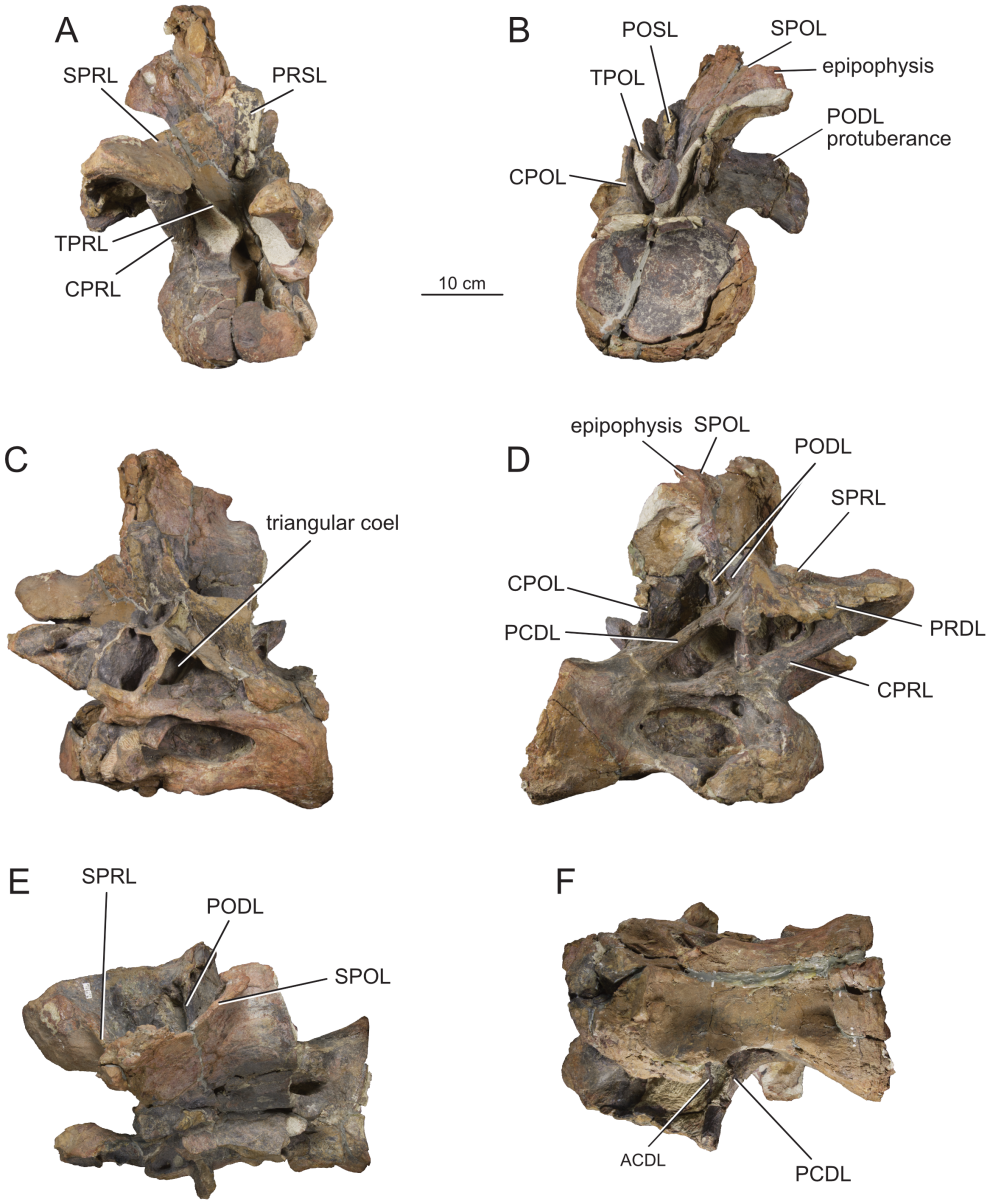
Posterior cervical vertebra Ce11 (MPEF-PV 1730/6) of *Bicharracosaurus dionidei*. In (A) anterior; (B) posterior; (C) left lateral; (D); right lateral; (E) dorsal and (F) ventral views. Abbreviations: CPOL, centropostzygapophyseal lamina; CPRL, centroprezygapophyseal lamina; PCDL, posterior centrodiapophyseal lamina; PODL, postzygodiapophyseal lamina; POSL, postspinal lamina; PRDL, prezygodiapophyseal lamina; PRSL, prespinal lamina; SPOL, spinopostzygapophyseal lamina; SPRL, spinoprezygapophyseal lamina; TPRL, interprezygapophyseal lamina.

**Figure 10:**
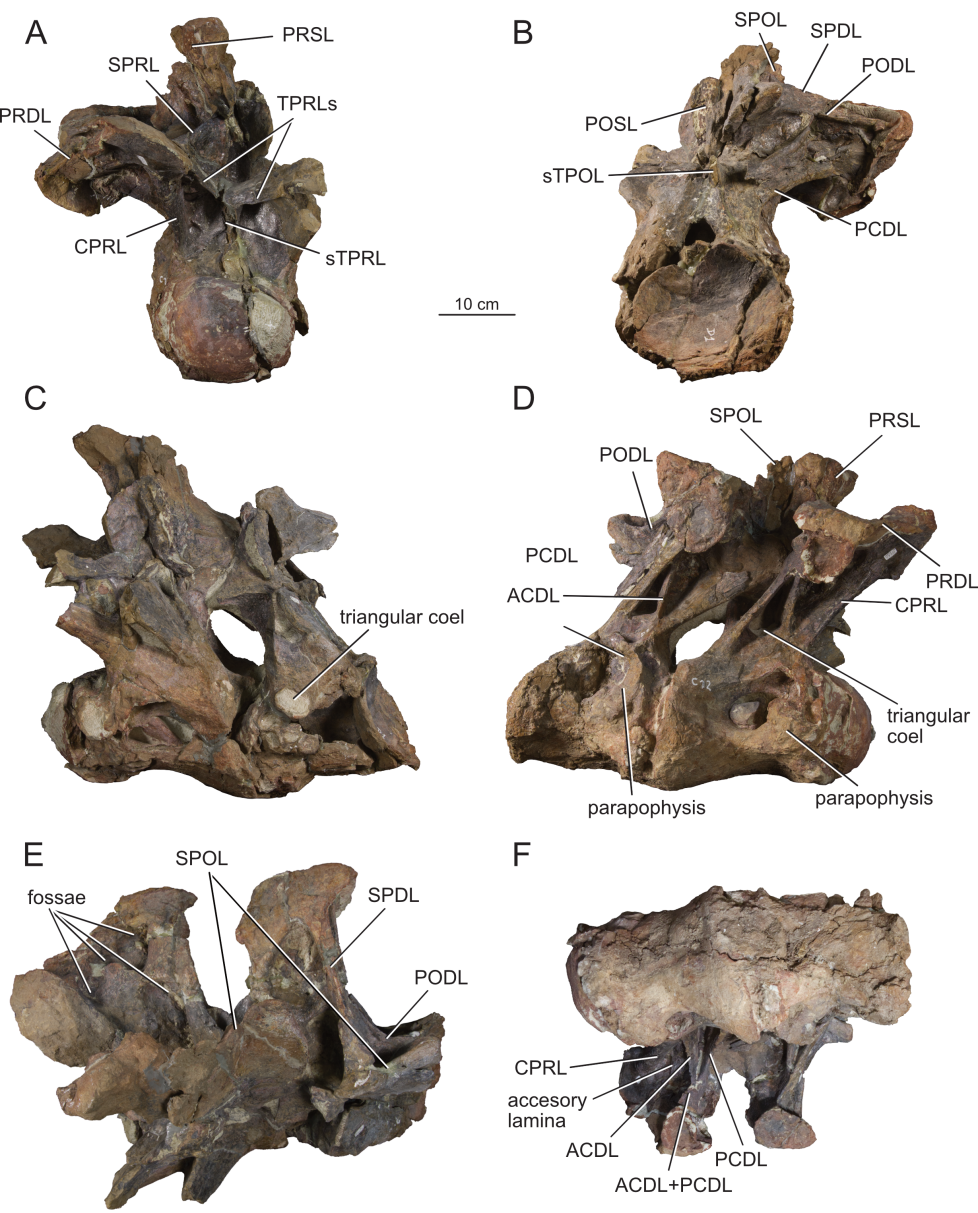
Articulated last cervical Ce12 and first dorsal Do1 vertebrae (MPEF-PV 1730/7) of *Bicharracosaurus dionidei*. In (A) anterior; (B) posterior; (C) left lateral; (D); right lateral; (E) dorsal and (F) ventral views. Abbreviations: ACDL, anterior centrodiapophyseal lamina; CPRL, centroprezygapophyseal lamina; PCDL, posterior centrodiapophyseal lamina; PODL, postzygodiapophyseal lamina; POSL, postspinal lamina; PRDL, prezygodiapophyseal lamina; PRSL, prespinal lamina; SPDL, spinodiapophyseal lamina; SPOL, spinopostzygapophyseal lamina; SPRL, spinoprezygapophyseal lamina; sTPOL, single interpostzygapophyseal lamina; sTPRL, single interprezygapophyseal lamina; TPRL, interprezygapophyseal lamina.

Seven middle to posterior cervical vertebrae (Ce) are preserved (MPEF-PV 1730/1-7; Table 1; Figs. 4–10). These are numbered from Ce6-12, assuming 12 cervicals, as in *Camarasaurus* (Gilmore, 1925; CM 11338: A. Reutter, pers. obs., 2024). Ce6-8 were found disarticulated from each other and the rest of the specimen. Their position within the cervical series was assessed considering centrum length, height and width and neural arch and neural spine height (see Table 1). Based on these measurements and the general taphonomy of the site it is very likely that Ce6-8 represent consecutive vertebrae preceding Ce9. Ce9-10 were found aligned with the rest of the axial skeleton, only some 15 cm apart from Ce11, and were found lying on their left lateral side, while all following presacral vertebrae were found lying on their right lateral side. Ce11-12 were found in articulation with Do1. During preparation Ce11 was separated from Ce12 and Do1. The anteroposterior longest centra are those of Ce8 and Ce9 (Table 1), which are here interpreted as the last middle cervicals. In general, all cervicals are well preserved, except for Ce8, which is slightly dorsoventrally compressed and does not preserve most of the neural arch and spine.

**Table 1:** Measurements (mm) of the cervical vertebrae of *Bicharracosaurus dionidei*. Centrum height and width was measured on the posterior articular surface; centrum length does not include the condyle; neural arch height was measured vertically from the dorsal most point of centrum to the dorsal most point of the postzygapophysis articular surface in posterior view; neural spine height was measured vertically from the dorsal most point of the postzygapophysis articular surface to the dorsal most point of the neural spine in posterior view; neural spine width and length was measured on the dorsal surface.

	**Ce6**	**Ce7**	**Ce8**	**Ce9**	**Ce10**	**Ce11**	**Ce12**
**Centrum height**	145	150^*^	145	150	170	175	170
**Centrum width**	175	155^*^	230	215	200	230	220
**Centrum length**	380	420	440	435	340	310	235
**Condyle length**	60	70	70	60	80	80	90
**Neural arch heigth**	80	120	–	150	160	195	240
**Neural spine height**	90	100	–	85	155	130	145^*^
**Neural spine width**	35	60	–	45	–	–	90
**Neural spine length**	75	80	–	75	–	–	30
**Pleurocoel length**	175	225	190	240	190	140	80

**Notes.**

An asterisk (*) denotes a measurement that is based on an incomplete or deformed element.

All preserved cervical centra are strongly opisthocoelous, as in most sauropods (Salgado, Coria & Calvo, 1997). The posterior articular surfaces of the centra are wider mediolaterally than high dorsoventrally (Table 1), as in several neosauropods, with the exclusion of some titanosaurs and most rebbachisaurs (Mannion et al., 2013). The length of the middle cervical centra increases from Ce6 to Ce8 and then decreases rapidly between Ce9 and Ce10 (Table 1). Similar to most non-Somphospondyli sauropods (Mannion et al., 2019b), the highest average Elongation Index (aEI: the anteroposterior length of centrum (excluding articular ball) divided by the mean average value of the mediolateral width and dorsoventral height of the posterior articular surface of the centrum (Upchurch, 1998; Chure et al., 2010)) is below four. The posterior articulation extends further posteriorly ventrally than dorsally in lateral view. In all but the last cervical, the ventral surface is concave transversely, bound by ventrolateral ridges. Although a ventral keel is absent, as in most non-titanosaurian macronarians (Upchurch, 1998), an anteroposterior short ridge is present in the posterior portion of the centrum in Ce6 and Ce7 (Figs. 4–5F), where it is laterally bound by paired rounded fossae. This morphology is also present in the middle cervicals of *Omeisaurus tianfuensis* (He, Li & Cai, 1988: fig. 21 A) and *Dinheirosaurus lourinhanensis* (Mannion et al., 2012: fig. 2).

In several vertebrae, where the condyle is not well preserved (*e.g.*, Ce6, Ce7 and Ce12), and especially in Ce10 (Figs. 4–5C, 8D–8E), where the vertebra is broken medially, a polycamerate internal system (see Materials and Methods section above) is exposed. Such a system can also be identified in some cervicals of the mamenchisaurid *Omeisaurus puxiani* (Tan et al., 2019: fig. 2), the diplodocids *Diplodocus* and *Apatosaurus* (see Wedel, Cifelli & Sanders, 2000), the macronarians *Galvesaurus herreroi* (MPG CLH3), *Giraffatitan* (Janensch, 1950: fig. 68-71) and also the ambiguously referred cervical vertebrae of *Tendaguria* (Mannion et al., 2019b: fig. 23). This is different from the camerate condition, where the pleurocoel opens into a large internal camera that is not divided further in the centrum, *e.g.*, in *Camarasaurus* (Wedel, Cifelli & Sanders, 2000) and *Europasaurus holgeri* (Carballido & Sander, 2014) and *Pilmatueia faundezi* (Windholz, Coria & Zurriaguz, 2020), and from the camellate internal structure of most mamenchisaurids (He, Li & Cai, 1988; Russell & Zheng, 1993; Ouyang & Ye, 2002; Tan et al., 2021; Moore et al., 2023) and titanosaurs (Wilson & Sereno, 1998; Wedel, 2003). However, the internal morphology of pneumatic structures can only be studied when the internal bone is exposed, as is the case here, or with the use computed tomography. So, to fully understand the evolution of this trait, further study is needed.

The lateral surface of the centra is deeply excavated by anteroposteriorly long, sharp-lipped pleurocoels. The presence of pleurocoels with well-defined edges, including the posterior one, is the widespread condition among neosauropods (Carballido et al., 2012: C114), while a reduced pleurocoel or a shallow fossa is synapomorphic for Titanosauria (Mannion et al., 2013: appendix 4). The pleurocoels are longer anteroposteriorly than higher dorsoventrally; however, unlike in some titanosauriforms (Poropat et al., 2016), they occupy less than two thirds of the centrum length (Table 1). Most cervical pleurocoels of *Bicharracosaurus* are divided, as is common among Neosauropods (Wilson & Sereno, 1998). However, the cervical pleurocoels present a great inter- and intra-variability in their internal divisions. Both pleurocoels in Ce6 have accessory lamina within their anterior portion (Figs. 4C–4D). Likewise, the right pleurocoel of Ce7 shows anterior subdivisions (Fig. 5D), whereas the left pleurocoel has a vertical strut dividing the pleurocoels at mid-length (Fig. 5C). The pleurocoels in Ce8 are undivided, although a dorsally oriented lamina partially subdivides the left pleurocoel (Figs. 6C–6D). The right pleurocoel in Ce9 is divided by an oblique lamina, that is flush with the lateral surface of the centrum, into a main anterior cavity and a smaller posteroventral cavity (Fig. 7D). Within the main cavity, a dorsoventrally oriented ridge, located near to the posterior margin, further divides the pleurocoel. Nevertheless, the left pleurocoel in Ce9 is undivided (Fig. 7C). The internal structure of the right pleurocoel of Ce10 is not preserved, but a dorsoventrally oriented ridge at about two thirds of the length of the pleurocoel divides the left pleurocoel of Ce10 (Fig. 8C). The pleurocoels in Ce11 are undivided. In the last cervical, the right pleurocoel is subdivided by an oblique lamina. Due to preservation, it is not possible to establish if this lamina was present in the left pleurocoel of the vertebra.

The articular surfaces of the parapophyses are not preserved (excluding the posteriormost cervicals, Ce12 and maybe C11), suggesting that the ribs were fused but broken off during preservation. The parapophyses are placed anteriorly on the ventrolateral edges of the centrum. In lateral view, the parapophyses lie anterior to the anterior margin of the pleurocoel. In al cervical vertebrae, except for the last one, the parapophyses are longer than wide, unlike the subcircular parapophyses of *Europasaurus* (Carballido & Sander, 2014: fig. 4C, 7B, 9G, 13B). Nevertheless, their anteroposterior length is less than half the centrum length, contrasting with the exceptionally long parapophyses of *Alamosaurus sanjuanensis* (Lehman & Coulson, 2002: fig. 2.16) and *Saltasaurus loricatus* (PVL 4017-7: A. Reutter, pers. obs., 2023). They project ventrolaterally, but not as ventrally as in *Euhelopus zdanskyi* (Wilson & Upchurch, 2009), *Erketu ellisoni* (Ksepka & Norell, 2006), *Nigersaurus taqueti* (MNN GDA 512: A. Reutter, pers. obs.), *Apatosaurus louisae* (Gilmore, 1936) or *Suuwassea emilieae* (Harris, 2006b), which have very ventrally displaced ribs. The dorsal surface of the parapophyses is excavated, as is widespread in sauropods (*e.g.*, *Jobaria tiguidensis* (MNN TIG 6, 9: A. Reutter, pers. obs., 2024), *Camarasaurus* (BYU 9047: A. Reutter, pers. obs., 2024), *Giraffatitan* (Janensch, 1950: figs. 20, 29, 39, 49), *Ligabuesaurus leanzai* (Bonaparte, González Riga & Apesteguía, 2006: fig. 3A), *Diplodocus* (Hatcher, 1901: plates 3-4) and *Nigersaurus* (MNN GDA 512: A. Reutter, pers. obs., 2024). This parapophyseal fossa is separated from the pleurocoel by an anteroposteriorly extending ridge, as in some neosauropods (Upchurch, 1998), and is subdivided into two or three cavities (*e.g.*, Figs. 6D, 8C).

As with the parapophyses, in all cervicals preceding the cervicodorsal transition, the articular surfaces of the diapophyses are not preserved, indicating that they were fused to the ribs. In Ce12 and Do1, the diapophyses are triangular, with the apex pointing ventrally, have a concave articular surface and are oriented lateroventrally (Figs. 10D, 10F). The articular surfaces have a distinct ventral lip separating them from the remaining transverse process. This indicates that the transitional rib in Ce12 was not fused to the parapophysis nor the diapophysis. A similar morphology is also present the cervicodorsals of *Europasaurus* (Carballido & Sander, 2014: fig. 16), *Euhelopus* (Wilson & Upchurch, 2009: fig. 16) and *Malawisaurus dixeyi* (Gomani, 2005: fig. 11B). Four laminae originate at the transverse process: the ACDL, PCDL, PRDL and PODL. The ACDL is not visible in lateral view in the middle cervicals, where it is short and medially projected. From Ce10 onwards, the ACDL is longer and projects anteroventrally. Similarly, the PCDL is semi-horizontal in middle cervicals, but becomes more vertically than horizontally oriented with the increasing height of the neural arch in posterior cervicals. The PCDL lacks the distinctive posterior triangular flange of *Spinophorosaurus nigerensis* and mamenchisaurids (Remes et al., 2009: fig. 3A). In posteriormost cervicals the ACDL and PCDL meet into a single lamina just ventral to the transverse process, that then extends laterally along the ventral margin of the transverse process until reaching the diapophysis (Fig. 10F). The PRDL maintains a semihorizontal orientation throughout the cervical series. With the lateral expansion of the transverse processes in the posteriormost cervicals, the PRDL remains straight in dorsal view and therefore becomes a vast, sub-horizontal triangular platform (Figs. 9–10F). In contrast, the PODL projects slightly dorsoposteriorly in middle cervicals (Figs. 5D, 7D), and in posterior cervicals (Fig. 9D) and then sub-horizontal in the last cervical (Fig. 10D). In these vertebrae, the PODL has an hourglass shape in posterior view (Fig. 9B).

In posterior cervicals (Ce10-Ce11) the PODL bifurcates ventrally (Fig. 9D). As pointed out by Moore et al. (2020: fig. 6A, C, 33) this is also present in the posterior cervicals of *Klamelisaurus gobiensis*, *Euhelopus* (Wilson & Upchurch, 2009: fig. 12), an isolated vertebrae of the Phu Kradung Formation of Thailand (Suteethorn et al., 2012: fig. 3-4 B) and the ‘Shishugou cervicodorsal vertebrae’ (Moore et al., 2020). We also found this morphology in the posterior cervicals of *Spinophorosaurus* (GCP-CV-4229), *Haplocanthosaurus priscus* (Hatcher, 1903: plate 1; CM 572: A. Reutter, pers. obs., 2024) and *Dinheirosaurus* (Mannion et al., 2012: fig. 2). Following Moore et al. (2020) it is different from the widely diverging accessory lamina present in posterior cervicals of *Brontosaurus parvus* (Gilmore, 1936: plate 31) and *Galeamopus pabsti* (Tschopp & Mateus, 2017: figs. 26–27, 31).

A deep triangular coel invades the dorsal margin of the CDF, just below the transverse process, in all preserved cervicals (Figs. 8E, 9C, 10D), which is here interpreted as an autapomorphy of *Bicharracosaurus*. This coel is internally connected to the pleurocoel. Both the PRCDF, delimited by ACDL, PRDL and CPRL, and the POCDF, delimited by the PCDL, PODL and CPOL, are deeply excavated in middle and posterior cervicals. In the posteriormost cervical of *Bicharracosaurus* the PRCDF is divided by a vertically oriented accessory lamina (Fig. 10F). In the right lateral aspect of Ce9, several fossae are present along the ventral margin (or the PCDL) of the POCDF (Fig. 7D). There is also a rounded coel just below the postzygapophyses (or the PODL). In Ce11 and Ce12 the distal portion of the PODL has a unique small dorsally oriented protuberance (Fig. 9B). In Ce12, the dorsal surface of the transverse process is pierced by several distinct fossae separated by ridges (Fig. 10E).

The articular surfaces of the prezygapophyses face dorsomedially. In middle cervicals the articular surfaces are anteroposteriorly longer than mediolaterally wide, contrary to the case in posterior cervicals. In all cervicals the articular surfaces of the prezygapophyses are flat to slightly convex, unlike the strongly convex articular facets of *Diplodocus* and *Barosaurus lentus* (Upchurch, 1995), *Xinjiangtitan shanshanesis* (Zhang et al., 2020) and *Hudiesaurus sinojapanorum* (Upchurch et al., 2021: fig. 2C). The prezygapophyses extend far beyond the condyle, as is the plesiomorphic condition among sauropods (Salgado, Coria & Calvo, 1997). The typical middle cervical lateral prezygapophyseal fossa of flagellicaudatans (Harris, 2006a; Harris, 2006b: figs. 5–7 B–D), that is also present in *Europasaurus* (Carballido & Sander, 2014: *e.g.*, figs. 7B–C, 8B) and *Giraffatitan* (Janensch, 1950: fig. 29), is present in Ce6 and Ce7 of *Bicharracosaurus* (Figs. 4, 5D–5E). A pre-epipophysis, a blunt process below the prezygapophysis (Wilson & Upchurch, 2009), is present in the last cervical of *Bicharracosaurus* (Figs. 10D–10E), as is widespread within Eusauropoda (Mannion et al., 2013). The prezygapophyses are connected to the centrum through paired, undivided CPRL, unlike the divided CPRL of some diplodocoids (Upchurch, 1998). The CPRLs are oriented vertically and contact the centrum on its anterolateral margin. The prezygapophyses are also supported by ventromedially oriented TPRL that contact each other at the dorsal margin of the neural canal. A sTPRL is especially developed in the last cervical of *Bicharracosaurus* (Fig. 10A), where the neural arch is highest (Table 1). These laminae, CPRL, TPRL and sTPRL, form the margins of paired deep fossa (CPRF) on the anterior surface of the neural arch, dorsolateral to the neural canal (Figs. 8–10A).

While the neural arch is lower than the dorsoventral height of the posterior articular surface in middle cervicals, it is higher in posterior cervicals (see Table 1). Low neural arches (ratio of less than 1.0) in middle cervical vertebrae were found as a synapomorphy uniting *Mamenchisaurus* and *Omeisaurus* (Wilson, 2002: p. 267). High posterior cervical neural arches (ratio of 0.5 or greater) are the typical condition amongst non-lithostrotian sauropods (Bonaparte, González Riga & Apesteguía, 2006; Mannion et al., 2013).

The articular surfaces of the postzygapophyses are facing ventrolaterally. The articular surfaces are flat to slightly concave transversely. The postzygapophyses do not project posteriorly beyond the posterior margin of the centrum. The epipophyses are pillar-like, in contrast to the derived prong-like epipophyses that are present in several early branching eusauropods, especially developed in *Jobaria* (MNN TIG 6, 9: A. Reutter, pers. obs., 2024), diplodocoids such as *Suuwassea* (Harris, 2006a; Harris, 2006b: *e.g.*, figs. 6D, 8D) and *Nigersaurus* (MNN GDA 512: A. Reutter, pers. obs., 2024), and in some derived macronarians (Ksepka & Norell, 2006; You et al., 2008; Wilson & Upchurch, 2009). The postzygapophyses are supported from below by paired CPOL and TPOL. The CPOL are oriented vertically and contact the centrum at the level of the lateral margin of the neural canal. Therefore, the mediolateral distance separating both CPOL is less than the distance between both CPRL. The CPOL bifurcate dorsally, with the lateral branch contacting the postzygapophyses and the dorsomedially directed branch contacting the TPOL or sTPOL at the dorsal margin of the neural canal (mdCPOL) (Figs. 4–5B, 7B). The TPOLs are oriented ventromedially and contact each other at the dorsal margin of the neural canal, forming a short sTPOL. This lamina was first identified by Upchurch & Martin (2002: fig. 3B) for the cervical vertebrae of *Cetiosaurus oxoniensis*, stating that there is an excavated area on either side of the lamina (CPOF). This morphology was then also recognized in *Europasaurus* by Carballido & Sander (2014: fig. 8C, 9B–D, 12 B and 13C). These authors interpreted that, as a result of these fossa (CPOF), the CPOL becomes divided dorsally into a mdCPOL. Thus, the presence of a sTPOL in middle and/or posterior cervical vertebrae is generally accompanied by the presences of the mdCPOL. The presence of these CPOFs or mdCPOLs is widespread among sauropods, such as *Mamenchisaurus youngi* (Ouyang & Ye, 2002: fig. 18B, D), *Jobaria* (MNN TIG 6, 9: AR pers. obs. 2024), *Camarasaurus* (Osborn & Mook, 1921: fig. 34)*, Euhelopus* (PMU R 233), *Giraffatitan* (Janensch, 1950: fig. 19, 22, 25, 28, 31), *Erketu* (Ksepka & Norell, 2006: figs. 4E, 5E), in the juvenile *Rapetosaurus krausei* (Curry Rogers, 2009: figs. 9–10B, 13B), and in diplodocoids such as *Haplocanthosaurus priscus* (Hatcher, 1903: plate 1), *Diplodocus carnegii* (Hatcher, 1901: plate 6), *Apatosaurus louisae* (Gilmore, 1936: plate 14), *Lingwulong shenqi* (Xu et al., 2018: suppl. fig. 4C), *Dicraeosaurus* (Janensch, 1929: plate 1) and *Brachytrachelopan* (MPEF-PV 1716: A. Reutter, pers. obs., 2023).

The cervical neural spines are undivided in all preserved vertebrae of *Bicharracosaurus* contrary to flagellicaudatans (Tschopp, Mateus & Benson, 2015), some mamenchisaurids (*e.g.*, Ouyang & Ye, 2002; Upchurch et al., 2021), and macronarians like *Camarasaurus* and *Euhelopus* (Osborn & Mook, 1921; Gilmore, 1925; McIntosh et al., 1996; Wilson & Sereno, 1998; Wilson & Upchurch, 2009). The neural spines are anteroposteriorly longer than mediolaterally wide and have parallel to slightly converging lateral margins in the middle cervicals. This changes in posterior cervicals where the neural spines are mediolaterally wider than anteroposteriorly long and have slightly diverging lateral margins, similar to the morphology in *Haplocanthosaurus priscus* (CM 572: A. Reutter, pers. obs., 2024). This is different from the strongly diverging neural spines with a convex dorsal margin of some titanosaurs (Calvo et al., 2007; González Riga, Previtera & Pirrone, 2009. In Ce11 the left lateral side of the neural spine is missing, but the right one is well preserved (Fig. 9A). In Ce12 the neural spine is preserved with the anterior side directed anterolaterally (Figs. 10A, 10E). This neural spine was probably crushed into that position before diagenesis. In middle cervicals the neural spines are low, unlike the high neural spines of dicraeosaurids (Rauhut et al., 2005). Also, there is an abrupt increase in neural spine height, as was identified for *Sauroposeidon proteles*, *Giraffatitan, Erketu* and *Paluxysaurus jonesi* (Wedel, Cifelli & Sanders, 2000: fig. 10). In *Bicharracosaurus* this change occurs between Ce9 and Ce10 (Table 1). In lateral view, the anterior and posterior margins of the middle cervical neural spines have an equivalent slope. In posterior cervicals the neural spines show a steeply inclined anterior margin and a less inclined posterior margin, as is the case for most eusauropods (Upchurch, 1998).

Anteriorly, the neural spines are formed by paired SPRLs that emerge from the lateral side of the prezygapophyses and reach the dorsal margin of the neural spine. In the middle cervicals of *Bicharracosaurus* the SPRLs make up the complete anterior margin of the neural spine in lateral view, whereas in the posterior cervicals the SPRLs reach the neural spines at around midheight and then continue towards the dorsal margin of the neural spine on its lateral surface. This change in morphology of the SPRLs contributes to the increasing verticality of the anterior margin in posterior neural spines. Only posterior cervicals have wide and rough PRSLs (Figs. 8–10A). While the presence of a PRSL in posterior cervicals and anterior dorsals is synapomorphic for Somphospondyli (D’Emic, 2012: C33) it is also present in *Europasaurus* (Carballido & Sander, 2014: fig. 12, 14, 16). Posteriorly, the neural spine is bound by paired SPOLs. These laminae emerge from the epipophyses and reach the dorsal margin of the neural spine. In lateral view the SPOLs form the complete posterior margin of the neural spine even in the posteriormost cervicals. In middle cervicals the angle between the SPOL and PODL is acute (Figs. 4C, 5–6D) unlike the obtuse angle of dicraeosaurids (Rauhut et al., 2005: C67). POSLs are also only present in the posterior cervicals (Figs. 8–10B). In Ce7 the SDF is pierced by several rounded coels separated by low ridges (Fig. 5D), as in the middle cervicals of *Vouivria damparisensis*, *Giraffatitan* and referred cervicals of *Brachiosaurus* (see Mannion, Allain & Moine, 2017: p. 34–35). In most of the preserved cervical neural spines of *Bicharracosaurus*, the neural spine has a laterodorsal rugose ridge, as in other macronarians such as *Jobaria* (MNN TIG 6, 8: A. Reutter, pers. obs., 2024), *Camarasaurus* (SMA 0002: A. Reutter, pers. obs., 2023), *Europasaurus* (Carballido & Sander, 2014: fig. 12A), *Giraffatitan* (Janensch, 1950: fig. 29, 37, 43), *Vouivria* (Mannion, Allain & Moine, 2017: fig. 10A) and *Euhelopus* Wilson & Upchurch, 2009.

#### Cervical ribs

**Figure 11:**
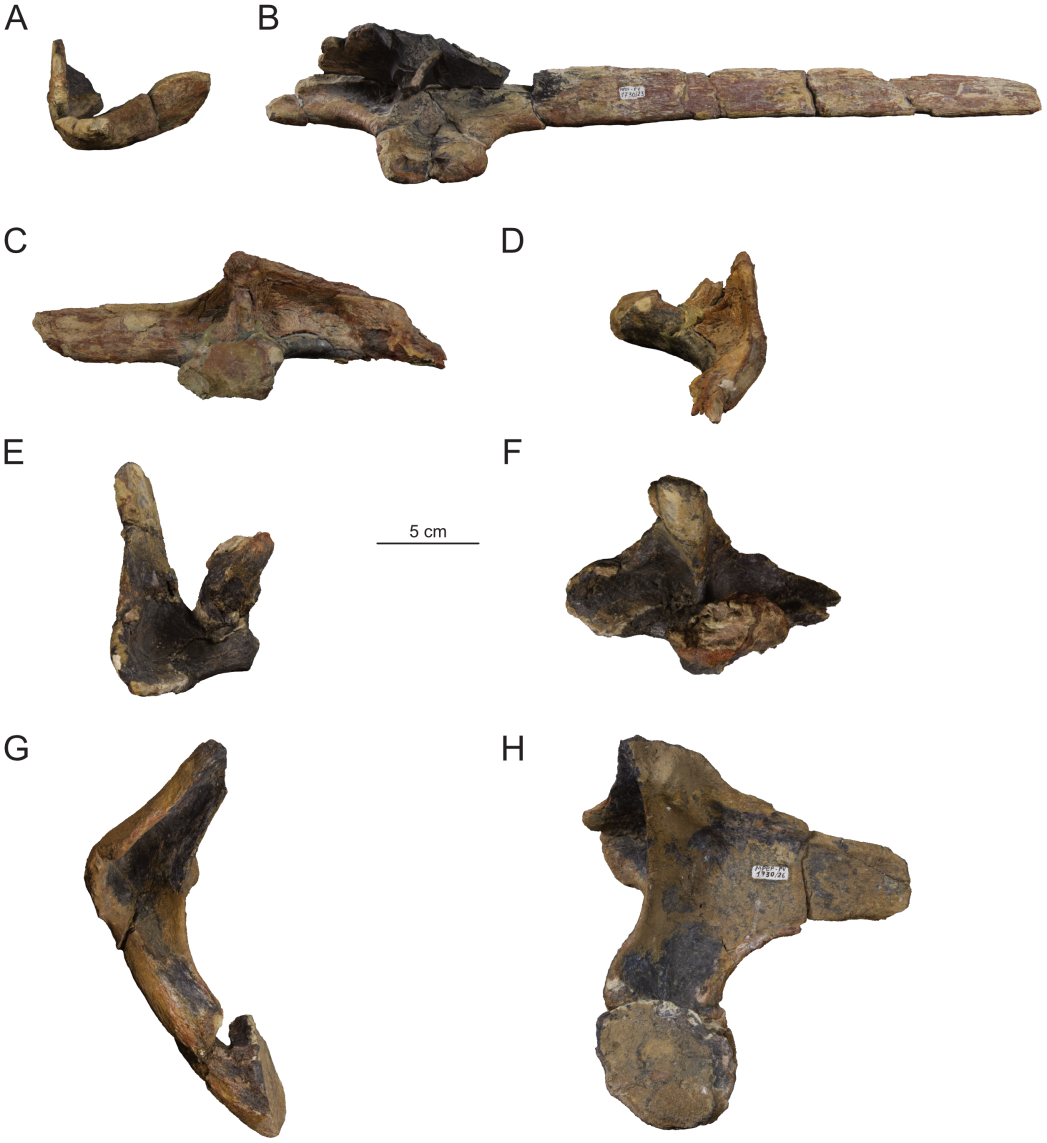
Cervical ribs (MPEF-PV 1730/23-26) of *Bicharracosaurus dionidei*. (A) MPEF-PV 1730/23 in anterior and (B) left dorsolateral; (C) MPEF-PV 1730/24 in right dorsolateral and (D) anterior; (E) MPEF-PV 1730/25 in anterior and (F) left dorsolateral; (G) MPEF-PV 1730/26 in anterior and (H) left dorsolateral views.

A total of four cervical ribs were found (MPEF-PV 1730/23-26; Fig. 11), but none of them was found in articulation with the vertebrae. Not much information can be gained from these elements as none is completely preserved, with all lacking the tips of the anterior and most of the posterior processes. The most complete cervical rib (MPEF-PV 1730/23) is the least robust element and is probably an anterior to middle rib. MPEF-PV 1730/24 and MPEF-PV 1730/25 are more robust elements. These ribs are here interpreted as middle cervical ribs. MPEF-PV 1730/26 has an intermediate shape between a cervical and a dorsal rib and is here interpreted a posteriormost cervical rib. Based on its proportions it probably articulated with Ce11 or Ce12.

The capitulum is anteroposteriorly longer than dorsoventrally high in the middle cervicals and is rounded in the transitional rib (Fig. 11H). The angle between the capitulum and tuberculum is close to 80° in the middle cervical ribs, so that the ribs lie ventral but close to the ventral margin of the centrum (Figs. 11A, 11D–11E) unlike the strongly ventrally displaced ribs of some diplodocoids and titanosaurs (Wilson & Sereno, 1998; Wilson & Upchurch, 2009; Mannion et al., 2013). The posteriormost rib has an obtuse angle and the shaft is displaced dorsally at the height of the arch (Fig. 11G). The posterior process of MPEF-PV 1730/23 is not completely preserved, but it would extend posterior to the posterior margin of the centrum even in articulation with the longest cervical (Ce8), unlike the short ribs of diplodocoids (Upchurch, 1995: p. 381).

In contrast with apatosaurines (*e.g.*, *Apatosaurus ajax*; Gilmore, 1936) an anterior process is present even in the posterior cervical ribs of *Bicharracosaurus* (Figs. 11G–11H). In MPEF-PV 1730/24, the anterior process is completely preserved (Fig. 11C). Its anterior extension would not surpass the anterior margin of the centrum even in articulation with the most anteriorly preserved middle cervical (Ce6). This is the common condition among sauropods, with the exception of mamenchisaurids (*e.g.*, *Omeisaurus tianfuensis;* He, Li & Cai, 1988: figs. 20–21). The anterior process lacks the bifurcation seen in some Late Jurassic Chinese sauropods (Moore et al., 2023: fig. 6). The dorsomedial aspect of the ribs, between the capitulum and tuberculum, is concave, with a vertical lamina demarcating a deep anterior and a less deep posterior fossa. This lamina originates from the anterior aspect of the capitulum and reaches the midline of the tuberculum. In the posteriormost cervical (MPEF-PV 173/26) this lamina and the fossae are particularly well developed. Such a lamina is common among sauropods (*e.g.*, *Bellusaurus*; Moore et al., 2023: fig. 6H, *Galeamopus*; Tschopp & Mateus, 2017: fig. 40, and *Diamantinasaurus matildae*; Poropat et al., 2015: fig. 4).

#### Dorsal vertebrae

**Figure 12:**
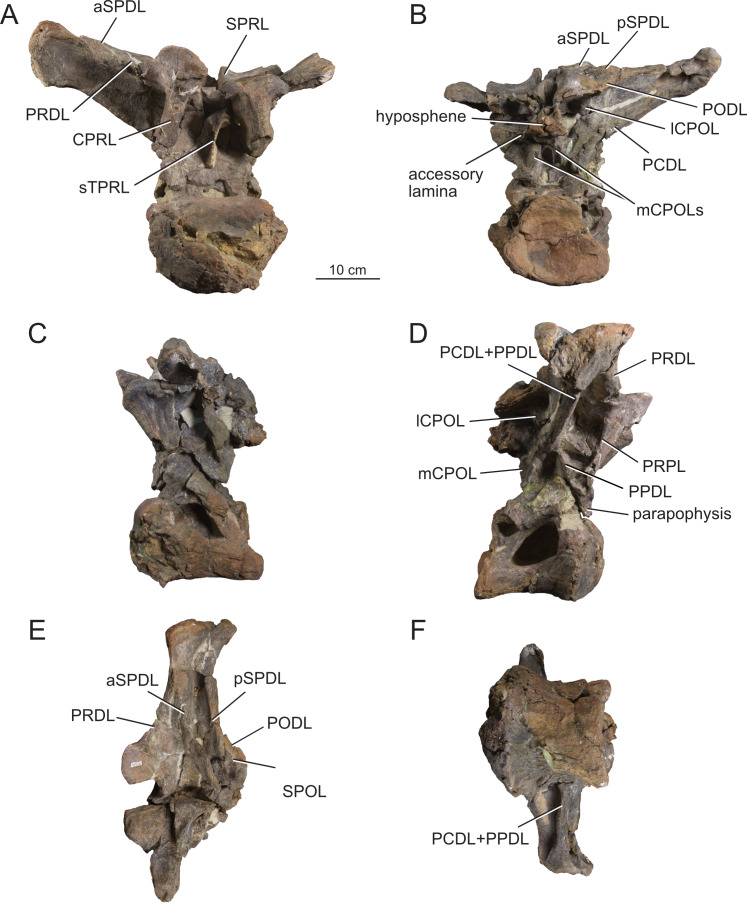
Anterior dorsal vertebra Do2 (MPEF-PV 1730/8) of *Bicharracosaurus dionidei*. In (A) anterior; (B) posterior; (C) left lateral; (D) right lateral; (E) dorsal and (F) ventral views. Abbreviations: aSPDL, anterior spinodiapophyseal lamina; CPRL, centroprezygapophyseal lamina; lCPOL, lateral centropostzygapophyseal lamina; mCPOL, medial centropostzygapophyseal lamina; PCDL, posterior centrodiapophyseal lamina; PODL, postzygodiapophyseal lamina; PPDL, paradiapophyseal lamina; PRDL, prezygodiapophyseal lamina; PRPL, prezygoparapophyseal lamina; pSPDL, posterior spinodiapophyseal lamina; SPOL, spinopostzygapophyseal lamina; SPRL, spinoprezygapophyseal lamina; sTPRL, single interprezygapophyseal lamina.

**Figure 13:**
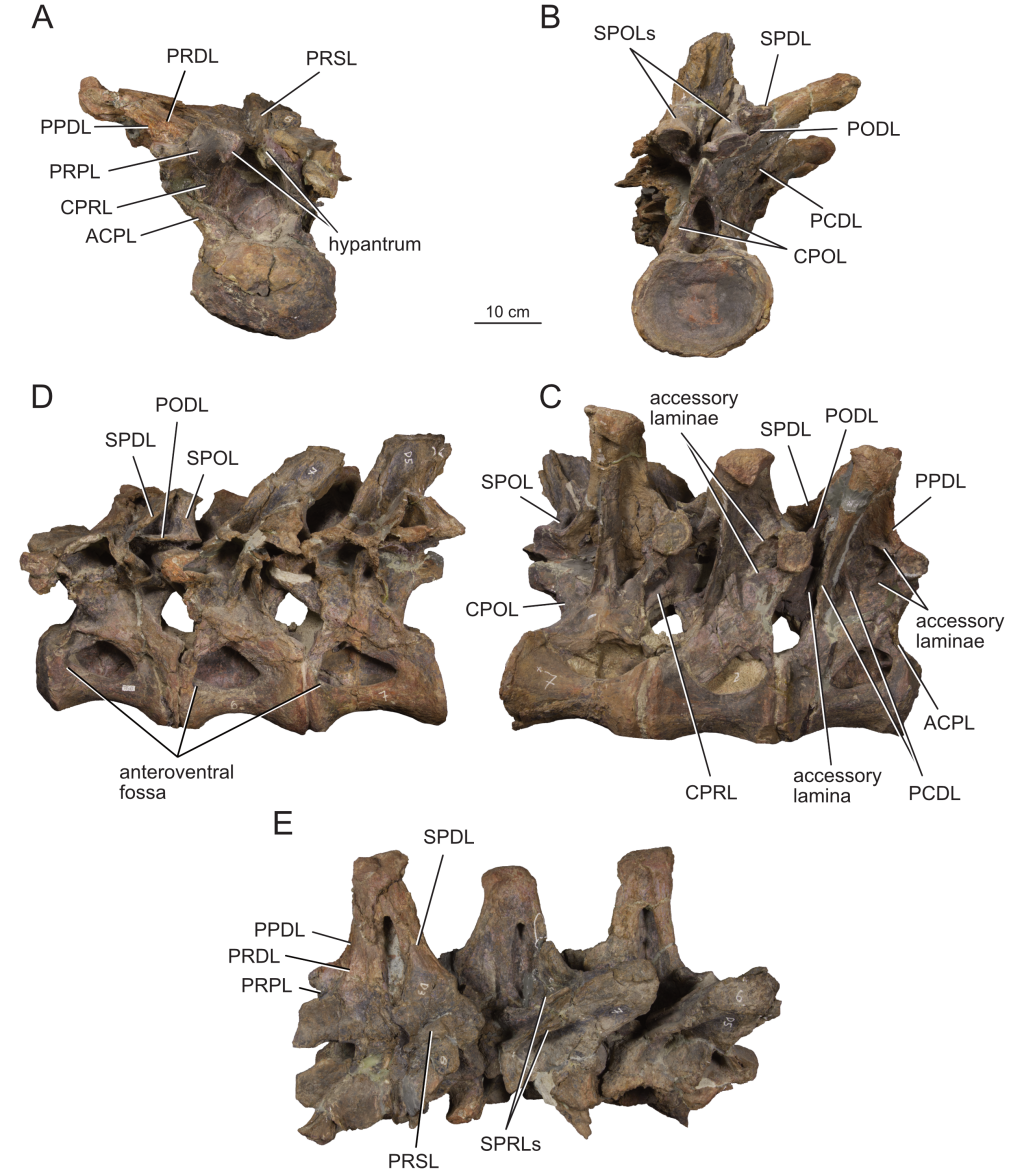
Middle dorsal vertebrae Do3-5 (MPEF-PV 1730/9) of *Bicharracosaurus dionidei*. In (A) anterior; (B) posterior; (C) left lateral; (D) right lateral and (E) dorsal views. Abbreviations: CPL, anterior centroparapophyseal lamina; CPOL, centropostzygapophyseal lamina; CPRL, centroprezygapophyseal lamina; PCDL, posterior centrodiapophyseal lamina; PODL, postzygodiapophyseal lamina; PPDL, paradiapophyseal lamina; PRPL, prezygoparapophyseal lamina; PRSL, prespinal lamina; SPDL, spinodiapophyseal lamina; SPOL, spinopostzygapophyseal lamina; SPRL, spinoprezygapophyseal lamina.

**Figure 14:**
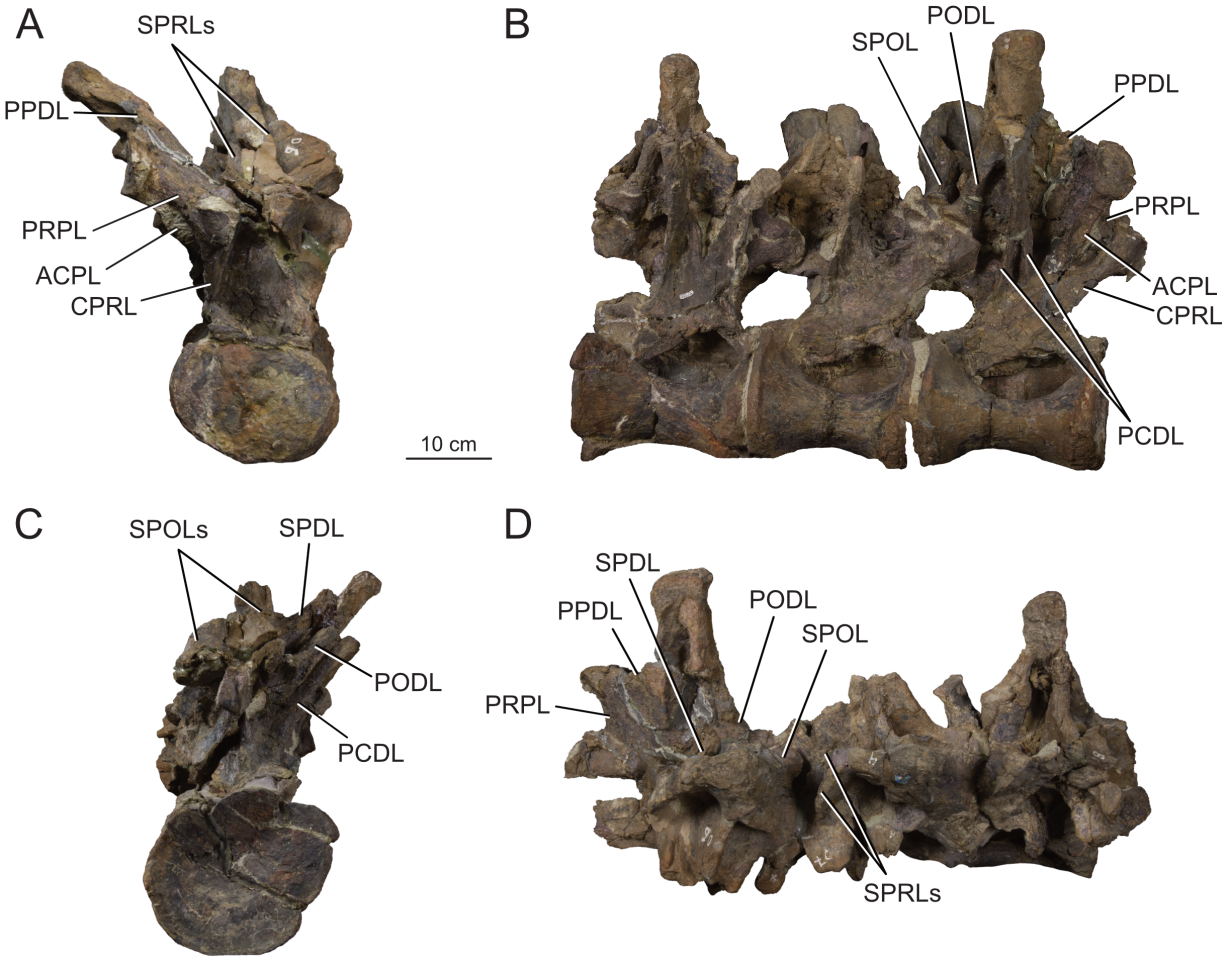
Middle and posterior dorsal vertebrae Do6-8 (MPEF-PV 1730/10) of *Bicharracosaurus dionidei*. In (A) anterior; (B) right lateral; (C) posterior and (D) dorsal views. Abbreviations: ACPL, anterior centroparapophyseal lamina; CPRL, centroprezygapophyseal lamina; PCDL, posterior centrodiapophyseal lamina; PODL, postzygodiapophyseal lamina; PPDL, paradiapophyseal lamina; PRPL, prezygoparapophyseal lamina; SPDL, spinodiapophyseal lamina; SPOL, spinopostzygapophyseal lamina; SPRL, spinoprezygapophyseal lamina.

**Figure 15:**
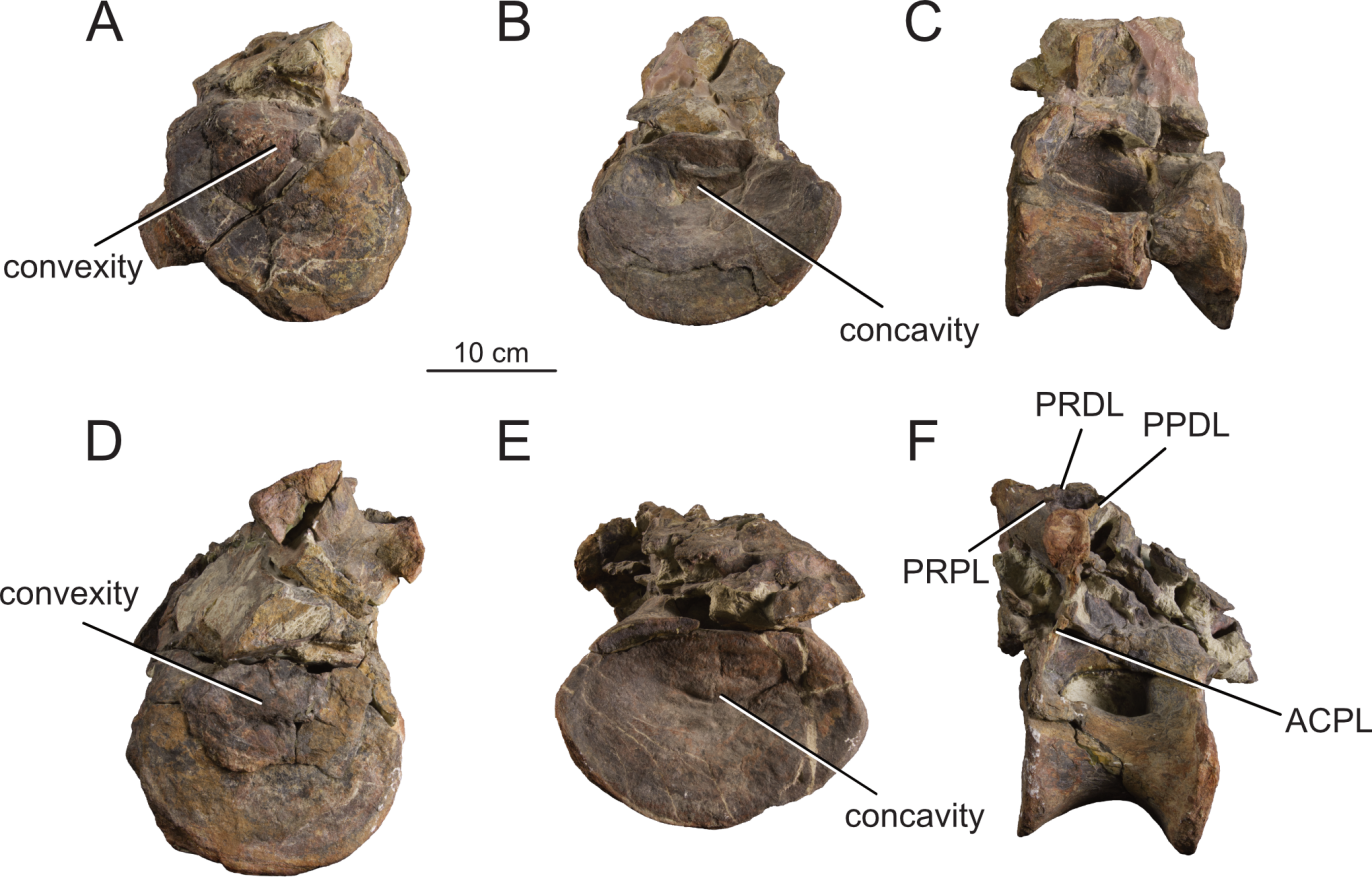
Posterior dorsal vertebrae Do9-10 (MPEF-PV 1730/11-12) of *Bicharracosaurus dionidei*. Do9 in (A) anterior; (B) posterior and (C) left lateral; Do10 in (D) anterior; (E) posterior and (F) left lateral views. Abbreviations: ACPL, anterior centroparapophyseal lamina; PPDL, paradiapophyseal lamina; PRDL, prezygodiapophyseal lamina; PRPL, prezygoparapophyseal lamina.

The complete dorsal series of *Bicharracosaurus* (MPEF-PV 1730/7-12; Table 2; Figs. 10, 12–15) is preserved, representing a total of 10 vertebrae (Do1-Do10), unlike *Tehuelchesaurus,* which has at least 11 (Carballido et al., 2011b). We mainly focus our description on the right lateral side as it is better preserved, except in the posteriormost dorsals. The first dorsal was found in articulation with the last two cervicals, while the rest of the dorsals were found in articulation with the sacrum. During field work, Do2 was separated from the rest of the dorsal column. Do3 through Do8 were extracted together and during preparation separated between Do5 and Do6. Do9 and Do10 were collected separately and preserve mainly the centrum. The neural spine is at least partially preserved in Do3-Do8.

**Table 2:** Measurements (mm) of the dorsal vertebrae of *Bicharracosaurus dionidei*. Measurements follow the same procedure as in cervical vertebrae (see Table 1).

	**D1**	**D2**	**D3**	**D4**	**D5**	**D6**	**D7**	**D8**	**D9**	**D10**
**Centrum height**	160	145^*^	165	175	170	180	185	200	170	180
**Centrum width**	200	170^*^	205	210	205	205	210	240	220	270
**Centrum length**	205	175^*^	205	210	215	215	190	205	195	180
**Neural arch heigth**	210	165	185	180	165	135	160	150	–	–
**Neural spine height**	–	–	–	205	185	175	165^*^	–	–	–
**Neural spine width**	–	–	–	60	60	55	35	–	–	–
**Neural spine length**	–	–	–	90	110	100	130	–	–	–
**Pleurocoel length**	–	80	110	105	135	120	135	120	125^*^	105

**Notes.**

An asterisk (*) denotes a measurement that is based on an incomplete or deformed element.

In general, macronarians, including *Tehuelchesaurus*, tend to have strongly opisthocoelous dorsal centra, even in the posterior elements (Calvo & Salgado, 1995; Upchurch, Barrett & Dodson, 2004; Carballido et al., 2011b). In *Bicharracosaurus*, the degree of convexity of the anterior articular surface is greatly reduced across the dorsal series, whereby the anterior articular surface is strongly convex in anterior dorsals, but slightly so in posterior dorsal vertebrae. The posterior dorsal vertebrae of *Bicharracosaurus* have a slightly convex dorsal portion in the anterior articular surface and a dorsally placed concavity in the posterior articular surface (Figs. 15A–15B, 15D–15E). As noted by Mannion, Tschopp & Whitlock (2021: fig. 6) this slightly opisthocoelous condition is common among diplodocoids, such as *Diplodocus carnegii* (Hatcher, 1901: plate 7; CM 84: A. Reutter, pers. obs., 2024), *Apatosaurus louisae* (Gilmore, 1936: plate 25), *Barosaurus* (McIntosh, 2005: fig. 2.5), *Amphicoelias altus* (Mannion, Tschopp & Whitlock, 2021: fig. 5 D) and *Haplocanthosaurus priscus* (Hatcher, 1903: plates 1-2; CM 572: A. Reutter, pers. obs., 2024). Among macronarians it has been described for the brachiosaurids *Lusotitan atalaiensis* (Mannion et al., 2013: fig. 2; Mocho, Royo-Torres & Ortega, 2017), *Vouivria* (Mannion, Allain & Moine, 2017: fig. 13A), possibly in *Soriatitan golmayensis* (Royo-Torres et al., 2017a: fig. 3A), and is also present in the last dorsal vertebra of *Brachiosaurus* (Taylor, 2009: fig. 1 N; FMNH P 25107: A. Reutter, pers. obs., 2024). Some turiasaurs also present this morphology (*e.g.*, *Mierasaurus bobyoungi* Royo-Torres et al., 2017b: figs. 4E–G; UMNH.VP.26004: A. Reutter, pers. obs., 2024). Likewise, the best-preserved dorsal centrum of MPEF-PV 1324 is slightly opisthocoelous. In this specimen, however, the maximum convexity is placed centrally, instead of dorsally.

As in the cervical vertebrae, the posterior articular surfaces of the dorsal centra are wider mediolaterally than high dorsoventrally. This morphology is especially pronounced in the last dorsals (Table 2). In these vertebrae, the posterior articular surface is also much wider than the anterior articular surface (Figs. 15A–15B, 15D–15E). The length of the first dorsal centrum is similar to the following vertebrae, with the posteriormost dorsal vertebrae decreasing only slightly in length (Table 2). The ventral surfaces are convex mediolaterally, unlike the concave ventral surfaces of some titanosaurs (Poropat et al., 2016). A ventral midline keel is absent, which is the typical condition in most sauropods (Upchurch, Barrett & Dodson, 2004). In lateral view the ventral margin of the centrum is concave anteroposteriorly. The internal morphology of the centrum is exposed in the condyle of Do2. A polycamerate system, as in the cervical vertebrae of *Bicharracosaurus*, is absent. Instead, the pleurocoels do not subdivide further within the centra (camerate) as in most non-titanosauriform and non-dicraeosaurid neosauropods (Mannion et al., 2013).

The pleurocoels deeply excavate the lateral surface of the centra in *Bicharracosaurus,* as in most non-dicraeosaurid eusauropods (Upchurch, Barrett & Dodson, 2004). Except for Do1, where the parapophyses are at the level of the pleurocoels, the latter are longer anteroposteriorly than high dorsoventrally and are not divided, unlike those of some titanosaurs and diplodocoids (Salgado, Coria & Calvo, 1997; Mannion et al., 2012). As in other macronarians, the posterior margins of the pleurocoels are acute (Mannion et al., 2013). While in anterior and middle dorsals the pleurocoels are placed anteriorly on the centrum, in posterior dorsals they occupy almost the entire length of the centrum, leaving equally short distances on both ends (Figs. 15C, 15F). This is different from *Tehuelchesaurus* where the pleurocoels are relatively smaller. The dorsal margin of the pleurocoels is high on the centrum, approximately level with the ventral floor of the neural canal, and is also acutely angled in middle and posterior dorsals (Figs. 13–14B, 15C). Both of these morphologies were previously identified in *Apatosaurus louisae*, *Diplodocus carnegii* and MPEF-PV 1324 (Rauhut, Carballido & Pol, 2015), but have a wider distribution, being also present in *Moabosaurus utahensis* (Britt et al., 2017: fig. 3), *Haplocanthosaurus priscus* (Hatcher, 1903: plate 1; CM 572: A. Reutter, pers. obs., 2024), *H. delfsi* (McIntosh & Williams, 1988: fig. 8; CMNH 10380: A. Reutter, pers. obs., 2024), *Barosaurus* (McIntosh, 2005: fig. 2.5), *Camarasaurus* (McIntosh et al., 1996: plate 5; SMA 0002, BYU 9047: A. Reutter, pers. obs., 2023, 2024) and *Europasaurus* (Carballido & Sander, 2014: figs. 17, 21–22; DFMMh/FV 712.1, 1195: A. Reutter, pers. obs., 2024). On the contrary, the pleurocoels in the middle to posterior dorsal vertebrae of *Tehuelchesaurus* are lower on the centrum and have an oval outline (Carballido et al., 2011b: figs. 8–10, 13), similar to those of *Brachiosaurus* (Riggs, 1904: plate 72; FMNH P 25107: A. Reutter, pers. obs., 2024) and *Giraffatitan* (Janensch, 1950: fig. 6, 65). In *Bicharracosaurus* a small triangular fossa, anteroventral to the pleurocoel, is present in middle to posterior dorsals. So far, this fossa has only been described for MPEF-PV 1324 and *Diplodocus* (Rauhut, Carballido & Pol, 2015).

During the cervicodorsal transition the parapophyses move quickly dorsally. In Do1, the right parapophysis, which is better preserved, is placed within the anterior end of the pleurocoel at mid-heigh of the centrum. In this vertebra, the parapophysis is moon-shaped, being as high as the pleurocoel and half as long as it is high (Fig. 10D). In Do2, the parapophyses are not well preserved, but they are placed just above the pleurocoel (Figs. 12C–12D). The CPRL and ACDL present in the preceding vertebra are here considered as PRPL and PPDL respectively. At this point no other parapophyseal laminae are present. In Do3, the parapophysis is at the level of the prezygapophysis, the CPRL reappears and the ACPL support the parapophyses from below, as usual in sauropods (Wilson, 2002). A PCPL, as in known for many sauropods (Upchurch, 1998), is absent in the dorsals of *Bicharracosaurus* and *Tehuelchesaurus*. As the parapophyses reach a position above the prezygapophyses by Do4, the PRDL is completely replaced by the PRPL and PPDL (Fig. 13E), only to reappear in the posteriormost dorsal vertebra (Fig. 15F). Since in *Tehuelchesaurus* the parapophyses remain low, below the prezygapophyses, the PRDL is present even in posterior dorsals in this taxon. In addition, the PRPL is absent in middle and posterior dorsals of *Tehuelchesaurus*. A high parapophysis, in relation to the prezygapophysis, was first recognized in some rebbachisaurs (Wilson & Allain, 2015), but has since also been identified in the turiasaurs *Moabosaurus* (Britt et al., 2017: fig. 4; BYU 10976: A. Reutter, pers. obs., 2024) and *Mierasaurus bobyoungi* (Royo-Torres et al., 2017b: fig. 4; UMNH.VP.26004: A. Reutter, pers. obs., 2024) as well as in *Tastavinsaurus* (Canudo, Royo-Torres & Cuenca-Bescós, 2008: fig. 3C). The high position of the parapophyses in *Bicharracosaurus* allows the CPRL to be visible in lateral view, while in *Tehuelchesaurus* it remains behind the ACPL and is reduced, or even absent (although more detailed examination is hindered because the region is partially covered in matrix).

In the anteriormost dorsals the parapophyses are directed laterally in *Bicharracosaurus* but lateroventrally in *Tehuelchesaurus* (Carballido et al., 2011b: figs. 3–4). In the middle and posterior dorsals of *Bicharracosaurus* the parapophyses are subcircular, being slightly higher dorsoventrally than they are long and are excavated from posterior. This excavation is related to an accessory lamina of the parapophysis, which is also present in *Tehuelchesaurus.* This lamina originates at the dorsal margin of the parapophysis and is directed posteriorly or posterodorsally and therefore is not to be confused with the PCPL. In *Tehuelchesaurus* this lamina is well-developed and present from the fifth until at least the ninth dorsal vertebra (Carballido et al., 2011b: autapomorphic lamina one). In *Bicharracosaurus* this lamina is not as well-developed and is only present from the third to the fourth dorsal vertebra (Fig. 15B). However, in *Tehuelchesaurus* this lamina meets another accessory lamina (autapomorphic lamina two) while in *Bicharracosaurus* the lamina fades into the parapophyseal centrodiapophyseal fossa. The neural arch is shifted anteriorly in the first dorsal. This morphology has been found to be related with an upwards curvature between the thorax and the neck (Britt et al., 2024).

As mentioned before, in the cervicodorsal transition (Ce12 and Do1) the diapophyses have an expanded, triangular flat articular surface that is oriented lateroventrally. While in anterior dorsals the transverse processes project laterally and only slightly dorsally (Figs. 12A–12B), in middle and posterior dorsals they change to a more dorsolaterally projection (Figs. 13–14A, 14C) as is common in several sauropods, including *Jobaria* (MNN TIG 9: A. Reutter, pers. obs., 2024), *Tastavinsaurus* (Canudo, Royo-Torres & Cuenca-Bescós, 2008: figs. 3A–B) and *Giraffatitan* (Taylor, 2009).

In middle and posterior dorsals the PCDL of *Bicharracosaurus* is ventrally bifurcate, as in several Somphospondyli and *Tehuelchesaurus* (Carballido et al., 2011b: figs. 6–10). Do1 is the first vertebra to bear a SPDL which is present until the posterior dorsals. Do2 is the only vertebra, where the SPDL is divided into an anterior and posterior ramus, as in some titanosaurs (Salgado & Powell, 2010), *Haplocanthosaurus priscus* (Hatcher, 1903: plate 1; CM 572: A. Reutter, pers. obs., 2024) and *Comahuesaurus windhauseni* (Carballido et al., 2012: figs. 2-3). Between both laminae, the dorsal surface of the transverse process is deeply excavated (Fig. 12E). Even though the SPDL is not divided in subsequent vertebrae, a similar excavation is still present (Fig. 13E). The SPDL (or pSPDL in Do2) contacts the neural spine at its base, merging with the SPOL in all dorsal vertebrae, as in most sauropods (Wilson & Sereno, 1998). Together with the PODL, the SPDL and SPOL enclose a low triangular POSDF that laterally excavates the postzygapophyses (Figs. 13–14C).

The articular surfaces of the prezygapophyses in anterior and middle dorsals of *Bicharracosaurus* are slightly mediodorsally oriented, unlike those of some titanosaurs and diplodocoids (Poropat et al., 2016). Contrary to rebbachisaurs (Apesteguía, Gallina & Haluza, 2010) the prezygapophyses in *Bicharracosaurus* are separated from each other by a TPRL. A sTPRL is still present until Do2 (Fig. 12A), but is absent in middle and posterior vertebrae, with the emergence of the hypantrum. As mentioned above, the CPRL is visible in lateral view. The SPRLs are present throughout the cervical and dorsal series, except for Do3 (Fig. 13A). These paired laminae do not converge at the base of the spine, as it is the case in some titanosaurs and diplodocoids (Poropat et al., 2016).

As in the cervical vertebrae, the postzygapophyses are supported from below by paired CPOLs in the dorsal vertebrae, except for Do1, where the CPOLs are absent or not preserved (Fig. 10B). With the emergence of the hyposphene in Do2, the CPOLs remain in a medial position, supporting the hyposphene from below (Figs. 12B, 13C), as in most sauropods (Apesteguía, 2005) (*e.g.*, the Rutland *Cetiosaurus*; Upchurch & Martin, 2002: figs. 5D–F, in some vertebrae of *Giraffatitan*; Janensch, 1950: fig. 66; MB.R.3822, MB.R.3824: A. Reutter, pers. obs., 2023, 2024, *Brachiosaurus*; Taylor, 2009: fig. 1A, E; FMNH P 25107: A. Reutter, pers. obs., 2024 and in *Chubutisaurus insignis*; Carballido et al., 2011a: fig. 3E). Do2 is the only vertebra where the CPOLs are divided, with additional paired lateral branches below the postzygapophyses. However, in contrast to *Cetiosaurus* and *Chubutisaurus*, in *Bicharracosaurus* the CPOLs are undivided in middle and posterior dorsals.

The TPOL is absent in all dorsals, and a sTPOL in only present in Do1, where no hyposphene is present (Fig. 10B). In *Tehuelchesaurus* the hyposphene is supported from below only by a sTPOL, which was originally identified in *Diplodocus* and *Barosaurus* (Upchurch, 1995), but has since also been found in more basal forms, such as *Tazoudasaurus naimi* (Allain & Aquesbi, 2008: figs. 14C–D) and *Patagosaurus fariasi* (Holwerda, Rauhut & Pol, 2021: figs. 13–14D, 15E). Furthermore, the paired CPOLs in *Tehuelchesaurus* are in a lateral position. An accessory horizontal lamina connecting the hyposphene and the PCDL, as in *Dinheirosaurus* and other diplodocids (Bonaparte & Mateus, 1999; Mannion et al., 2012: Fig. 3; Tschopp, Mateus & Benson, 2015), is also present in middle dorsal vertebrae of *Bicharracosaurus* (Figs. 12–13B). The SPOLs are undivided, as in *Galvesaurus* (Sánchez-Hernández, 2005: fig. 2C), *Tehuelchesaurus*, *Europasaurus* (Carballido & Sander, 2014: figs. 18–19, 21–22) and *Tastavinsaurus* (Canudo, Royo-Torres & Cuenca-Bescós, 2008: figs. 2–3).

The middle and posterior dorsal single neural spines of *Bicharracosaurus* possess sub-parallel lateral margins, unlike rebbachisaurs and dicraeosaurs (Upchurch, 1998). However, contrary to most macronarians (Wilson & Sereno, 1998), aliform processes are absent in neural spines of *Bicharracosaurus.* Only a few other macronarians (*e.g.*, *Tehuelchesauru* s; *Paluxysaurus;* Rose, 2007: fig. 14; ‘Cloverly titanosauriform’ YPM 5449; D’Emic & Foreman, 2012: figs. 6A–D) share this morphology with *Bicharracosaurus*. While in Do3-5 the ventral projection of the anterior margin of the neural spine is level with the diapophysis, in Do6-8 it is anterior to it. The height of the neural spines in relation to the height of the centrum is much lower (see Table 2) than in diplodocoids (Upchurch, 1998). The neural spines are slightly dorsoposteriorly projecting, but not as strongly as in some titanosaurus (Wilson, 2002). The posterior dorsal neural spines of *Bicharracosaurus* are transversely narrower than they are anteroposteriorly long. This is an uncommon reversion to the plesiomorphic condition of Sauropodomorpha, which is shared with *Omeisaurus puxiani* (Tan et al., 2021), *Spinophorosaurus* (Remes et al., 2009), *Jobaria* (Sereno et al., 1999), *Galvesaurus* (Pérez-Pueyo et al., 2019), and *Tehuelchesaurus*.

A rugose and wide PRSL is present in the dorsals of *Bicharracosaurus,* as in several basal macronarians, such as *Europasaurus* (Carballido & Sander, 2014: figs. 16A, 18–21A) and *Galvesaurus* (Sánchez-Hernández, 2005: fig. 2A) but not *Camarasaurus lewisi* (BYU 9047: A. Reutter, pers. obs., 2024), *Tehuelchesaurus* (Carballido et al., 2011b: fig. 11) and *Tastavinsaurus* (Canudo, Royo-Torres & Cuenca-Bescós, 2008: fig. 3A). Posteriorly, the POSL is absent as is also the case in *Tehuelchesaurus* (Carballido et al., 2011b: fig. 11).

**Figure 16:**
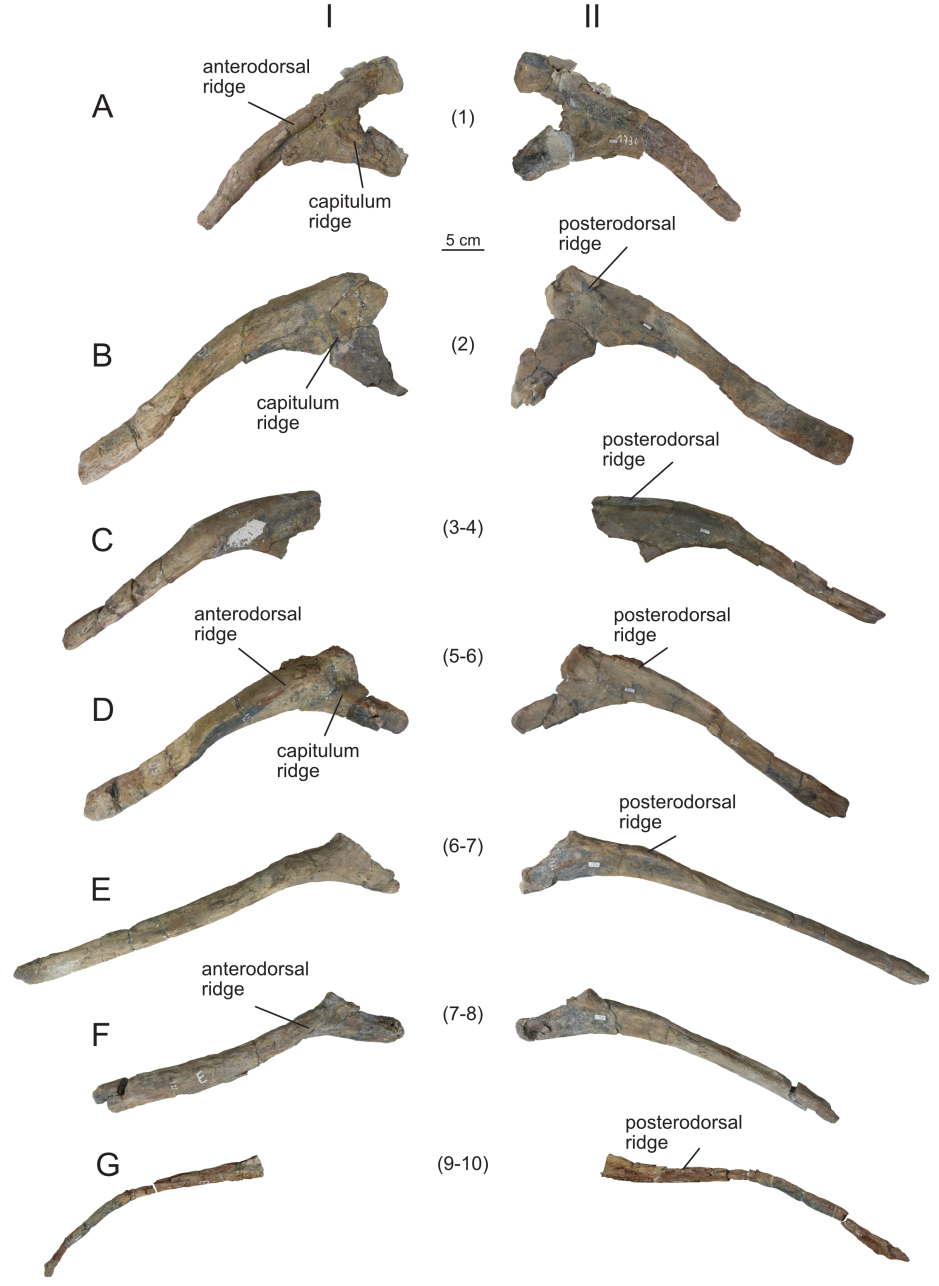
Right dorsal ribs (MPEF-PV 1730/29-30, 32-33, 35-36, 38) of *Bicharracosaurus dionidei*. (A) MPEF-PV 1730/29; (B) MPEF-PV 1730/30; (C) MPEF-PV 1730/32; (D) MPEF-PV 1730/33; (E) MPEF-PV 1730/35; (F) MPEF-PV 1730/36; (G) MPEF-PV 1730/38 in (I) anterior and (II) posterior views. Numbers in parenthesis indicate the approximate position in dorsal series.

**Figure 17:**
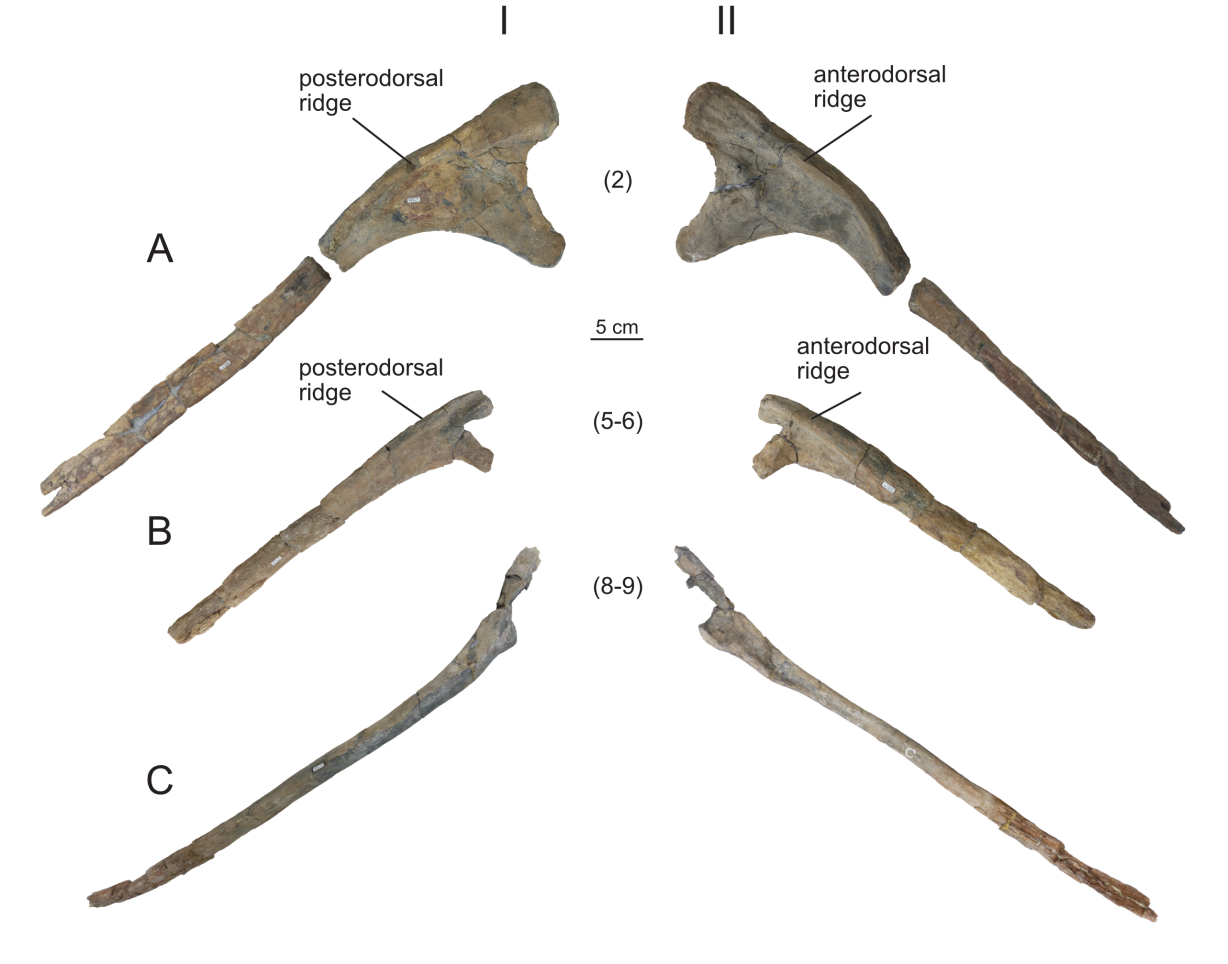
Left dorsal ribs (MPEF-PV 1730/31, 34, 37) of *Bicharracosaurus dionidei*. (A) MPEF-PV 1730/31; (B) MPEF-PV 1730/34; (C) MPEF-PV 1730/37 in (I) posterior and (II) anterior views. Numbers in parenthesis indicate the approximate position in dorsal series.

#### Dorsal ribs

A total of 10 dorsal ribs were recovered (MPEF-PV 1730/29-38; Figs. 16–17). As in the cervical vertebrae none of them were found in articulation with the vertebrae. Their approximate position within the dorsal series was established based on their morphology and that of the vertebrae, considering the size of the articular surfaces of the capitulum/parapophysis and tuberculum/diapophysis, the distance and angle between the capitulum and tuberculum/parapophysis and diapophysis and the shape of the shaft. None of the ribs is completely preserved distally.

Since the parapophysis is low in the first two dorsal vertebrae, the capitulum lies vertically bellow the tuberculum in the anterior dorsal ribs but is in an anteroventral position in middle and posterior dorsal ribs. While in anterior dorsal ribs the angle between the capitulum and tuberculum is around 90°, this angle becomes acute in middle and posterior dorsal ribs, with the capitulum extending further medially than the tuberculum (Figs. 16–17). The anterior proximal surface is flat in the anterior dorsal ribs and slightly convex in the middle dorsal ribs. An anterodorsal ridge, extending from the tuberculum to the shaft, is present in most ribs, while the ridge of the capitulum, extending only the length of the capitulum, is lost in posterior dorsal ribs (Fig. 16). Similarly, the posterior proximal surface is flat in the anterior dorsal ribs and becomes concave in the middle and posterior dorsal ribs. A posterodorsal ridge is present along the entire sequence. Proximal pneumatic foramina are absent in the ribs of *Bicharracosaurus*, unlike in titanosauriform sauropods (Wilson & Sereno, 1998). The shafts of anterior dorsal ribs are plank-like as in in most macronarians (Wilson, 2002), while in posterior elements the shafts become rounded, especially so in the last dorsal rib (Figs. 16E–16G).

#### Sacral vertebrae

**Figure 18:**
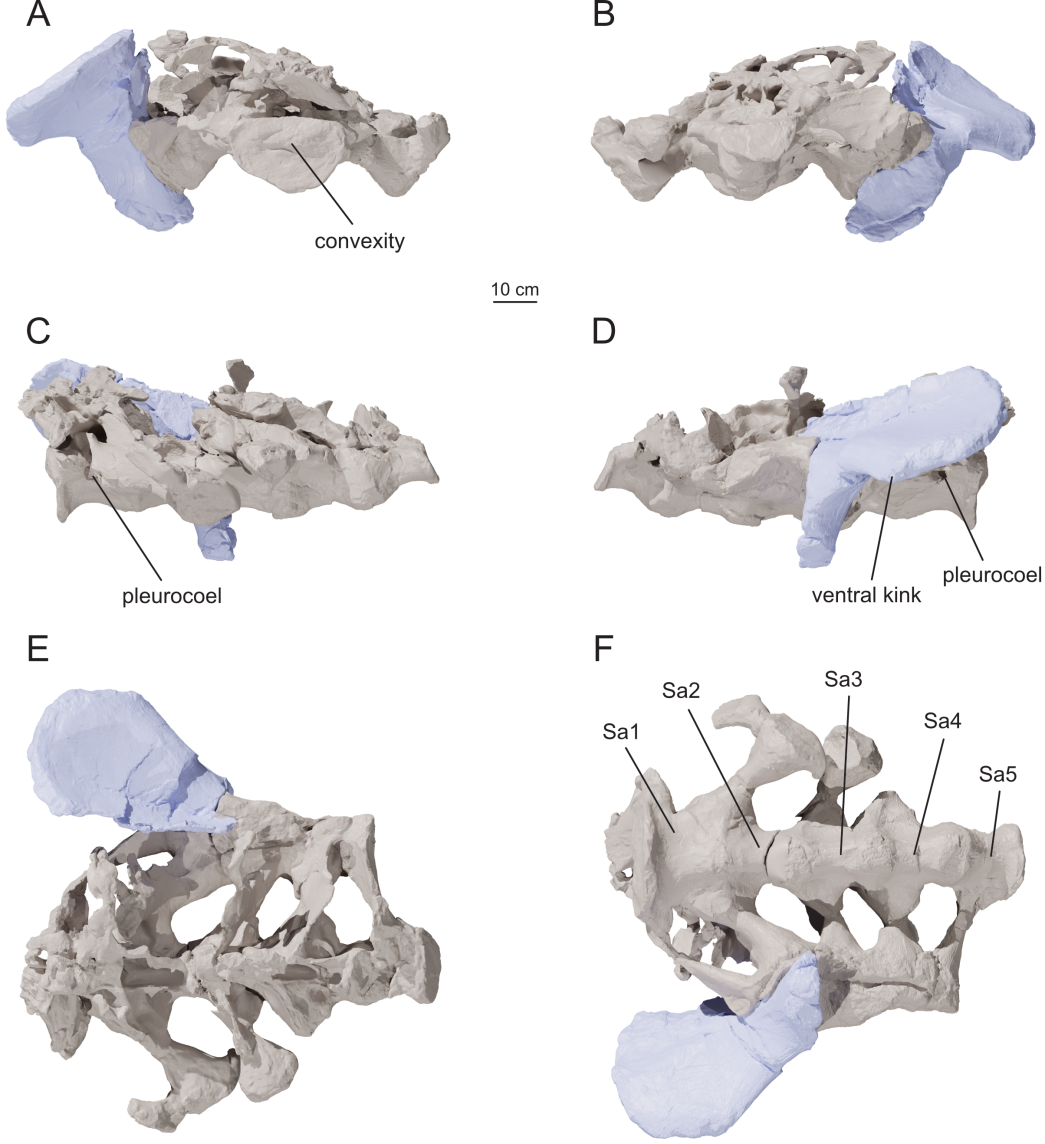
Articulated sacrum and ilium (MPEF-PV 1730/13) of *Bicharracosaurus dionidei*. In (A) anterior; (B) posterior; (C) left lateral; (D) right lateral; (E) dorsal and (F) ventral views. Images were taken from 3D scans in orthographic projection (3D model credit: Ethel Dening; Image credit: Victor Beccari). ‘Sa’ stands for sacral vertebra.

The sacrum (MPEF-PV 1730/13a-c; Table 3; Fig. 18) is fragmentary and only the centra and ribs are preserved. It is fused to the partial right ilium. During preparation the sacrum was prepared in three pieces, the first two sacral vertebrae, the last three sacrals and the ilium.

The sacrum is composed of five fused vertebrae (Sa1-Sa5), as in most non-titanosaurian neosauropods (Salgado, Coria & Calvo, 1997). All five centra that compose the sacrum are fused together. The sacrum is narrow (the mediolateral width across the centra and ribs at midlenght is 3.7 times the anteroposterior average length of the centra) as in some basal macronarians, such as *Camarasaurus*, *Galvesaurus*, *Europasaurus,* and *Vouivria* (Poropat et al., 2016; Mannion, Allain & Moine, 2017). Only the anterior articulation of the first sacral and the posterior one of the fifth sacral are visible. The anterior articulation of the first sacral centrum is very slightly convex, with the small convexity being wider than high and placed dorsally, matching the shape of the posterior articulation of the last dorsal (Fig. 18A). The posterior articulation of the fifth sacral is flat. The articular facets of the sacral centra are much wider than they are high (Table 3). The articulations are widest dorsally and have a straight dorsal margin, so that the shape is not oval but boat-like (Figs. 18A–18B). This shape is considered an autapomorphy of *Bicharracosaurus*. The posterior articulation of the fifth sacral is notably smaller in both width and height than the anterior articulation of the first sacral and then that of the first caudal vertebra (Tables 3–4). The length of the centra remains approximately constant along the sequence. The ventral surfaces are mediolaterally convex and in lateral view anteroposteriorly concave, with a rounded swelling at the fusion point between two subsequent vertebrae.

**Table 3:** Measurements (mm) of the sacral vertebrae of *Bicharracosaurus dionidei*. Centrum height and width was measured on the anterior articular surface for Sa1 and on the posterior articular surface for Sa5.

	**Sa1**	**Sa2**	**Sa3**	**Sa4**	**Sa5**
**Centrum height**	170	–	–	–	90
**Centrum width**	260	–	–	–	160
**Centrum length**	165	145	180	195	155

Pleurocoels, normally present in Titanosauriformes (Mannion et al., 2013) and in at least some sacrals of some basal macronarians, such as *Camarasaurus* (BYU 9041: A. Reutter, pers. obs., 2024), *Lourinhasaurus alenquerensis* (Mocho, Royo-Torres & Ortega, 2014: fig. 11), *Tehuelchesaurus* (Carballido et al., 2011b: fig. 13) and *Tastavinsaurus* (Canudo, Royo-Torres & Cuenca-Bescós, 2008) are also present in the first sacral of *Bicharracosaurus* (Figs. 18C–18D). The pleurocoel is notably smaller than the pleurocoels in the dorsal vertebrae, and also relatively smaller than the pleurocoel in the first sacral vertebra of *Tehuelchesaurus* (Carballido et al., 2011b: fig. 13), and is placed centrally on the centrum. A camellate internal structure, like in some somphospondylans (Mannion et al., 2013), is absent in *Bicharracosaurus*, as can be seen from the breakpoint between the second and third sacral. Whether the pleurocoel in the first sacral ramifies within the centrum or if it is a simple blind pocket remains unclear. The sacral ribs are fused to the centra and fuse laterally with each other, forming a sacricostal yoke, as in most eusauropods (Wilson & Sereno, 1998). Each vertebra, even the first and last one, have fused ribs that emanate from the anterior margin of the centrum. The sacral ribs are especially massive in Sa2-4, where they form part of the acetabulum. In contrast to most sauropods, including *Tehuelchesaurus,* the second sacral rib has a dual contribution from Sa1 (Fig. 18F) as in brachiosaurids (Mannion, Allain & Moine, 2017). Interestingly, this dual contribution might have also been present in a hip that could have been potentially referred to *Janenschia* (Fraas, 1908: fig. 10) although the material was never collected and did not preserve any overlapping material to allow a certain referral to the taxon (see Mannion et al., 2019b: p. 788).

**Table 4:** Measurements (mm) of the caudal vertebrae of *Bicharracosaurus dionidei*. Centrum height and width was measured on the anterior articular surface for MPEF-PV 1730/14 and on the posterior articular surface all other caudals. (I) MPEF-PV 1730/14; (II) MPEF-PV 1730/15; (III) MPEF-PV 1730/16; (IV) MPEF-PV 1730/17; (V) MPEF-PV 1730/18; (VI) MPEF-PV 1730/19; (VII) MPEF-PV 1730/20; (VIII) MPEF-PV 1730/21; (IX) MPEF-PV 1730/22.

	**I**	**II**	**III**	**IV**	**V**	**VI**	**VII**	**VIII**	**IX**
**Centrum height**	165^*^	125	105	110	95	80	65^*^	60	60^*^
**Centrum width**	195	105	100	100^*^	100	65^*^	70^*^	55	55^*^
**Centrum length**	120^*^	100	110	105	105	110	100^*^	100	95^*^
**Neural arch height**	–	–	40	40	–	–	–	–	–
**Neural spine height**	–	–	25^*^	–	–	–	–	–	–
**Neural spine width**	–	–	15	–	–	–	–	–	–
**Neural spine length**	–	–	55	–	–	–	–	–	–

**Notes.**

An asterisk (*) denotes a measurement that is based on an incomplete or deformed element.

**Figure 19:**
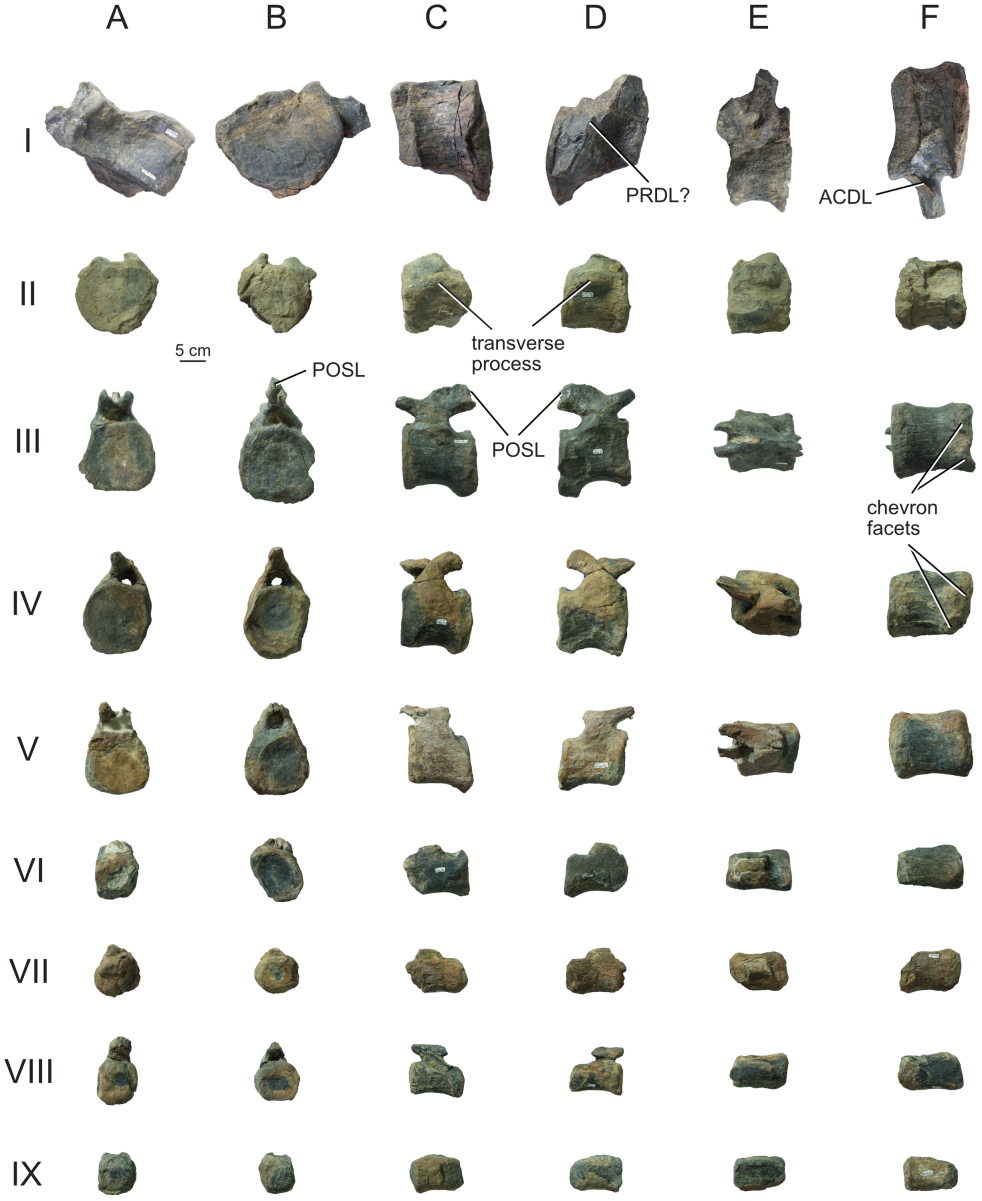
Caudal vertebrae (MPEF-PV 1730/14-22) of *Bicharracosaurus dionidei*. In (A) anterior; (B) posterior; (C) left lateral; (D) right lateral; (E) dorsal and (F) ventral views. (I) MPEF-PV 1730/14;(II) MPEF-PV 1730/15; (III) MPEF-PV 1730/16; (IV) MPEF-PV 1730/17; (V) MPEF-PV 1730/18; (VI) MPEF-PV 1730/19; (VII) MPEF-PV 1730/20; (VIII) MPEF-PV 1730/21 and (IX) MPEF-PV 1730/22. Abbreviations: ACDL, anterior centrodiapophyseal lamina; POSL, postspinal lamina; PRDL, prezygodiapophyseal lamina.

#### Caudal vertebrae

A total of nine caudal vertebrae (MPEF-PV 1730/14-22; Table 4; Fig. 19) were collected at the site of *Bicharracosaurus*. None of these were found in articulation. Anterior caudals are identified by the presence of a transverse process. Posterior caudals are distinguishable from middle caudals by having an aEI closer to 1,5 or higher. The most anteriorly preserved caudal vertebra (MPEF-PV 1730/14) has a transverse process that is dorsoventrally higher than anteroposteriorly long, its dorsal surface is broken, suggesting that the rib would have reached high on the neural arch, chevron facets are absent and a prominent sheet-like ACDL is present, all characters that are consistent with the morphology of anteriormost caudal vertebrae. The rest of the caudals represent one posterior proximal vertebra (MPEF-PV 1730/15), three middle (MPEF-PV 1730/16-18) and four distal vertebrae (MPEF-PV 1730/19-22). Several caudals do not preserve the neural arch and only MPEF-PV 1730/16 preserves part of the neural spine.

In the anteriormost caudal vertebra (MPEF-PV 1730/14) the anterior face of the centrum is concave (Fig. 19 IA), as is the common condition among sauropods (Salgado, Coria & Calvo, 1997). The posterior articular surface is flat, contrasting with the procoelous condition of some mamenchisaurs (*e.g.*, Ouyang & Ye, 2002: fig. 30; Sekiya, 2011: fig. 21), titanosaurs (*e.g.*, Martínez et al., 2004: fig. 6; PVL 4017: A. Reutter, pers. obs., 2023) and flagellicaudatans (*e.g.*, Gilmore, 1936: plate 16; Janensch, 1929: plate 3). All other preserved caudals are amphicoelous unlike most Somphospondyli (Mannion et al., 2013). The anterior articular surface is almost equally wide as it is high throughout the caudal series (Table 4). The anteroposterior length of the centra does not increase along the anterior sequence, unlike in diplodocids and rebbachisaurids (Mannion et al., 2019b). In the middle caudals of *Bicharracosaurus* the aEI is lower than that of titanosaurs, diplodocids and rebbachisaurids (Mannion et al., 2013; Tschopp, Mateus & Benson, 2015). The ventral surface is flat to convex (Fig. 19F), unlike the mediolaterally narrow ventral hollow of some titanosaurs (*e.g.*, *Andesaurus delgadoi;* Mannion & Calvo, 2011: figs. 4F, 5B; *Euhelopus*; Wilson & Upchurch, 2009: fig. 7D) or the wide hollow of some diplodocids (McIntosh, 1990a). There are no vascular foramina piercing the lateral or ventral surface of anterior and middle caudals, as seen in several titanosaurs and some other sauropods (see Mannion & Calvo, 2011). The typical pleurocoels on anterior caudals of diplodocids (Hatcher, 1901; Fraas, 1908; Remes, 2006; Lovelace, Hartman & Wahl, 2007; Gallina et al., 2014) are absent in *Bicharracosaurus*. The internal morphology of the first caudal centrum is exposed on the anterior and dorsal aspect. The solid structure is shared with most sauropods, with the exception of some lithostrotians (Mannion et al., 2013). Chevron facets are unequivocally present in MPEF-PV 1730/16 and 17 on the posterior margin of the centrum (Fig. 19 III-IVF).

Only the right transverse process of the first caudal is preserved, although it is broken distally and dorsally. The transverse process is situated on the laterodorsal side of the centrum and reaches the neural arch dorsally, unlike the more dorsally placed transverse processes of rebbachisaurids (Mannion et al., 2019b). Medially it is higher than wide. It is supported from below by an undivided, prominent and sheet-like ACDL (Fig. 19 IF). This morphology is rather uncommon within non-Somphospondyli Macronaria, but is also present in *Giraffatitan* (Janensch, 1950: p. 62) and *Vouivria* (Mannion, Allain & Moine, 2017: fig. 15A). Below the transverse process and delimited anteriorly by the ACDL there is a rounded fossa. A PRDL seems to be present (Fig. 19 ID), but the region is not well preserved, and it is not possible to determine if this lamina reached the prezygapophysis. In anterior or posterior view the ventral margin of the transverse process is directed laterally, as is the dorsal margin, giving it a triangular shape, unlike the wing-like transverse processes of diplodocoids (McIntosh, 1990a; McIntosh, 1990b: fig. 16.7). In dorsal view the transverse process is directed laterally, as in most non-titanosauriform sauropods (Mannion & Calvo, 2011). The transverse process is placed dorsally on the centrum and is reduced to an anteroposteriorly long ridge in the posterior proximal caudal vertebra (Fig. 19 IIC–D) and is completely absent in posterior middle and distal caudals.

Only the middle caudal vertebrae preserve the neural arch. Here the neural arch is positioned on the anterior half of the centrum (Figs. 19C–19D) as in Titanosauriformes (Calvo & Salgado, 1995; Salgado, Coria & Calvo, 1997) and some basal macronarians, such as *Galvesaurus* (Pérez-Pueyo et al., 2019: fig. 4) and *Europasaurus* (Carballido & Sander, 2014). The neural arch is low in middle caudals, as in most Sauropods, whereas occasionally the neural arches of middle caudals remain high in a few taxa, such as *Tastavinsaurus* (Canudo, Royo-Torres & Cuenca-Bescós, 2008: fig. 8A), *Malarguesaurus* (González Riga, Previtera & Pirrone, 2009: fig. 6), *Haplocanthosaurus priscus* (Hatcher, 1903: plate 3) and some rebbachisaurids (Carballido et al., 2012: p. 644). A shelf on the lateral side of the neural arch between the pre- and postzygapophyses, as seen in *Lusotitan* (Mannion et al., 2013: fig. 7), is absent. The prezygapophyses project anteriorly and slightly dorsally, as in *Mamenchisaurus youngi* (Ouyang & Ye, 2002: fig. 32), *Wamweracaudia* (Mannion et al., 2019b: fig. 27; MB.R.2091.1-30: A. Reutter, pers. obs., 2023) and some titanosaurs (Mannion & Calvo, 2011: p. 165). They extend only slightly anterior to the anterior margin of the centrum, unlike the extreme anterior extension of titanosaurs (González Riga, 2003). In MPEF-PV 1730/16, the postzygapophyses and part of the neural spine are preserved. The neural spine is much longer anteroposteriorly than it is mediolaterally wide and a POSL can be identified (Fig. 19 III).

#### Ilium

The ilium is fragmentary (MPEF-PV 1730/13c) and only the preacetabular process and part of the pubic peduncle are preserved (Fig. 18). In contrast to several titanosaurs (Wilson & Upchurch, 2009; Poropat et al., 2015), the ilium of *Bicharracosaurus* is not pneumatized. The preacetabular process it thick whereas towards the dorsal blade it becomes thin. The preacetabular process has a rounded outline, as in most Titanosauriformes (Wilson & Sereno, 1998). Similar to *Europasaurus* (Carballido et al., 2020: fig. 8), *Giraffatitan* (Janensch, 1961: suppl. E) and some lithostrotians (D’Emic, 2012) a ventral kink or bulge at the anteroventral margin of the preacetabular process is also present in *Bicharracosaurus* (Fig. 18D). The preacetabular process is directed slightly laterally as in most neosauropods, but not to the extent of some titanosaurs, such as *Epachtosaurus sciuttoi* (Martínez et al., 2004: figs. 2, 11), *Saltasaurus* (Powell, 1992: fig. 17), *Opisthocoelicaudia skarzynskii* (Borsuk-Bialynicka, 1977: p. 33) and *Diamantinasaurus* (Poropat et al., 2015: p. 1015). In these taxa the preacetabular process forms a semi-horizontal plate, which is also present in *Bicharracosaurus* (Fig. 18). However, in the latter taxon, the horizontalization of the ilium occurs independently from the lateral projection of the preacetabular process, and the lateral surface of the preacetabular process faces almost entirely more dorsally than laterally. Until now, this condition has not been described outside Titanosauria. The articular surface for the pubis is not well preserved, but distally the mediolateral width to anteroposterior length ratio of the pubic peduncle is 0.4, similar to the ratio in the basal macronarians *Europasaurus* (Carballido et al., 2020), *Tastavinsaurus* (Canudo, Royo-Torres & Cuenca-Bescós, 2008: fig. 10 B), *Giraffatitan* (Janensch, 1961: p. 200) and *Euhelopus* (Wilson & Upchurch, 2009: p. 223). The angle between the pubic peduncle and the long axis of the ilium or of the long axis of the sacrum is greater than 90° (Fig. 18D) as in several macronarians, including the early-branching *Europasaurus* (Carballido et al., 2020: fig. 8) and *Tastavinsaurus* (Canudo, Royo-Torres & Cuenca-Bescós, 2008: fig. 10) and brachiosaurids (Janensch, 1961; Mannion, Allain & Moine, 2017: fig. 29).

**Table 5:** Matrices used in this study and tree search results. The number of taxa and characters takes into account only active taxa or characters. ‘#’ stands for number.

**Matrix**	**# Characters**	**# Taxa**	**Search method**	**Random seed**	**MPTs length**	**# MPTs**
**RE23**	403	87	EW	Default	1450	Overflow
			EIW (*k* = 3)	Default	124,39258	104
			EIW (*k* = 8)	Default	71,72773	4
			EIW (*k* = 13)	Default	51,52277	18
**UP21**	556	119	EW	Default	2751	Overflow
			EIW (*k* = 3)	Default	204,08253	24948
			EIW (*k* = 8)	8	127,21644	27
			EIW (*k* = 13)	Default	95,69024	8424
**RE23 STS**	364	65	EIW (*k* = 7)	Default	63,96534	4
**UP21 STS**	548	65	EIW (*k* = 7)	Default	115,14239	90

### Phylogenetic analysis

#### The position of Bicharracosaurus, MPEF-PV 1324, and Tehuelchesaurus

**Figure 20:**
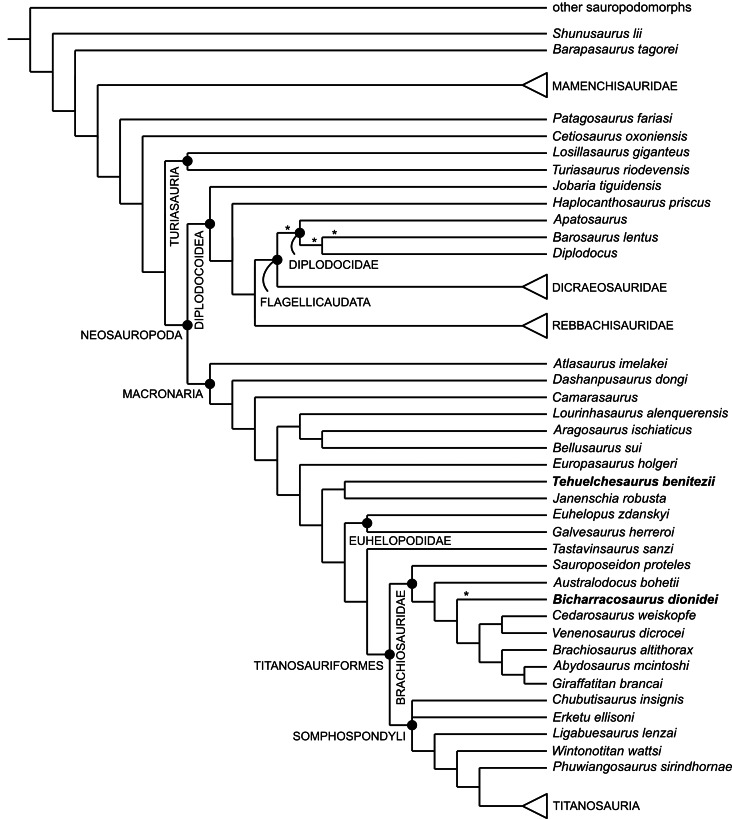
Reduced consensus tree of the extended implied weights (*k* = 8) analysis using the Ren et al. (2023) matrix. *A posteriori* pruning of MPEF-PV 1324. The asterisk (*) indicates its alternative positions. See text for details of phylogenetic analysis.

Table 5 summarizes our tree search results. Because MPEF-PV 1324 is alternatively placed with the *Bicharracosaurus* clade and Diplodocidae, the strict consensus trees of the EW and the EIW analyses of the RE23 matrix show a polytomy at the base of Neosauropoda (Article S1: figs. 1, 3, 5, 7), whereby the base of Titanosauria is also poorly resolved in the EW analysis. In every analysis *Bicharracosaurus*, MPEF-PV 1324, and *Tehuelchesaurus* are found within this polytomy. In two analyses, EIW3 and EIW8, *Tehuelchesaurus* is found as a sister taxon to *Janenschia* within the polytomy. The *a posteriori* pruning of 13 taxa in the EW analysis resolves the base of Neosauropoda and Titanosauria (Article S1: fig. 2). The *a posteriori* pruning of MPEF-PV 1324 resolves the base of Neosauropoda in the EIW analyses (Fig. 20; Article S1: figs. 4, 6, 8). While *Bicharracosaurus* is recovered as a basal macronarian, in a clade with *Tehuelchesaurus* and *Janenschia*, in the EW and EIW13 analyses, it is recovered within Brachiosauridae, intercalated between *Australodocus* and the clade containing *Cedarosaurus*, *Venenosaurus*, *Abydosaurus*, *Giraffatitan*, and *Brachiosaurus*, in the EIW3 and EIW8 analyses. In all analyses, the specimen MPEF-PV 1324 is recovered close to *Bicharracosaurus* and within Diplodocidae. In the EW and EIW13 analysis, MPEF-PV 1324 is recovered as a sister taxon to *Bicharracosaurus*, to *Janenschia*, to the clade containing *Bicharracosaurus + Tehuelchesaurus + Janenschia*, or in different positions within Diplodocidae. In the EIW3 and EIW8 analyses, MPEF-PV 1324 is recovered as a sister taxon to *Bicharracosaurus*, within Brachiosauridae, or in different positions within Diplodocidae. In every analysis, *Tehuelchesaurus* and *Janenschia* are found as sister taxa within basal Macronaria, with this clade sometimes (EW, EIW13) including *Bicharracosaurus*.

**Figure 21:**
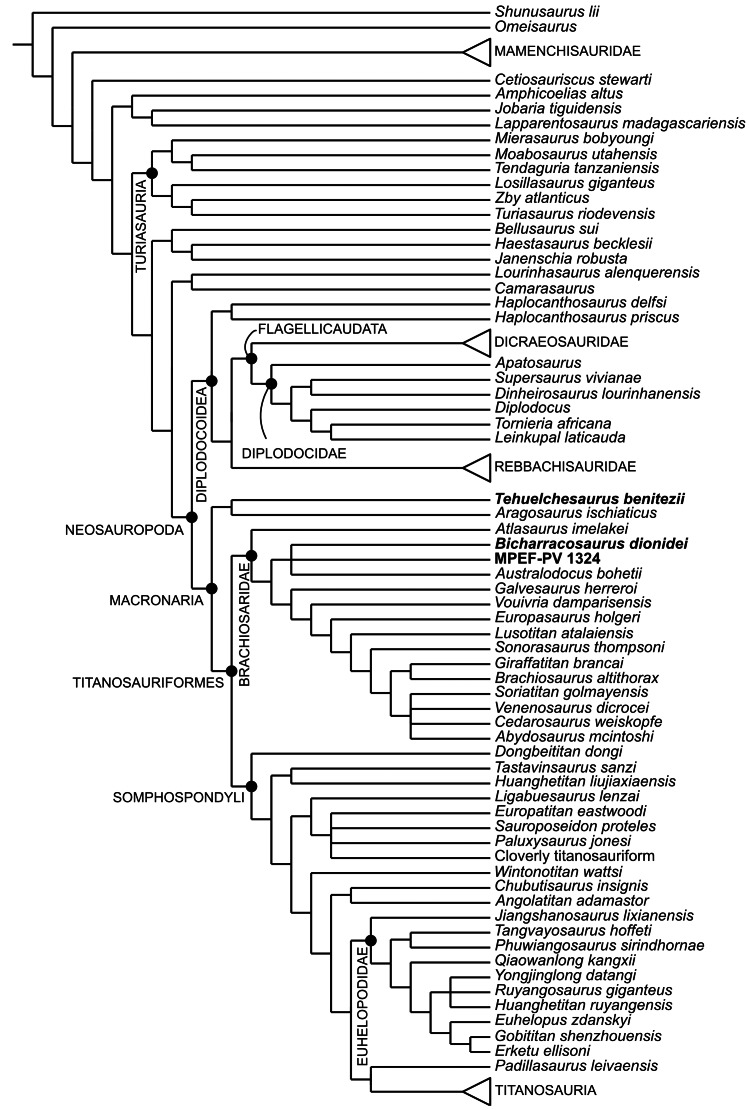
Strict consensus tree of the extended implied weights (*k* = 8) analysis using the Upchurch et al. (2021) matrix. See text for details of phylogenetic analysis.

In the UP21 EW analysis, both *Bicharracosaurus* and MPEF-PV 1324 are recovered in polytomy with *Lusotitan* as closest relatives to Titanosauriformes, while *Tehuelchesaurus* forms a clade with *Camarasaurus*, *Haestasaurus*, and *Janenschia*, also as basal macronarians (Article S1: figs. 9–10). *Bicharracosaurus* and MPEF-PV 1324 are recovered in a polytomy together with *Australodocus* within Brachiosauridae, intercalated between *Atlasaurus* and *Galvesaurus* in all EIW (Fig. 21; Article S1: figs. 10–13). *Tehuelchesaurus* is recovered as a sister taxon to *Aragosaurus* and as the closest relative to Titanosauriformes in the EIW3 and EIW8 analyses (Fig. 21; Article S1: figs. 11–15), but outside Neosauropoda in the EIW13 analysis. Here, *Tehuelchesaurus* is the closest relative to the clade *Bellusaurus* + (*Haestasaurus* + *Janenschia*).

In general, the results of constraint analyses and Templeton Tests indicate that the alternative topologies cannot be discarded with significant confidence. Constraining *Bicharracosaurus* within Brachiosauridae, with MPEF-PV 1324, *Tehuelchesaurus*, *Janenschia*, *Cedarosaurus*, *Sauroposeidon*, and *Australodocus* as floaters, in the EW RE23 analysis, requires four extra steps. The number of characters favouring the MPT is 39 compared to 40, which fit better the forced topology. Likewise, constraining *Bicharracosaurus* within Diplodocidae, with MPEF-PV 1324, *Tehuelchesaurus*, and *Janenschia* as floaters, requires 10 extra steps. The number of characters favouring the MPT is 44 compared to 38, which fit the forced topology better. Constraining *Bicharracosaurus* within Brachiosauridae in the EW UP21 analysis, with MPEF-PV 1324, *Lusotitan*, *Galvesaurus*, *Atlasaurus*, *Australodocus*, *Vouivria*, and *Europasaurus* as floaters, requires three extra steps. The number of characters that favour the MPT is 20 in comparison to 17, which better fit the suboptimal topology. Finally, the constraint analysis with *Bicharracosaurus* within Diplodocidae, and MPEF-PV 1324, *Lusotitan*, and *Australodocus* as floaters requires 20 extra steps. The number of characters favouring the MPT is 86 compared to 73, which fit better the forced topology.

#### The position of other putative macronarian taxa

As with *Bicharracosaurus,* other macronarian taxa are also found in varying positions within the clade, depending on the dataset and/or the analytical method. Such taxa include *Galvesaurus*, *Tastavinsaurus*, *Euhelopus,* and *Australodocus*. Like *Tehuelchesaurus*, several other putative macronarian taxa are found within Macronaria or outside Neosauropoda in the analyses performed here. These taxa include *Tendaguria* and *Lourinhasaurus*, and closely related taxa to *Tehuelchesaurus*, like *Camarasaurus, Janenschia*, and *Bellusaurus*.

*Galvesaurus*, *Tastavinsaurus*, and *Euhelopus* are recovered as early members of Macronaria in all RE23 analyses (Fig. 20; Article S1: figs. 1–8). Only in the EW UP21, is *Galvesaurus* recovered as a basal macronarian (Article S1: figs. 9–10), whereas in the EIW UP21 analyses it is recovered within Brachiosauridae intercalated between the clade (*Bicharracosaurus* + *Australodocus* + MPEF-PV 1324) and *Vouivria* (Fig. 21; Article S1: figs. 11–15). Alternatively, *Galvesaurus* can also be recovered as Somphospondyli in the EIW13 UP21 analysis. *Tastavinsaurus* is recovered as the closest relative to Titanosauriformes in all RE23 analyses, but within Somphospondyli in all the UP21 analyses. *Euhelopus* is always recovered within Somphospondyli in the UP21 analyses, sometimes inside Titanosauria (EIW3 and EIW13). *Australodocus* is recovered within Somphospondyli in the EW RE23 and UP21 analyses and the EIW13 RE23 analysis.

*Tendaguria* is found within Somphospondyli in every RE23 analysis, either inside Titanosauria or close to it. However, it is recovered outside Neosauropoda in every UP21 analysis, together with *Moabosaurus* and *Mierasaurus*, either inside Turiasauria or very close to it. *Lourinhasaurus* and *Camarasaurus* are recovered as early-branching macronarians in all the RE23 and the EW UP21 analyses, but as sister taxa and as closest relatives of Neosauropoda in the UP21 EIW analyses. In all the RE23 analyses and the EW UP21 analysis, *Janenschia* is found in a position together with *Tehuelchesaurus* within basal Macronaria. In the EIW8 and 13 UP21 analyses, *Janenschia* is recovered in a clade with *Bellusaurus* and *Haestasaurus* outside Neosauropoda in a slightly more derived position than Turiasauria. Additionally, in the EIW3 UP21 analyses, *Janenschia* is in a polytomy with *Haplocanthosaurus* and *Haestasaurus* within Diplodocoidea. While *Bellusaurus* is recovered within Macronaria, as a sister taxon to *Aragosaurus*, in all the RE23 analyses, it is found outside Neosauropoda, intercalated between Turiasauria or Turiasauria-like taxa (*Mierasaurus*, *Moabosaurus*, and *Tendaguria*), and the *Camarasaurus* + *Lourinhasaurus* clade, in all the UP21 ones.

### STS analyses

In the RE23 dataset, non-overlapping taxa were mostly non-eusauropod sauropodomorphs. Likewise, most characters that turned uninformative after reducing the taxonomic scope were related to basal Sauropodomorpha morphologies. In contrast, most unshared taxa in the UP21 dataset were Titanosauriformes and only a few of these characters became uninformative in the UP21 STS analyses. In total the STS datasets contain 65 taxa scored for 364 and 548 characters in the RE23 and UP21 STS datasets respectively (see Table 5).

**Figure 22:**
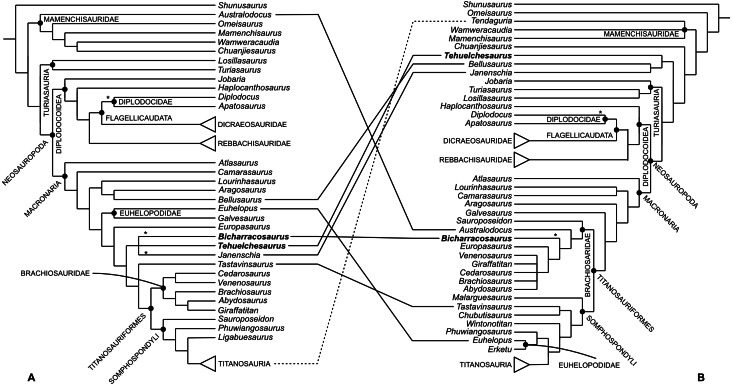
Results of the shared taxonomic scope analyses. (A) RE23 STS reduced consensus tree by *a posteriori* pruning of MPEF-PV 1324 and (B) UP21 STS strict consensus tree by *a posteriori* pruning of MPEF-PV 1324. The asterisk (*) indicates the alternative positions of MPEF-PV 1324. Full lines indicate incongruent positions position; the dotted line indicates the incongruent position of *Tendaguria* within Titanosauria in the RE23 STS reduced consensus tree. See text for details of shared taxonomic scope phylogenetic analysis.

In all analyses, the strict consensus trees are not well resolved at the base of Neosauropoda (Article S1: figs. 16, 18). The *a posteriori* pruning of MPEF-PV 1324 improves the resolution at this node (Fig. 22; Article S1: figs. 17, 19). The general topology of the RE23 STS reduced consensus tree is very similar to that of RE23 EW and EIW13. The topology of the UP21 STS reduced consensus tree is somewhat different from the UP21 analyses, with some putative macronarian taxa now being recovered in a similar position to that in the RE23 STS analysis.

*Bicharracosaurus* is recovered within Macronaria in both analyses, either outside Titanosauriformes, together with *Tehuelchesaurus* and *Janenschia*, in the RE23 STS or within it, as a brachiosaurid, in the UP21 STS analysis, intercalated between *Australodocus* and *Europasaurus*. The alternative positions of MPEF-PV 1324 are very similar between the two datasets, either as a sister taxon to *Bicharracosaurus* (or additionally to *Janenschia* in the RE23 STS analysis) or to diplodocids. *Tehuelchesaurus* is recovered with *Bicharracosaurus* and *Janenschia* in the RE23 STS analysis but outside Neosauropoda, in a clade with *Bellusaurus* and *Janenschia*, as sister clade to Turiasauria + Neosauropoda.

*Galvesaurus* is found as a basal macronarian in both analyses. *Tastavinsaurus* and *Euhelopus* are recovered within basal macronarians in the RE23 STS but inside Somphospondyli in the UP21 STS analysis. The brachiosaurid phylogenetic relationship of *Australodocus* is recovered only in the UP21 STS analyses, while in the RE23 STS analysis it is recovered within Mamenchisauridae. *Tendaguria* is still within Titanosauria in the RE23 STS dataset but within Mamenchisauridae in the UP21 STS dataset. In both analyses, *Camarasaurus* and *Lourinhasaurus* are recovered as basal macronarians. In both matrices, *Janenschia* is recovered in a clade with *Tehuelchesaurus*, as basal macronarians in the RE23 STS analysis or as a sister clade to Turiasauria + Neosauropoda in the UP21 STS analysis. Consistent with the RE23 and UP21 analyses *Bellusaurus* is recovered as a basal macronarian or non-neosauropod eusauropod respectively.

## Discussion

### The systematic affinities of *Bicharracosaurus*

*Bicharracosaurus* was recovered as a macronarian in all analyses performed here, a position supported by mediolaterally wider than dorsoventrally high presacral centra, neural spines of posterior cervicals with laterodorsal rugose ridge, presence of PRSL in dorsal neural spines, pleurocoel with acute posterior margin in anterior dorsals, ventrally divided PCDL in posterior dorsal vertebrae, plank-like anterior dorsal ribs, presence of pleurocoels in sacral vertebrae, anteriorly placed neural arch in mid-caudals, aEI of posterior caudal less than 1.7, a semi-circular preacetabular process, perpendicular pubic peduncle of the ilium, and pubic peduncle at least two times wider lateromedially than long anteroposteriorly.

Within Macronaria, *Bicharracosaurus* was recovered in a basal position outside Brachiosauridae in the EW and EIW13 RE23 and the EW UP21 analyses. While triangular aliform processes are common among macronarians, including brachiosaurids (Wilson & Sereno, 1998), these are absent in the middle and posterior dorsal neural spines of *Bicharracosaurus*. Unlike brachiosaurids, the anterior caudal centra of *Bicharracosaurus* do not bear pleurocoels on their lateral surfaces (D’Emic, 2012). Such pleurocoels are also found in diplodocids and a few titanosaurs (Calvo & Salgado, 1995; Mannion et al., 2013). A ‘shoulder’ between the prezygapophysis and the postzygapophysis in anterior to middle caudals, as in *Giraffatitan*, *Lusotitan*, *Sonorasaurus thompsoni*, and some titanosaurs (D’Emic, Foreman & Jud, 2016; Mannion et al., 2019b), is absent in *Bicharracosaurus*.

Furthermore, *Bicharracosaurus* was recovered within Brachiosauridae in the EIW3 and EIW8 RE23 and all the EIW UP21 analyses. Unlike most macronarians, the middle cervical prezygapophyses of *Bicharracosaurus* bear a lateral fossa, as do those of *Brachiosaurus*, *Giraffatitan*, and *Europasaurus*. Outside Macronaria, such fossae have been identified in diplodocoids and *Moabosaurus* (Harris, 2006a; Tschopp & Mateus, 2013; Mannion et al., 2019b). Whereas the surface of the SDF usually is smooth in the middle and posterior cervical vertebrae of sauropods, in *Vouivria* (Mannion, Allain & Moine, 2017: fig. 10) and *Giraffatitan* (Janensch, 1950: fig. 29, 34, 37, 43), both unequivocal brachiosaurid taxa, the SDFs are pierced by several coels. Very few other taxa share this unique morphology, including the putative brachiosaurids *Europasaurus* (Carballido & Sander, 2014: figs. 5, 7) and *Australodocus* (Remes, 2007: fig. 2), and the mamenchisaurid *Omeisaurus puxiani* (Tan et al., 2021: fig. 3). Slightly opisthocoelous posterior dorsal centra, is common among diplodocoids and turiasaurs (Royo-Torres et al., 2017b; Mannion, Tschopp & Whitlock, 2021), while macronarians tend to retain strong opisthocoely in posterior dorsal centra (Calvo & Salgado, 1995; Upchurch, Barrett & Dodson, 2004; Carballido et al., 2011b). However rare for macronarians, slightly opisthocoelous posterior dorsal centra have been identified in brachiosaurid-like taxa (*Lusotitan* (Mannion et al., 2013: fig. 2; Mocho, Royo-Torres & Ortega, 2017), *Vouivria* (Mannion, Allain & Moine, 2017: fig. 13A), in the last dorsal vertebra of *Brachiosaurus* (Taylor, 2009: fig. 1N), and possibly in *Soriatitan* (Royo-Torres et al., 2017a: fig. 3A), and in *Atlasaurus* (Monbaron, Russell & Taquet, 1999: fig. 1I)). The second sacral rib, with the contribution of the first sacral centrum, has only been identified in *Vouivria*, *Brachiosaurus*, and *Giraffatitan* (see Mannion, Allain & Moine, 2017).

From the latter, it is clear that these character states are uncommon among sauropods or within Macronaria, and their presence in *Bicharracosaurus* strongly support its position within Brachiosauridae. Several uncommon character states supporting a position within Brachiosauridae explain why this position was only recovered in EIW analyses but never in EW analyses. In other words, by penalizing homoplastic characters in EIW, less homoplastic characters gain in relative weight. When considering that EIW analyses produce more accurate results than EW (Goloboff et al., 2008; Goloboff, Torres & Arias, 2018; Ezcurra, 2024) and considering that constraint analyses and the Templeton test do not reject this topology for any dataset, we consider it possible that *Bicharracosaurus* represents a brachiosaurid from the Cañadón Calcáreo Formation, supporting the presence of this clade in the Jurassic of South America.

The fossil record of Brachiosauridae spans from the early Late Jurassic (Mannion, Allain & Moine, 2017) to the early Late Cretaceous (D’Emic, Foreman & Jud, 2016). Putative representatives of this group have been found in Europe (Mannion et al., 2013; Mannion, Allain & Moine, 2017; Royo-Torres et al., 2017a; Carballido et al., 2020), North America (Riggs, 1901; Tidwell, Carpenter & Brooks, 1999; Tidwell, Carpenter & Meyer, 2001; Chure et al., 2010; D’Emic, Foreman & Jud, 2016), Africa (Janensch, 1914; Monbaron, Russell & Taquet, 1999) and possibly also South America (Rauhut, 2006; Carballido et al., 2015). While originally recovered as a brachiosaurid (Carballido et al., 2015), the current systematic affinities of *Padillasaurus*, from the Barremian Paja Formation of Colombia, are regarded as somphospondylan (Mannion, Allain & Moine, 2017; Mannion et al., 2019b). The fragmentary nature of the material described by Rauhut (2006), represented by two excavated specimens, does not allow for a certain referral to Brachiosauridae as it lacks synapomorphies of the group (Mannion et al., 2013). Nonetheless, the widespread distribution of Brachiosauridae during the Late Jurassic suggests that this clade spread during the Middle Jurassic and thus was probably present in South America (Mannion et al., 2019b).

It is worth pointing out that *Bicharracosaurus* was found a few hundred meters north of the material in Rauhut (2006) (see Fig. 2B). The overlapping material with *Bicharracosaurus* is limited and restricted to one of the described specimens (MPEF-PV 3099) mainly comprising some partially articulated caudal vertebrae with damaged neural arches. In both cases, the caudal vertebrae of *Bicharracosaurus* and MPEF-PV 3099 are amphicoelous unlike the procoelous condition of several titanosaurs and mamenchisaurids (Ouyang & Ye, 2002; Mannion et al., 2013; Mannion et al., 2019b), a ventral longitudinal hollow is absent unlike that of titanosaurs and diplodocoids (McIntosh, 1990b; Wilson & Upchurch, 2009; Mannion & Calvo, 2011) and the neural arch is placed anteriorly on the centrum as in titanosauriforms, *Galvesaurus*, and *Europasaurus* ((Mannion et al., 2013; Carballido & Sander, 2014; Pérez-Pueyo et al., 2019). MPEF-PV 3099 was recovered in an unstable position within Brachiosauridae in the phylogenetic analysis of Rauhut (2006). Given its close geographical and geological provenance, its morphological similarities with non-titanosaurian Titanosauriformes (including *Bicharracosaurus*), and the prospect for this material to represent a brachiosaurid, there is the possibility for MPEF-PV 3099 to represent another specimen of *Bicharracosaurus*. However, the incomplete nature of MPEF-PV 3099, and the lack of unique morphological features, does not allow further testing of the relationship between this specimen and *Bicharracosaurus*.

### The referral of MPEF-PV 1324 to *Bicharracosaurus*

The isolated and incomplete dorsal vertebrae assigned by (Rauhut, Carballido & Pol, 2015) to Diplodocidae (MPEF-PV 1324) were found in the same formation, approximately two kilometres southeast of the holotype of *Bicharracosaurus*, also in the higher part of the sequence (see Fig. 2B). The vertebrae are retrieved by the analyses close to *Bicharracosaurus* or within Diplodocidae in the RE23 dataset, or as a sister taxon to *Bicharracosaurus* in the UP21 dataset.

The character states that support a position next to *Bicharracosaurus*, in the RE23 and UP21 analyses, and alternatively a position within Diplodocidae, in the RE23 analyses, relate mainly to the shape of the pleurocoel and the interarticulation of the posterior dorsal centra. As described extensively above (see Results section), the dorsal margin of the pleurocoels is high on the centrum, approximately levelled with the ventral floor of the neural canal, and is also acutely angled in *Bicharracosaurus*, MPEF-PV 1324, *Apatosaurus*, *Diplodocus*, and *Barosaurus* (McIntosh, 2005; Rauhut, Carballido & Pol, 2015). However, both characters states are also present in *Moabosaurus* (Britt et al., 2017: fig. 3), *Haplocanthosaurus priscus* (Hatcher, 1903: plate 1; CM 572: A. Reutter, pers. obs., 2024), *H. delfsi* (McIntosh & Williams, 1988: fig. 8; CMNH 10380: A. Reutter, pers. obs., 2024), *Camarasaurus* (McIntosh et al., 1996: plate 5; SMA 0002, BYU 9047: A. Reutter, pers. obs., 2023, 2024) and *Europasaurus* (Carballido & Sander, 2014: figs. 17, 21–22; DFMMh/FV 712.1, 1195: A. Reutter, pers. obs., 2024). While the small triangular fossa, anteroventral to the pleurocoel, is absent in most sauropods, it is currently known to be present in MPEF-PV 1324 and *Diplodocus* (Rauhut, Carballido & Pol, 2015), as well as *Bicharracosaurus*. Slightly opisthocoelous posterior dorsal centra are common among diplodocoids, turiasaurs, and brachiosaurid-like taxa (Taylor, 2009; Mannion et al., 2013; Mannion, Allain & Moine, 2017; Royo-Torres et al., 2017a; Royo-Torres et al., 2017b; Mannion, Tschopp & Whitlock, 2021).

The only character state that supports a position as sister taxon to *Bicharracosaurus*, but not within Diplodocidae in the UP21 analyses, is that, as in *Bicharracosaurus*, the pleurocoels of MPEF-PV 1324 are not divided, unlike the divided pleurocoels of diplodocids (Salgado, Coria & Calvo, 1997; Mannion et al., 2012). However, the most evident difference between MPEF-PV 1324 and *Bicharracosaurus* is the relative sizes of the centra, with MPEF-PV 1324 having much bigger centra (see Table 2; Rauhut, Carballido & Pol, 2015: table 1), even though *Bicharracosaurus* is considered somatically mature by the histological evidence.

The evidence is scarce to infer the systematic affinities of MPEF-PV 1324 correctly. In any case, this specimen cannot be confidently referred to Diplodocidae, making the evidence for the presence of this family in the Cañadón Calcáreo Formation ambiguous, even though representatives of this family have been found in South America, in the Late Jurassic (Tithonian) Toqui Formation of Chile (Salgado et al., 2015), the Lower Cretaceous Bajada Colorada Formation of Argentina (Gallina et al., 2014), and the Lower Cretaceous Mulichinco Formation of Argentina (Gnaedinger et al., 2017). Given the stratigraphic and geographic provenance and the great morphological similarity, we tentatively refer the vertebrae (MPEF-PV 1324) to *Bicharracosaurus*, as this is the most parsimonious hypothesis thus far.

At this point, it should be noted that all the previously discussed character states shared between *Bicharracosaurus*, MPEF-PV 1324, and Diplodocidae also support a position of the former within Diplodocidae. However, as mentioned above, the only character state unique to these taxa is the small anteroventral fossa of the pleurocoel in middle to posterior dorsal vertebrae. Even though none of the phylogenetic analyses carried out here recover a close relationship between *Bicharracosaurus* and Diplodocidae, constraint analyses and a Templeton Test do not reject this hypothesis. This highlights the difficulties of solving the interrelationships at the origin of a group when derived morphologies are not fully developed.

### The generic separation from *Tehuelchesaurus*

In every RE23 analysis, *Tehuelchesaurus* and *Janenschia* are found as sister taxa within basal Macronaria, with this clade sometimes (EW, EIW13) including *Bicharracosaurus*. Such results are consistent with previous dataset versions (Carballido et al., 2011b; Carballido & Sander, 2014; Ren et al., 2021; Ren et al., 2023). In the UP21 EW analysis, *Tehuelchesaurus* forms a clade with *Camarasaurus*, *Haestasaurus*, and *Janenschia*, as basal macronarians. A similar clade, where *Tehuelchesaurus* is recovered as the closest relative to *Bellusaurus* + (*Haestasaurus* + *Janenschia*), is found outside Neosauropoda, intercalated between Turiasauria and Camarasaurus + Lourinhasaurus, in the EIW13 UP21 analysis. This finding is consistent with the EW result of Mannion et al. (2019b), with the only difference that the *Tehuelchesaurus*-clade is recovered within Turiasauria in the latter. In the EIW3 and EIW8 UP21 analyses, *Tehuelchesaurus* is recovered as a sister taxon to *Aragosaurus* and as a basal macronarian. Again, this is consistent with the EIW result of Mannion et al. (2019b). Of all the analyses performed here (EW, EIW3, EIW8, and EIW13 for two datasets), only two (EW and EIW13 RE23) support a close relationship between *Bicharracosaurus* and *Tehuelchesaurus*.

Even though shared character states between *Bicharracosaurus* and *Tehuelchesaurus* outnumber their differences, 72,3% (34 out of 47) of characters that are scored for both taxa in the RE23 matrix share the same character state, and 75,4% (or 46 out of 61) character states are shared in the UP21 matrix, only a few of these shared character states are diagnostic. These character states relate to the dorsal neural spines not having a triangular aliform process, the presence of it being the common condition among Macronaria (Wilson & Sereno, 1998; Mannion et al., 2013), and posterior dorsal neural spines being anteroposteriorly longer than mediolaterally, a reversal to the plesiomorphic condition of non-sauropod sauropodomorphs, shared with *Omeisaurus puxiani* (Tan et al., 2021: fig. 4), *Spinophorosaurus* Remes et al., 2009, *Jobaria* (Sereno et al., 1999: fig. 3C), and the macronarians *Europasaurus* (Carballido & Sander, 2014: figs. 21–22) and *Andesaurus* (Mannion & Calvo, 2011). In addition, *Bicharracosaurus* and *Tehuelchesaurus* share the presence of one accessory lamina of the parapophysis in middle and posterior dorsals.

Differences between *Bicharracosaurus* and *Tehuelchesaurus* include several diagnostic characters such as, the SPDL in anterior dorsals which are divided in *Bicharracosaurus* but simple in *Tehuelchesaurus*. A divided SPDL is uncommon among non-titanosaur sauropods (Salgado & Powell, 2010), and is only present in *Haplocanthosaurus priscus* (Hatcher, 1903: plate 1; CM 572: A. Reutter, pers. obs., 2024) and *Comahuesaurus windhauseni* (Carballido et al., 2012: figs. 2–3). The pleurocoels in the middle and posterior dorsal vertebrae of *Bicharracosaurus* have an anteroventral fossa (Fig. 13D), which has only been identified in MPEF-PV 1324 and *Diplodocus* thus far (Rauhut, Carballido & Pol, 2015). While the hyposphene of *Bicharracosaurus* is supported by paired CPOLs in the middle to posterior dorsal vertebrae, as in most sauropods (Janensch, 1929; Upchurch & Martin, 2002; Apesteguía, 2005; Taylor, 2009; Carballido et al., 2011a), in *Tehuelchesaurus* the hyposphene is supported from below only by a sTPOL, which was originally identified in *Diplodocus* and *Barosaurus* (Upchurch, 1995), but has since also been found in more basal forms, such as *Tazoudasaurus naimi* (Allain & Aquesbi, 2008: figs. 14C–D) and *Patagosaurus fariasi* (Holwerda, Rauhut & Pol, 2021: figs. 13–14D, 15E). Additionally, the hyposphene in middle and posterior dorsal vertebrae of B*icharracosaurus* has an accessory lamina that contacts the PCDL as in diplodocids (Bonaparte & Mateus, 1999; Mannion et al., 2012: fig. 3; Tschopp, Mateus & Benson, 2015). The posterior dorsal centra of *Bicharracosaurus* are slightly opisthocoelous, while they are strongly opisthocoelous in *Tehuelchesaurus* as in other macronarians (Calvo & Salgado, 1995; Upchurch, Barrett & Dodson, 2004; Carballido et al., 2011b). The parapophyses in the middle and posterior dorsal neural arches of *Bicharracosaurus* lie higher than the prezygapophyses, as in some rebbachisaurids (Wilson & Allain, 2015), the turiasaurs *Moabosaurus* (Britt et al., 2017: fig. 4) and *Mierasaurus* (Royo-Torres et al., 2017b: fig. 4), as well as in *Tastavinsaurus* (Canudo, Royo-Torres & Cuenca-Bescós, 2008: fig. 3C). As in some brachiosaurids (Mannion, Allain & Moine, 2017), the second sacral rib has a contribution from Sa1 in *Bicharracosaurus*, but this dual contribution is absent in *Tehuelchesaurus*.

Other Late Jurassic formations like the Morrison Formation, Lourinhã Formation, Villar del Arzobispo Formation, and the Tendaguru Formation each show a diverse array of sauropods, including several macronarian genera within the same formation (*e.g.*, *Lusotitan*, *Lourinhasaurus*, and *Oceanotitan dantasi* in the Lourinhã Formation (Mannion et al., 2013; Mocho, Royo-Torres & Ortega, 2014; Mocho, Royo-Torres & Ortega, 2017; Mocho, Royo-Torres & Ortega, 2019)). When taking into consideration that the Cañadón Calcáreo Formation extends over at least a few million years and that the coexistence of several macronarian species in one formation is common, it is expected that *Tehuelchesaurus* is not the only macronarian species in the formation. Based on the results of the phylogenetic analyses, the above list of morphological differences, and the possibility for another macronarian in the formation, we consider that there is strong evidence to support the new specimen as a different species and genus from *Tehuelchesaurus*.

### Incongruent phylogenetic results

Both matrices used here represent recent iterations of matrices widely used in eusauropod phylogeny, but as mentioned above, there are vast topological differences. The UP21 dataset includes a broader sample of eusauropod and characters compared to the RE23 dataset (see Table 5). Taxon choice has been discussed to have a major influence on phylogenetic results (Graybeal, 1998; Heath, Hedtke & Hillis, 2008) and is a first approach to investigate the root causes of phylogenetic incongruence (Sereno, 2009). In this study, the control of taxon choice has little effect on phylogenetic congruence (Fig. 22), so differences in topology can thus be explained by character choice, character state coding, and/or cell scoring.

For example, the UP21 dataset contains several putative brachiosaurid taxa not included in the RE23 matrix (*Vouivria*, *Lusotitan*, *Sonorasaurus*, and *Soriatitan*). However, excluding these taxa in the UP21 STS analysis does not affect the position of *Bicharracosaurus*, which is recovered as a basal macronarian and a brachiosaurid in the RE23 and UP21 STS analyses respectively. So, character choice, coding, and scoring differences must drive these results. Our results show that in the STS datasets, the number of informative characters for the UP21 matrix is much higher than in the RE23 matrix, with the latter having around two-thirds of the number of characters in the UP21 STS dataset.

Apart from *Bicharracosaurus*, other putative macronarian taxa are recovered in different position after controlling for taxonomic scope. *Tehuelchesaurus*, *Janenschia*, and *Bellusaurus* recovered as basal macronarians in the RE23 STS analysis, were outside Neosauropoda in the UP21 STS analyses. *Tastavinsaurus* and *Euhelopus* are recovered within basal macronarians in the RE23 STS but inside Somphospondyli in the UP21 STS analysis. *Australodocus* is recovered within Mamenchisauridae in the RE23 STS analysis, but inside Brachiosauridae in the UP21 analysis. *Tendaguria* is still within Titanosauria in the RE23 STS dataset but within Mamenchisauridae in the UP21 STS dataset. Although not tested here, considering the number of characters and the overall phylogenetic incongruences in the STS analyses, there is strong evidence to suggest that character choice has a greater impact on phylogenetic congruence than taxon choice. This is consistent with the study of Rokas & Carroll (2005), whereby the authors showed a positive relationship between gene number and phylogenetic accuracy and a lack of effect of taxon number, in three genome-scale matrices.

Nevertheless, there are potentially additional causes for phylogenetic incongruence. Character coding differences are evident when scoring taxa in both matrices used here (*e.g.*, characters 109 and 14, and characters 134 and 20 of the RE23 and UP21 matrices respectively; see Table S1). Its effect on phylogenetic congruence can hardly be tested and will be addressed elsewhere. Cell scoring differences might also influence the phylogenetic congruence. Differences can occur when scoring multi-species genera (*e.g.*, *Camarasaurus* and *Diplodocus*) or multi-specimen species (*e.g.*, *Jobaria* and *Moabosaurus*). It might also depend on scoring based on personal observations, publications, and/or photographs. A good example for this is *Atlasaurus*, which is included in several analyses (Upchurch, Barrett & Dodson, 2004; Royo-Torres, Cobos & Alcalá, 2006; Wilson & Upchurch, 2009; Royo-Torres & Upchurch, 2012; D’Emic, 2012; Mannion et al., 2013; Mannion et al., 2019b; Poropat et al., 2016; Mannion, Allain & Moine, 2017; Upchurch et al., 2021; Ren et al., 2023) but has rarely been studied in person by researchers (P. Mannion, pers. com., 2024), and only a preliminary description of this important specimen has been published so far (Monbaron, Russell & Taquet, 1999), which is of limited use for coding phylogenetic characters.

Phylogenies are the primary source of information for studies on evolution, including trait evolution, paleobiogeographic distribution patterns, evolutionary rates, among others (Sander et al., 2011; Poropat et al., 2016; Carballido et al., 2017b; Pol et al., 2020; Pol & Ezcurra, 2025). Even though our results show that major lineages within the sauropod tree are conserved (*e.g.*, Mamenchisauridae, Diplodocoidea, and Macronaria), they also suggest that character choice and potentially character state coding and cell scoring have a major influence over the position of certain taxa, especially basal taxa where derived morphologies are not fully developed or present a mosaic pattern of derived and plesiomorphic morphologies. This has the potential to influence our hypotheses on the early evolution of Neosauropoda and Macronaria, and hence their influence on biogeographic scenarios or macroevolutionary conclusions should be evaluated carefully. A thorough comparative cladistics analysis (Sereno, 2009; Goloboff & Sereno, 2021) can help to elucidate these issues and will be addressed elsewhere.

Having several contemporaneous independent morphological matrices is a common thing in vertebrate paleontology. In sauropods, before the matrices of Carballido et al. (2011a) and Mannion et al. (2013), which are the original matrices for the RE23 and UP21 datasets respectively, there were the matrices of Wilson & Sereno (1998) and Upchurch (1998) with subsequent iterations such as Wilson (2002) and Upchurch, Barrett & Dodson (2004). Carballido et al. (2011a) and Mannion et al. (2013), each independently, merged the former matrices with other sources of data, and they and several other work groups have since then been working on these two matrices. In pterosaurs, the classic examples are the matrices of Unwin (2003) and Kellner (2003), or more recently, Andres (2021) Martin-Silverstone et al. (2023) (see Fernandes, Pol & Rauhut (2024). In notosuchians, there are the matrices of Leardi et al. (2015) and Ruiz et al. (2021). We also find this issue in several other groups of dinosaurs (*e.g.*, Butler, Upchurch & Norman, 2008; Cau, Brougham & Naish, 2015; and references therein), highlighting that the issue of matrix composition and its effect on phylogenetic congruence, is not unique to sauropod evolution but is a more general issue in vertebrate paleontology.

## Conclusions

*Bicharracosaurus*, based on an adult partial postcranial skeleton from the Late Jurassic Cañadón Calcáreo Formation, represents an unequivocal macronarian and putative brachiosaurid from the Jurassic of South America. Current matrices available for the study of early evolution of Neosauropoda and especially early-branching macronarian interrelationships, show clear patterns of phylogenetic incongruence influenced mainly by character choice and potentially character coding and character state scoring. For the time being, any hypothesis regarding the origin, early evolution, and early biogeography of Macronaria should be taken carefully particularly concerning the position and influence of certain taxa detected here as sensitive to the choice of dataset or analytical method.

## Supplemental Information

10.7717/peerj.20945/supp-1 Supplemental Information 1 Results of the phylogenetic analyses

10.7717/peerj.20945/supp-2 Supplemental Information 2 Changes included in the phylogenetic analyses

10.7717/peerj.20945/supp-3 Supplemental Information 3 Vertebral laminae terminology and references

10.7717/peerj.20945/supp-4 Supplemental Information 4 RE23 matrix

10.7717/peerj.20945/supp-5 Supplemental Information 5 UP21 matrix

10.7717/peerj.20945/supp-6 Supplemental Information 6 STS RE23 matrix

10.7717/peerj.20945/supp-7 Supplemental Information 7 STS UP21 matrix

10.7717/peerj.20945/supp-8 Supplemental Information 8 Complete list of MorphoSource model DOIs

## Anatomical abbreviations

ACDL: anterior centrodiapophyseal lamina
ACPL: anterior centroparapophyseal lamina
aEI: average Elongation Index
aSPDL: anterior spinodiapophyseal lamina
Ca: caudal vertebrae
Ce: cervical vertebrae
Cer: cervical rib
Ch: chevron
CPOL: centropostzygapophyseal lamina
CPRL: centroprezygapophyseal lamina
Do: dorsal vertebrae
DoR: dorsal rib
lCPOL: lateral centropostzygapophyseal lamina
mCPOL: medial centropostzygapophyseal lamina
mdCPOL: medial division of the centropostzygapophyseal
PACDF: parapophyseal centrodiapophyseal fossa
PCDL: posterior centrodiapophyseal lamina
PCPL: posterior centroparapophyseal lamina
PODL: postzygodiapophyseal lamina
POSDF: postzygapophyseal spinodiapophyseal fossa
POSL: postspinal lamina
PPDL: paradiapophyseal lamina
PRDL: prezygodiapophyseal lamina
PRPL: prezygoparapophyseal lamina
PRSL: prespinal lamina
pSPDL: posterior spinodiapophyseal lamina
Sa: sacral vertebrae
SPDL: spinodiapophyseal lamina
SPOL: spinopostzygapophyseal lamina
SPRL: spinoprezygapophyseal lamina
sTPOL: single interpostzygapophyseal lamina
sTPRL: single interprezygapophyseal lamina
TPOL: interpostzygapophyseal lamina
TPRL: interprezygapophyseal lamina

## Institutional abbreviations

BYU: Brigham Young University, Museum of Paleontology, Provo, Utah, USA
CM: Carnegie Museum of Natural History, Pittsburgh, Pennsylvania, USA
CMNH: Cleveland Museum of Natural History, Cleveland, Ohio, USA
DFMMh: Dinosaurier-Freilichtmuseum Münchehagen, Germany
FMNH: Field Museum of Natural History, Chicago, Illinois, USA
GCP: Grupo Cultural Paleontológico de Elche, Elche, Spain
MB.R.: Museum für Naturkunde Berlin, Collection of Fossil Reptiles, Berlin, Germany
MNN: Musée National du Niger, Niamey, Republic of Niger
MPEF: Museo Paleontológico Egidio Feruglio, Trelew, Argentina
PMU: Paleontological Museum Uppsala, Uppsala, Sweden
PVL: Instituto Miguel Lillo, Colección Paleontología de Vertebrados, Tucumán, Argentina
SMA: Sauriermuseum Aathal, Aathal, Switzerland
UMNH: Natural History Museum of Utah, Salt Lake City, Utah, USA

## Additional Information and Declarations

### Data Availability

The following information was supplied regarding data availability: The results of the phylogenetic analyses, vertebral laminae terminology and references, and matrices are available in the Supplemental Files. The 26 3D models are available at MorphoSource; the complete list is available in the Supplemental Files.

### New Species Registration

The following information was supplied regarding the registration of a newly described species: Publication LSID: urn:lsid:zoobank.org:pub:A6452246-4BB8-471A-A943-6EA2DD964360 Genus *Bicharracosaurus* LSID: urn:lsid:zoobank.org:act:3949E86C-953B-43BC-A46F-7CC0C6C9B2D3, Species *B. dionidei* LSID: urn:lsid:zoobank.org:act:71DC4A62-78B6-456B-B528-33528A4D6673,
