# Supplementary material for: Bicharracosaurus dionidei, gen. et sp. nov., a new macronarian (Dinosauria, Sauropoda) from the Late Jurassic Cañadón Calcáreo Formation of Argentina and the problematic early evolution of macronarians
Source: PeerJ. 2026 Apr 16;14:e20945. doi: 10.7717/peerj.20945 (PMC13092234; doi:10.7717/peerj.20945)
Supplement: Supplemental Information 1 [file peerj-14-20945-s001.docx]

**Supplemental Article for:**

***Bicharracosaurus dionidei*, gen. et sp. nov., a new brachiosaurid (Sauropoda, Macronaria) from the Late Jurassic Cañadón Calcáreo Formation of Argentina and the problematic early evolution of macronarians**

Alexandra Reutter^1^, José L. Carballido^2,3^, Guillermo J. Windholz^3,4^, Diego Pol^3,5^, Oliver W. M. Rauhut^1,6,7^

^1^ Department of Earth and Environmental Sciences, Ludwig-Maximilians-Universität München, Munich, Germany

^2^ Museo Paleontológico Egidio Feruglio, Trelew, Argentina

^3^ Consejo Nacional de Investigaciones Científicas y Técnicas, Buenos Aires, Argentina

^4^ Instituto de Investigación en Paleobiología y Geología, Universidad Nacional de Río Negro, General Roca, Argentina

^5^ Museo Argentino de Ciencias Naturales Bernardino Rivadavia, Buenos Aires, Argentina

^6^ Bayerische Staatssammlung für Paläontologie und Geologie, Staatliche Naturwissenschaftliche Sammlungen Bayerns, Munich, Germany

^7^ GeoBioCenter^LMU^, Ludwig-Maximilians-Universität München, Munich, Germany

**
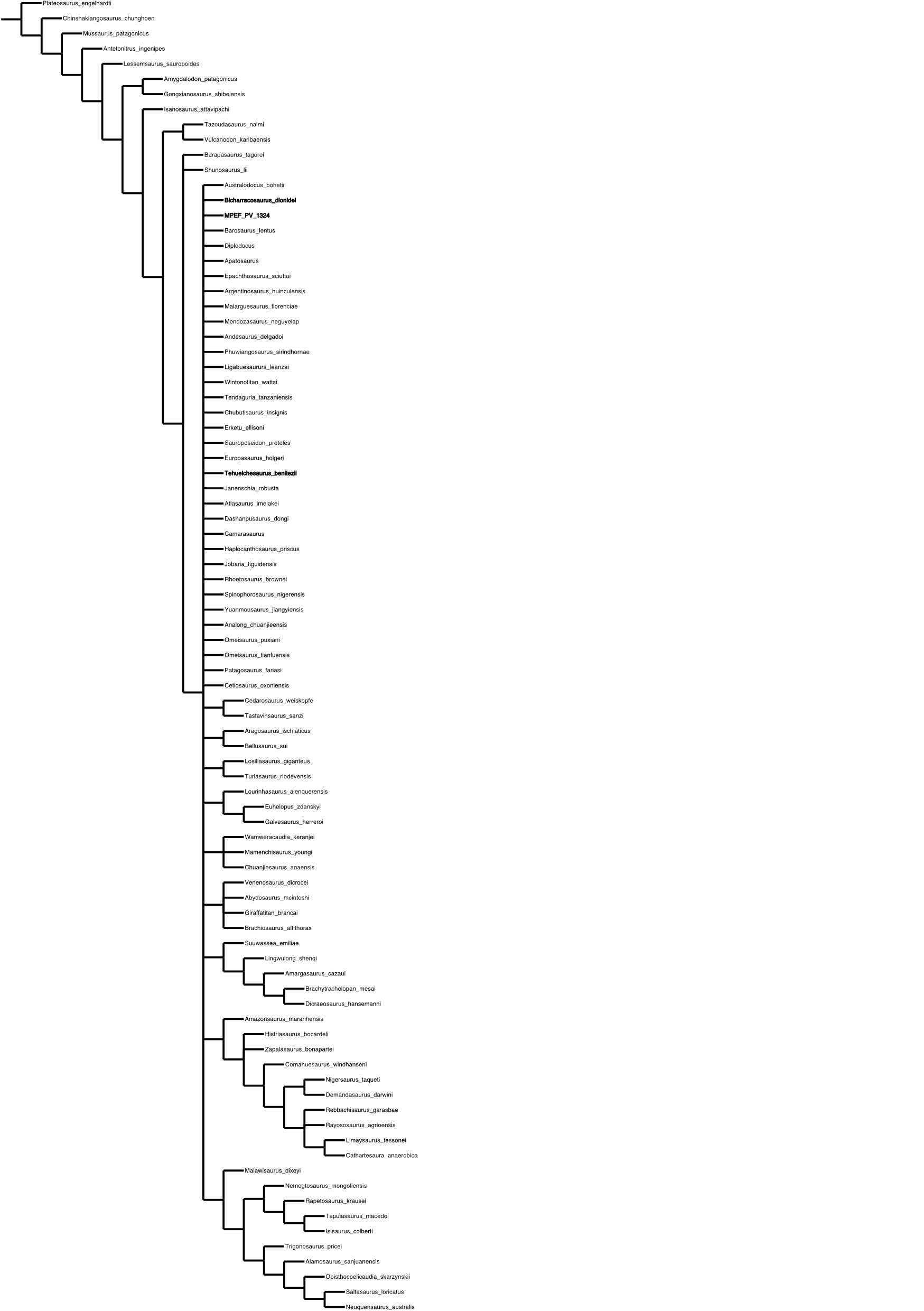
**

**Figure 1. Strict consensus tree of the equal weights analysis using the Ren et al. (2023) matrix.**

**
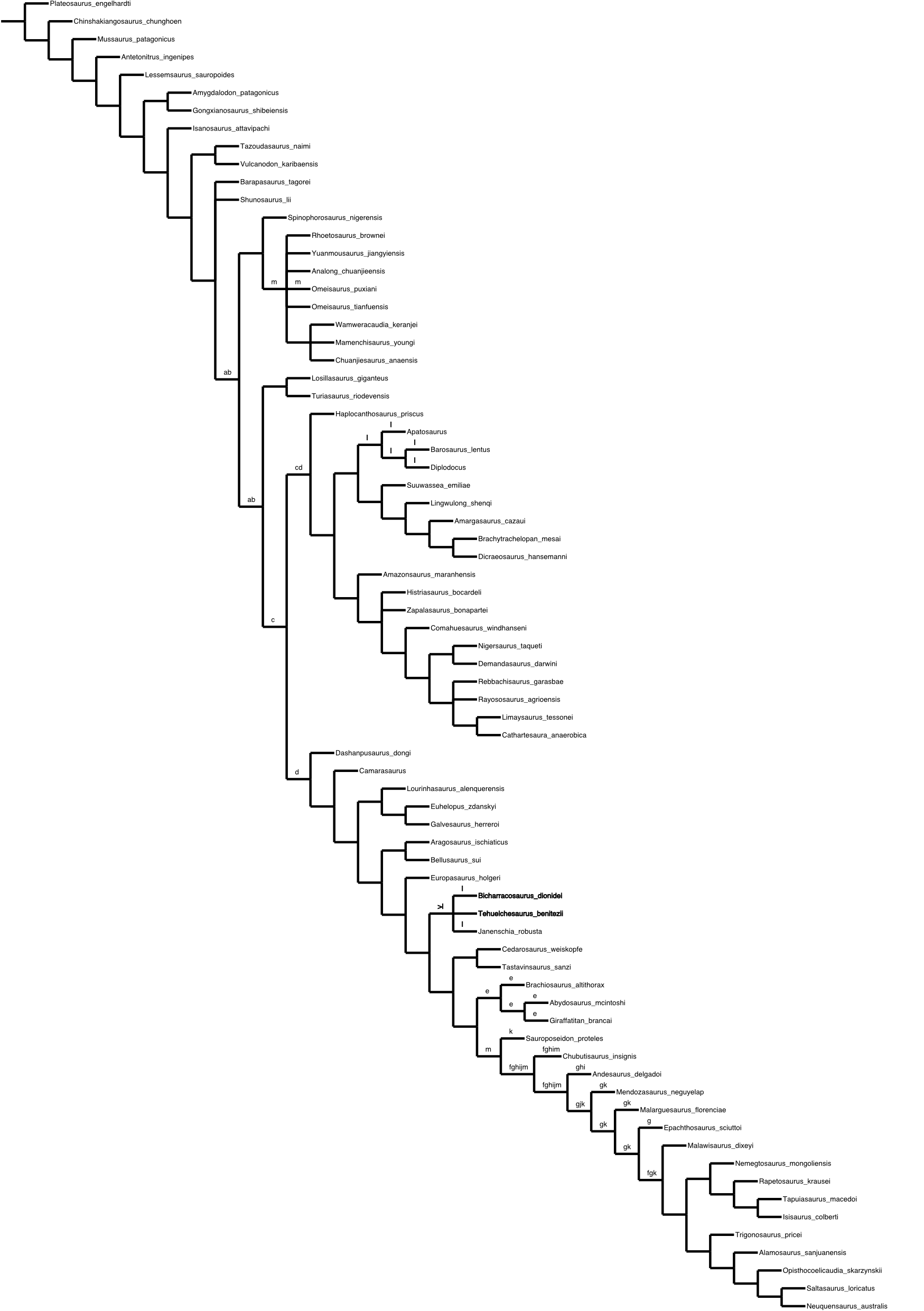
**

**Figure 2. Reduced consensus tree of the equal weights analysis using the Ren et al. (2023) matrix.**

*A posteriori* pruning of (a) *Cetiosaurus oxoniensis*; (b) *Patagosaurus fariasi*; (c) *Jobaria tiguidensis*; (d) *Atlasaurus imelakei*; (e) *Venenosaurus dicrocei*; (f) *Erketu ellisoni*; (g) *Tendaguria tanzaniensis*; (h) *Wintonotitan wattsi*; (i) *Ligabuesaurus leanzai*; (j) *Phuwiangosaurus sirindhornae*; (k) *Argentinosaurus huinculensis*; (l) MPEF PV 1324 and (m) *Australodocus bohetii*.

**
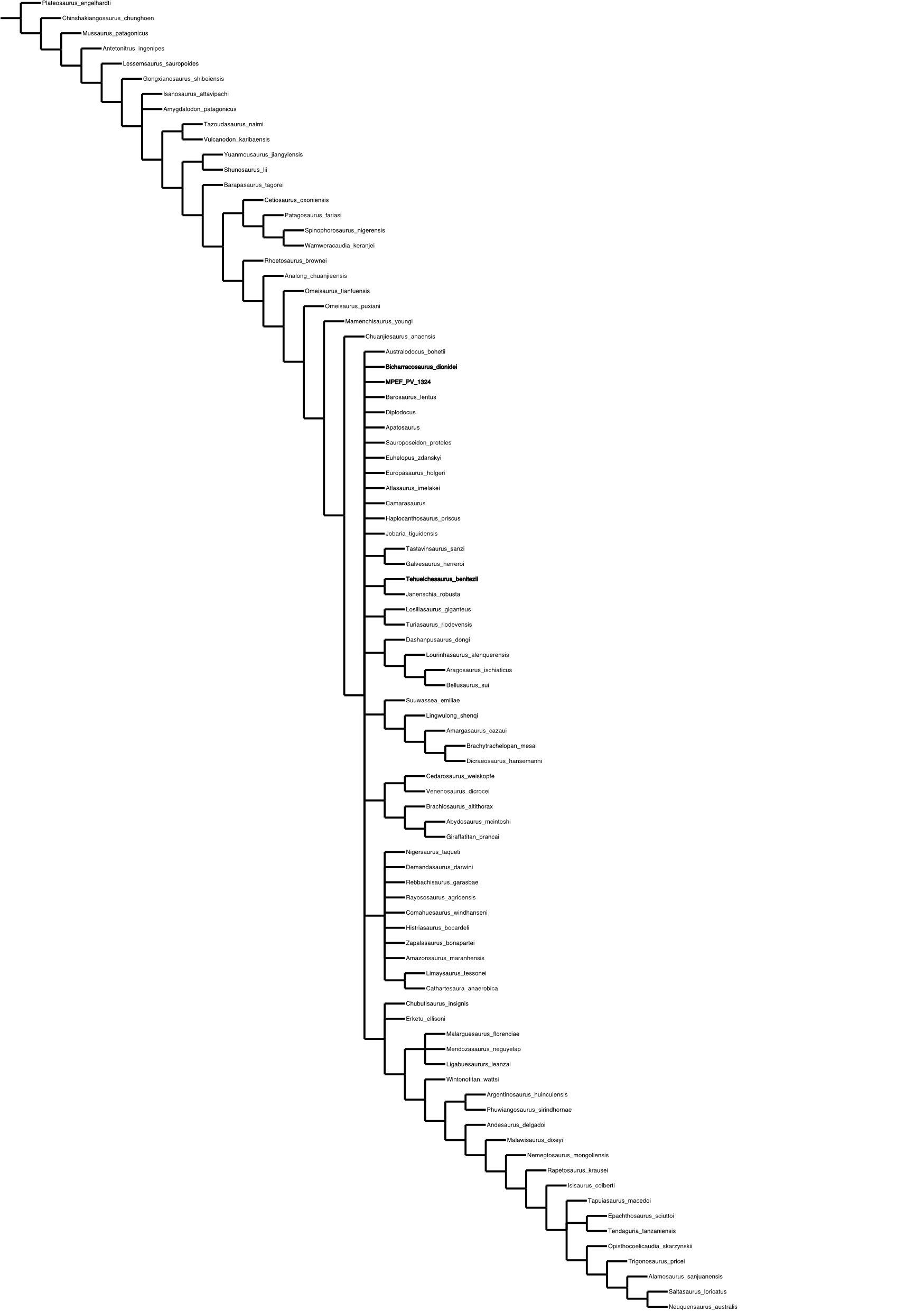
**

**Figure 3. Strict consensus tree of the extended implied weights (k = 3) analysis using the Ren et al. (2023) matrix.**

**
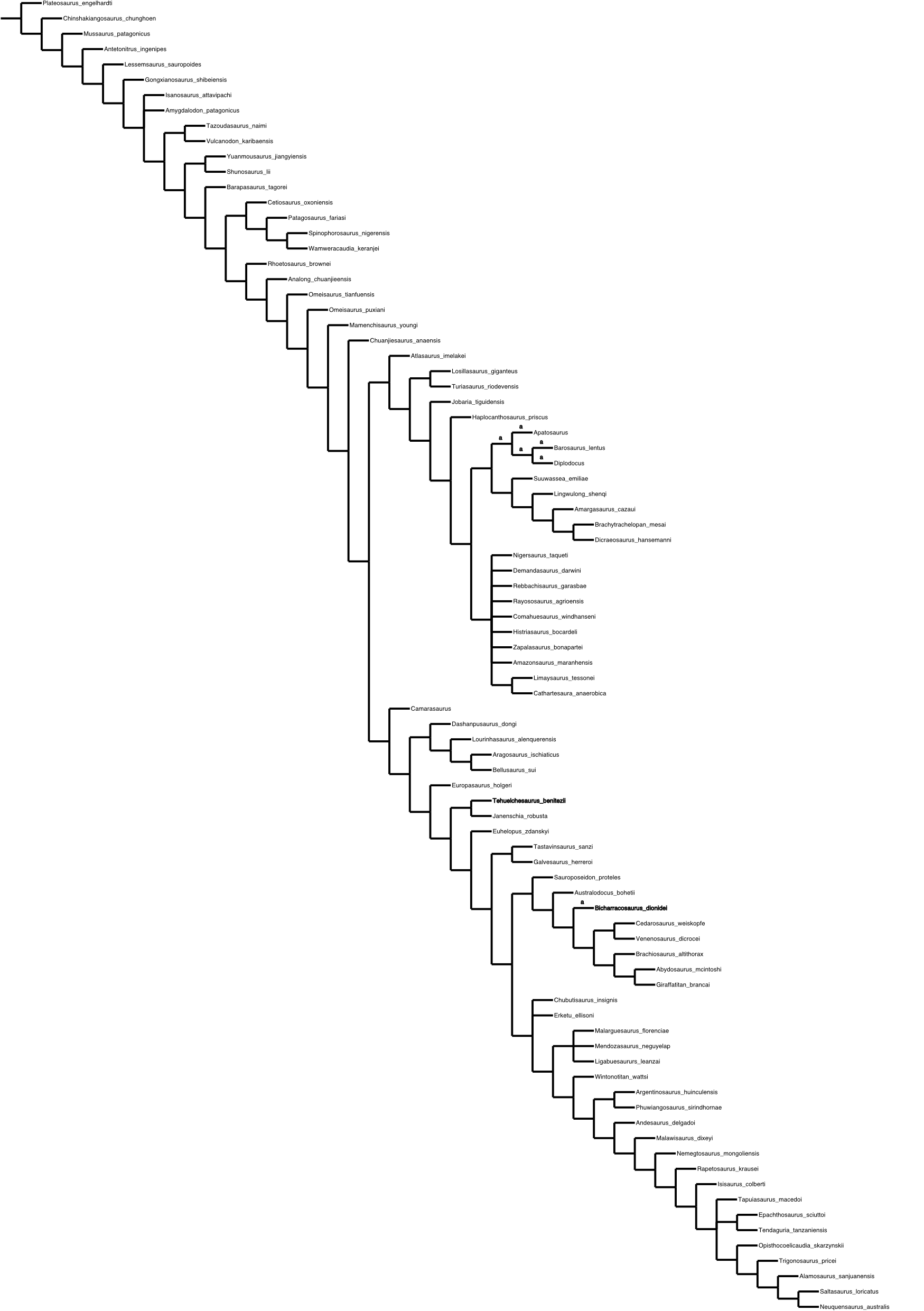
Figure 4. Reduced consensus tree of the extended implied weights (k = 3) analysis using the Ren et al. (2023) matrix.**

*A posteriori* pruning of (a) MPEF-PV 1324.

**
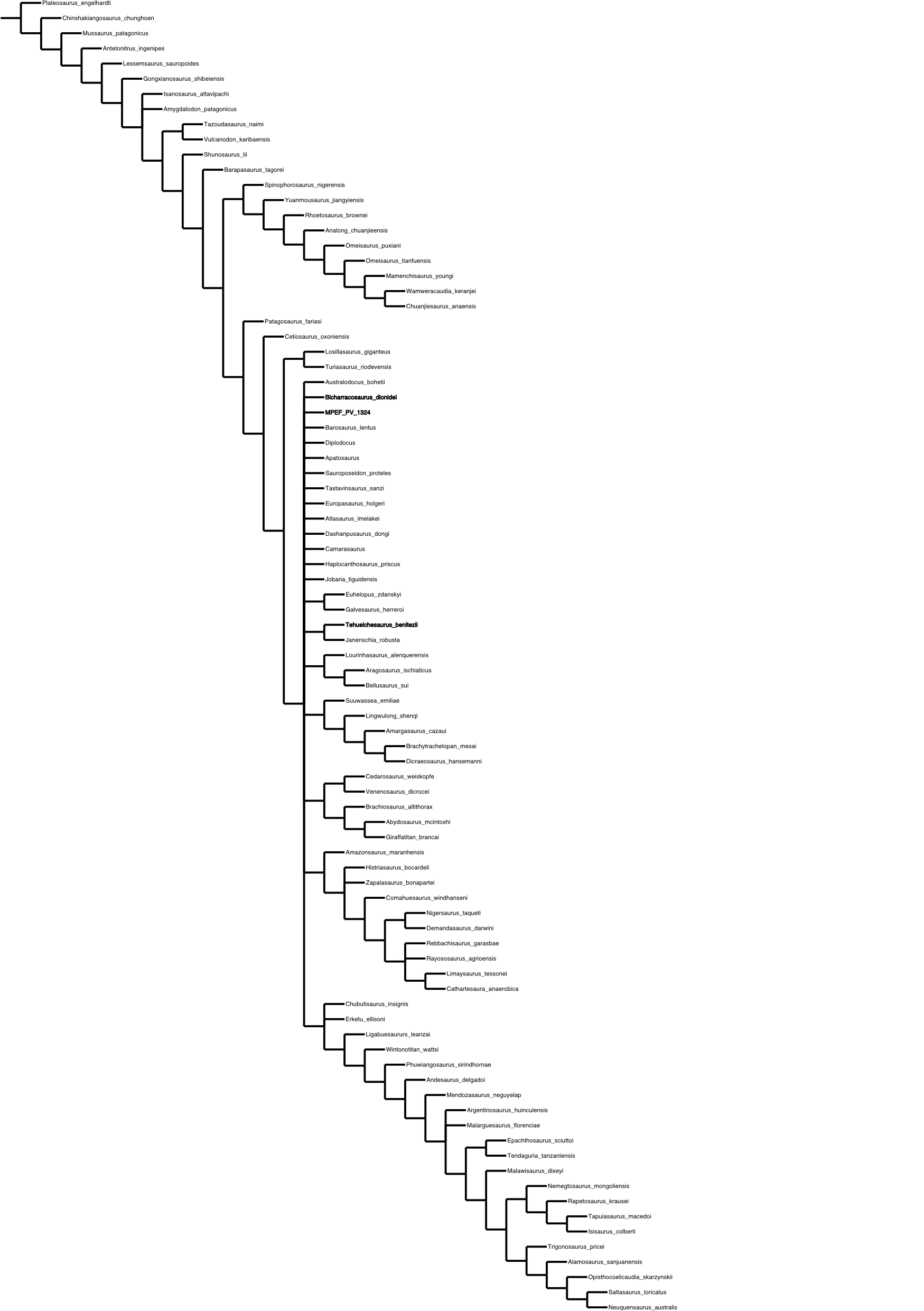
**

**Figure 5. Strict consensus tree of the extended implied weights (k = 8) analysis using the Ren et al. (2023) matrix.**

**
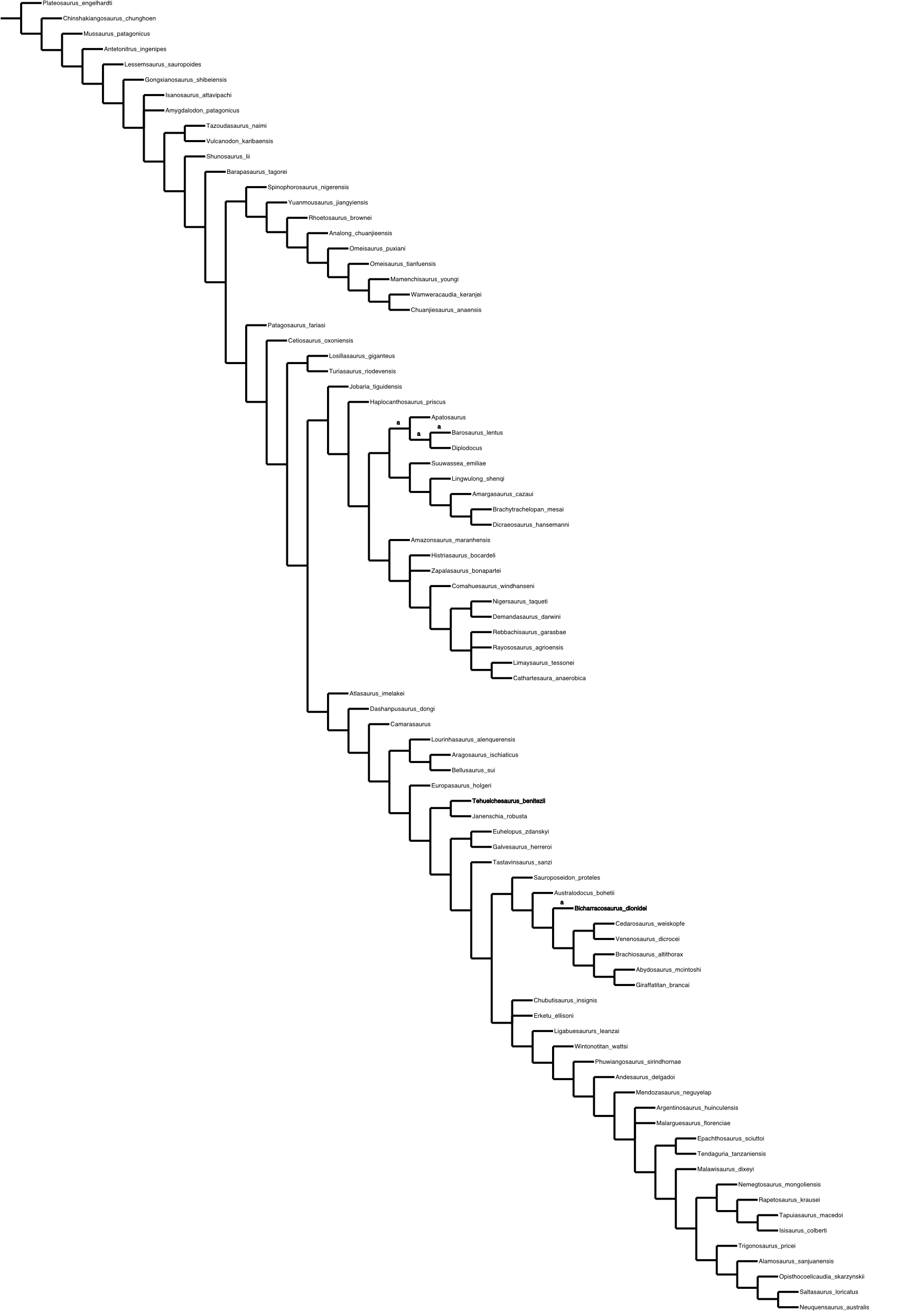
**

**Figure 6. Reduced consensus tree of the extended implied weights (k = 8) analysis using the Ren et al. (2023) matrix.**

*A posteriori* pruning of (a) MPEF-PV 1324.

**
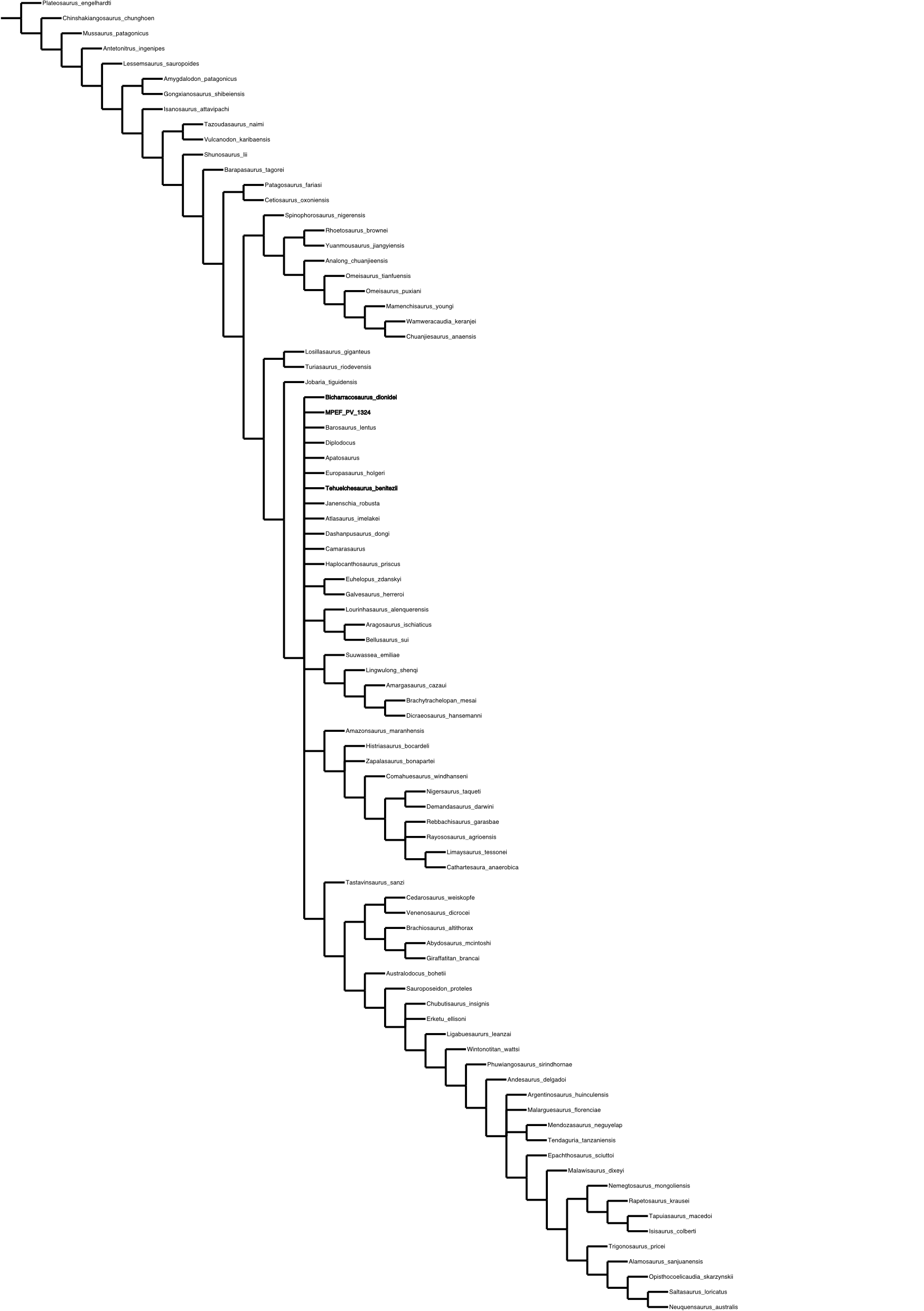
Figure 7. Strict consensus tree of the extended implied weights (k = 13) analysis using the Ren et al. (2023) matrix.**

**
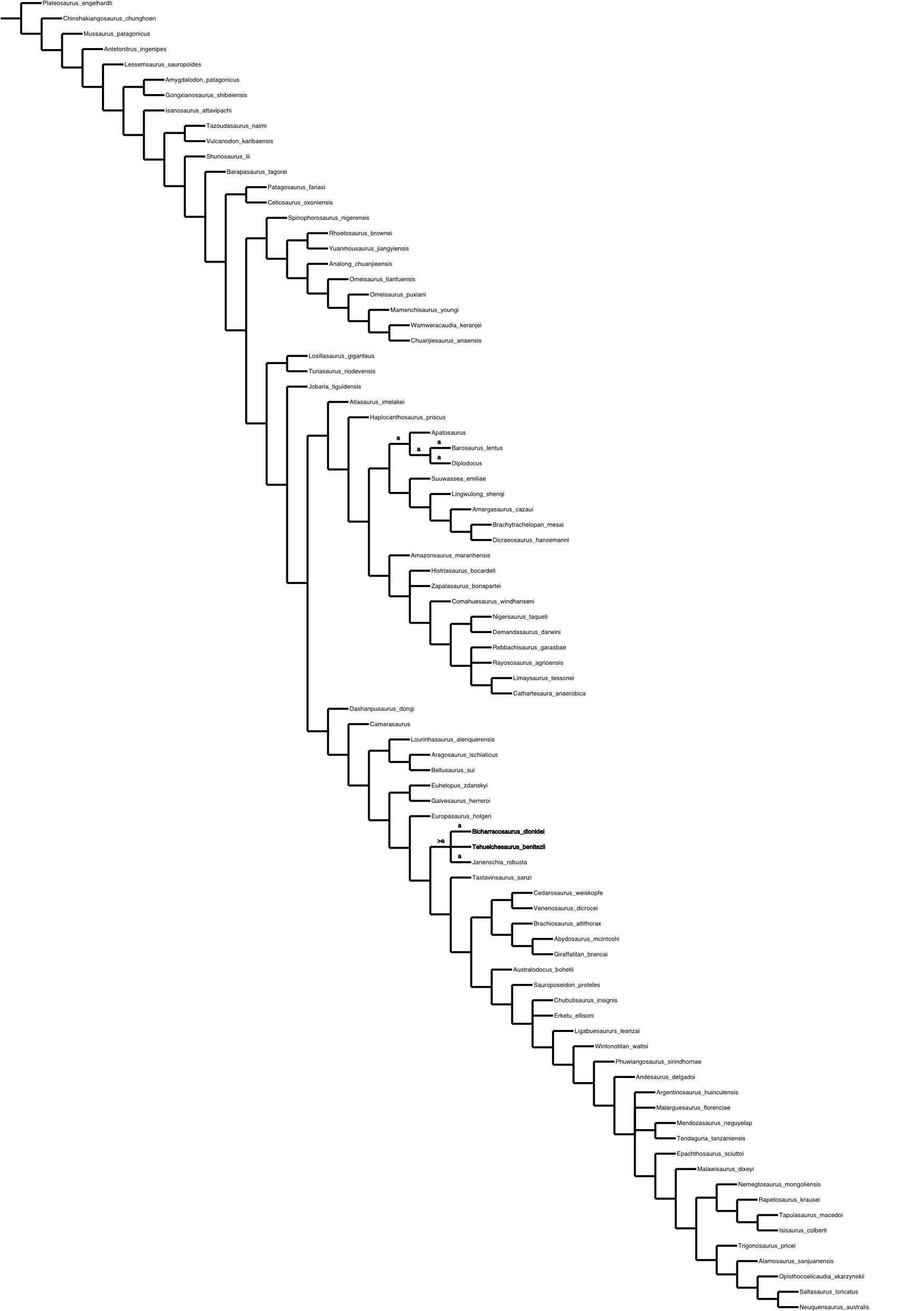
**

**Figure 8. Reduced consensus tree of the extended implied weights (k = 13) analysis using the Ren et al. (2023) matrix.**

*A posteriori* pruning of (a) MPEF-PV 1324.

**
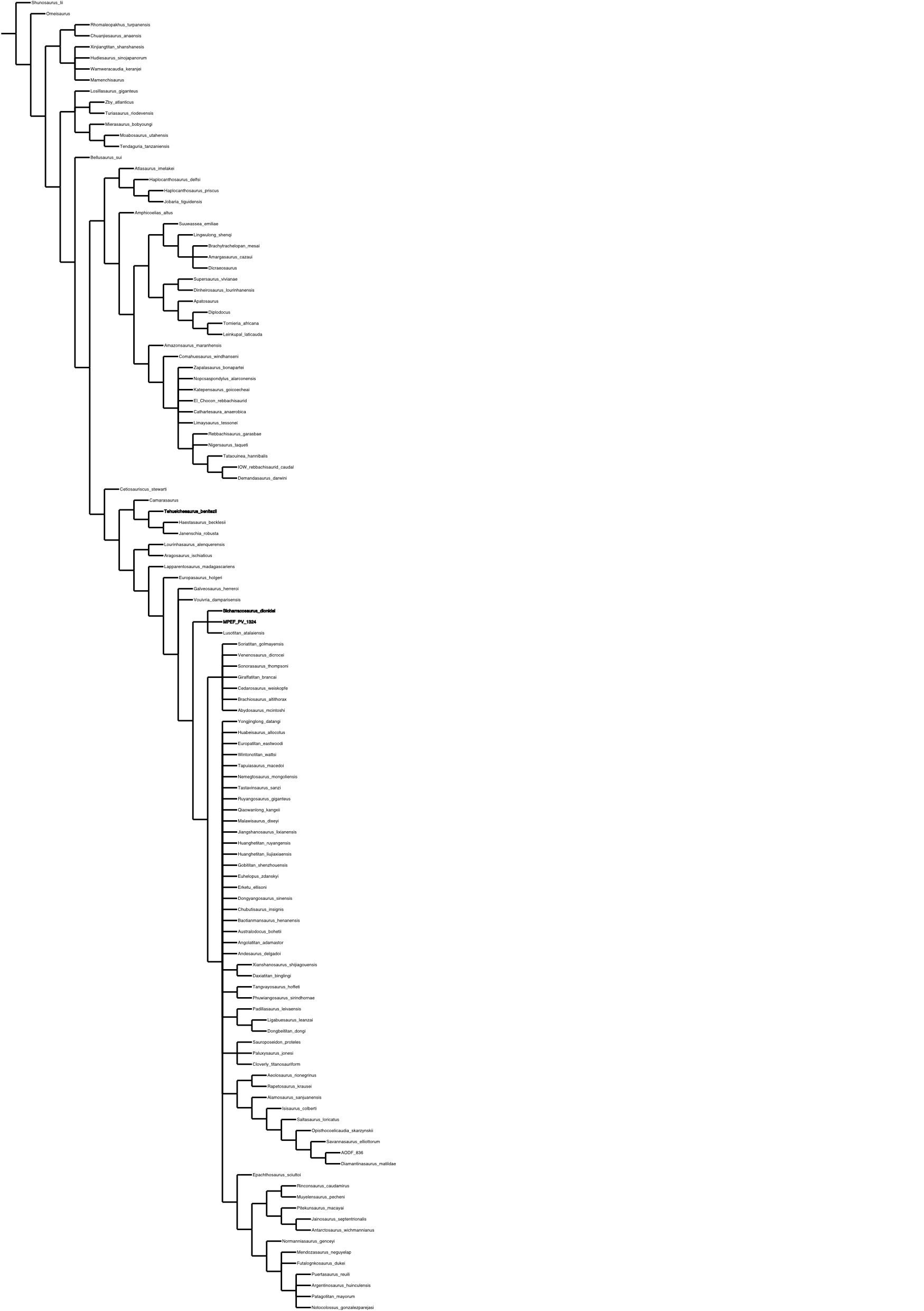
**

**Figure 9. Strict consensus tree of the equal weights analysis using the Upchurch et al. (2021) matrix.**


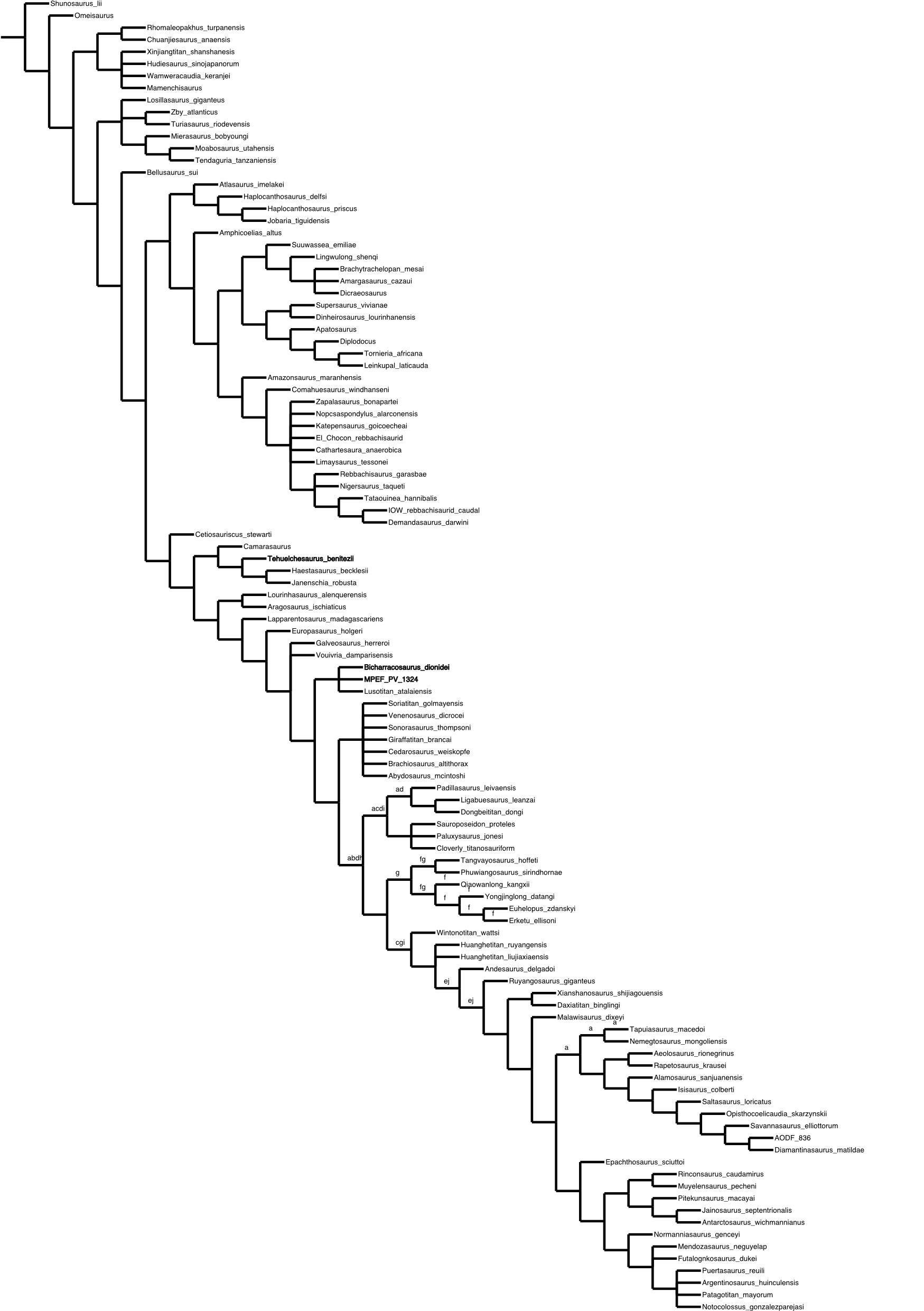


**Figure 10. Reduced consensus tree of the equal weights analysis using the Upchurch et al. (2021) matrix.**

*A posteriori* pruning of (a) *Angolatitan adamastor*, (b) *Australodocus bohetii*, (c) *Baotianmansaurus henanensis*, (d) *Chubutisaurus insignis*, (e) *Dongyangosaurus sinensis*, (f) *Gobititan shenzhouensis*, (g) *Jiangshanosaurus lixianensis*, (h) *Tastavinsaurus sanzi*, (i) *Europatitan eastwoodi* and (j) *Huabeisaurus allocotus*.


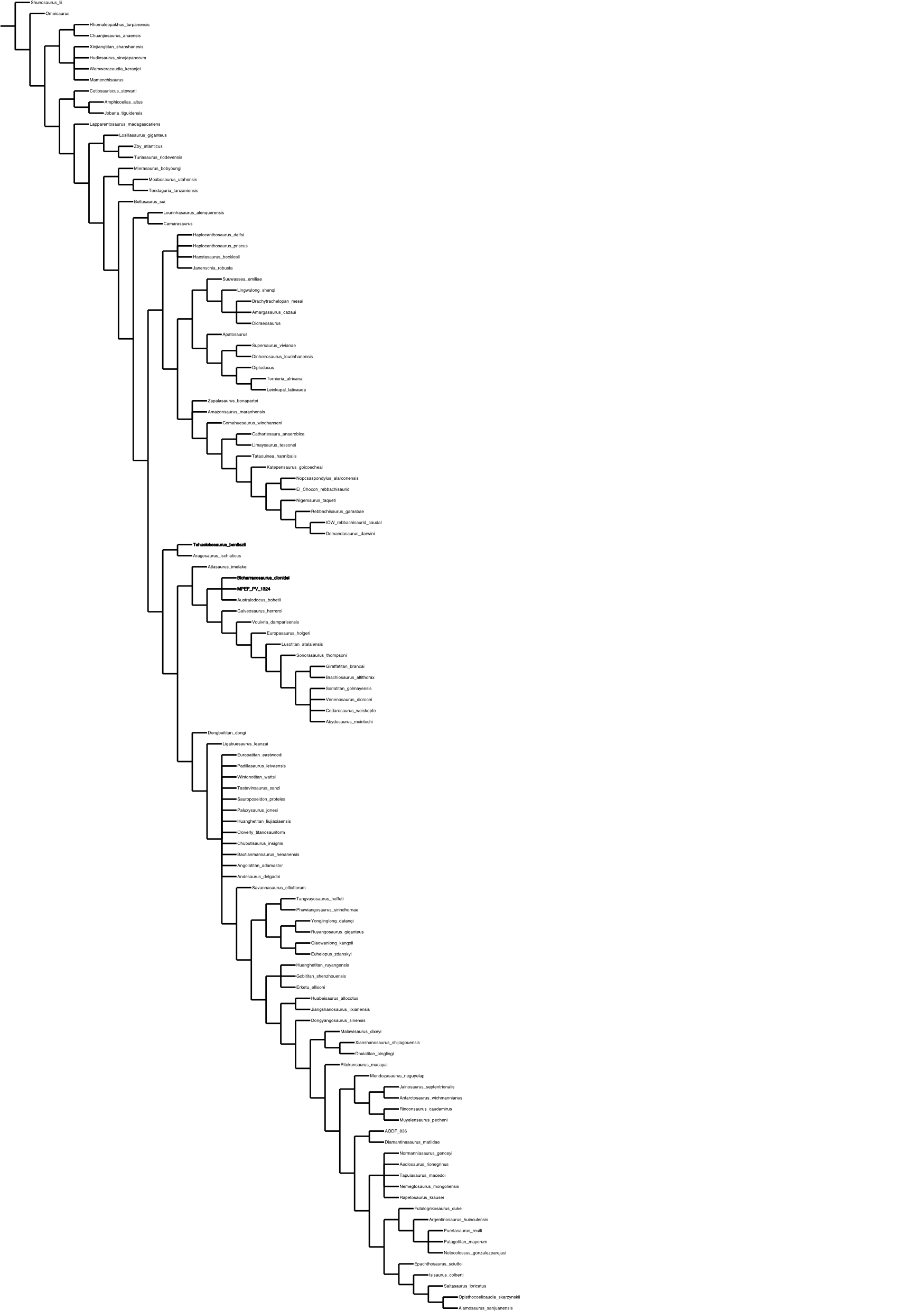


**Figure 11. Strict consensus tree of the extended implied weights (k = 3) analysis using the Upchurch et al. (2021) matrix.**

**
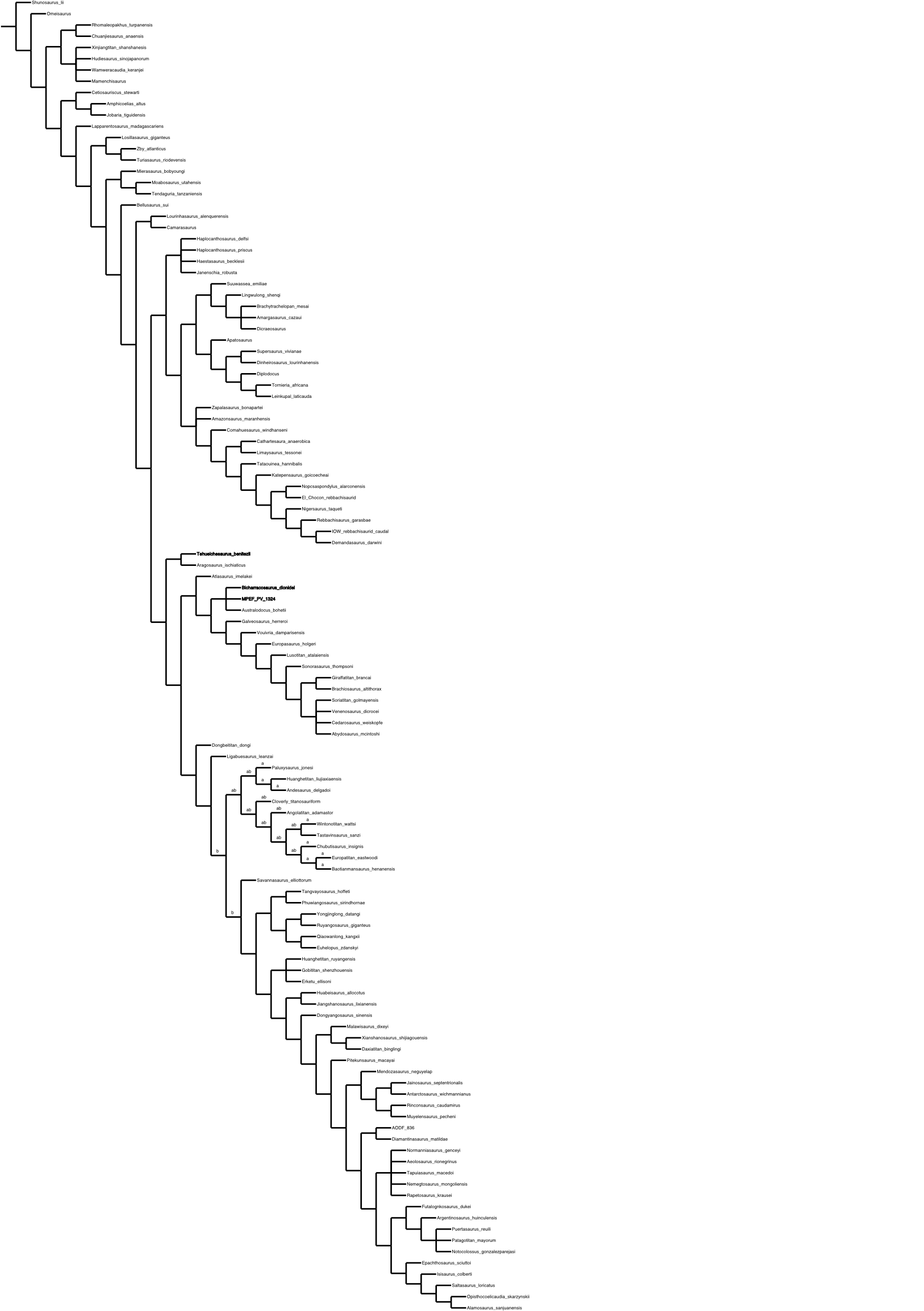
**

**Figure 12. Reduced consensus tree of the extended implied weights (k = 3) analysis using the Upchurch et al. (2021) matrix.**

*A posteriori* pruning of (a) *Sauroposeidon proteles* and (b) *Padillasaurus leivaensis*.

**
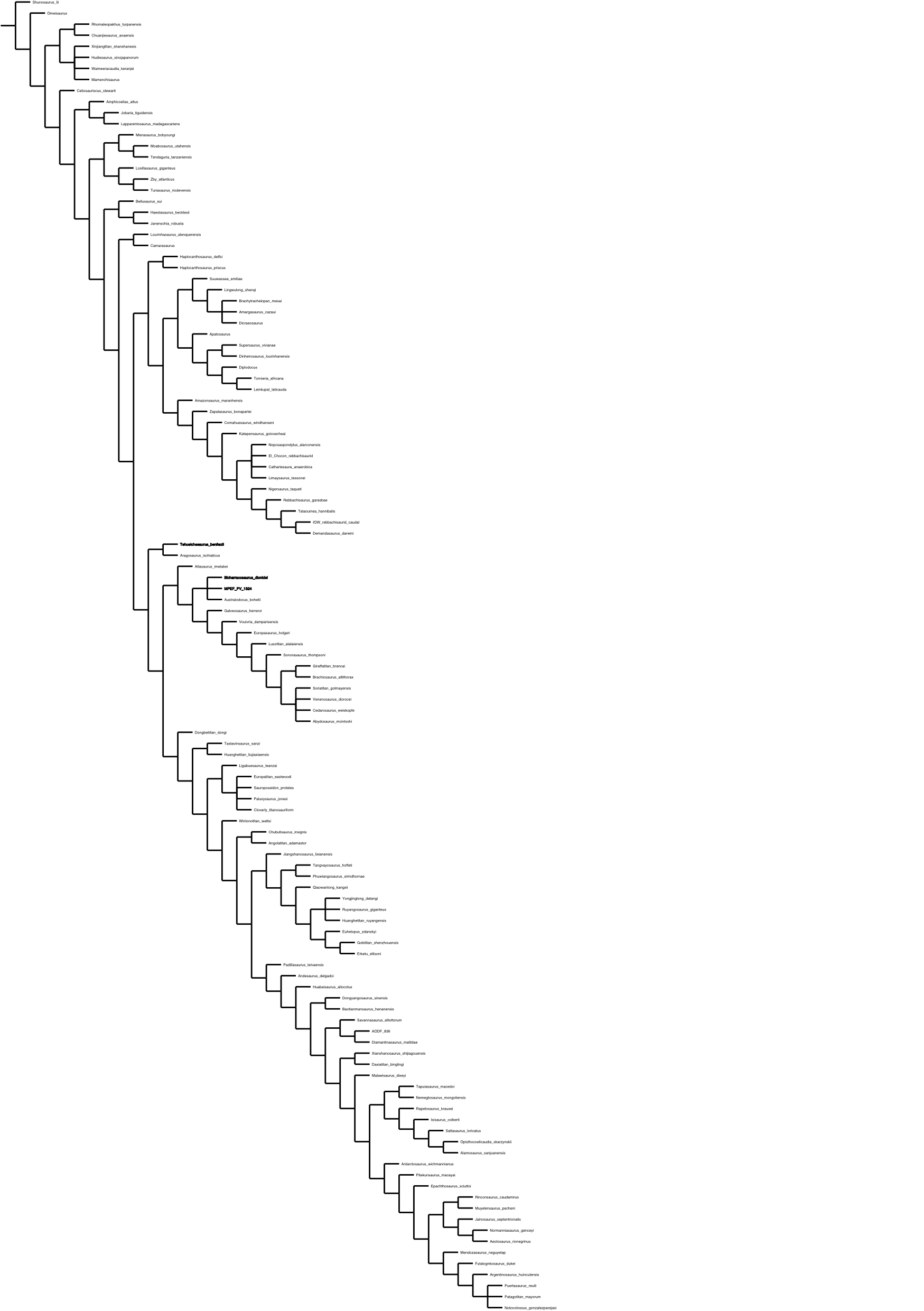
**

**Figure 13. Strict consensus tree of the extended implied weights (k = 8) analysis using the Upchurch et al. (2021) matrix.**

**
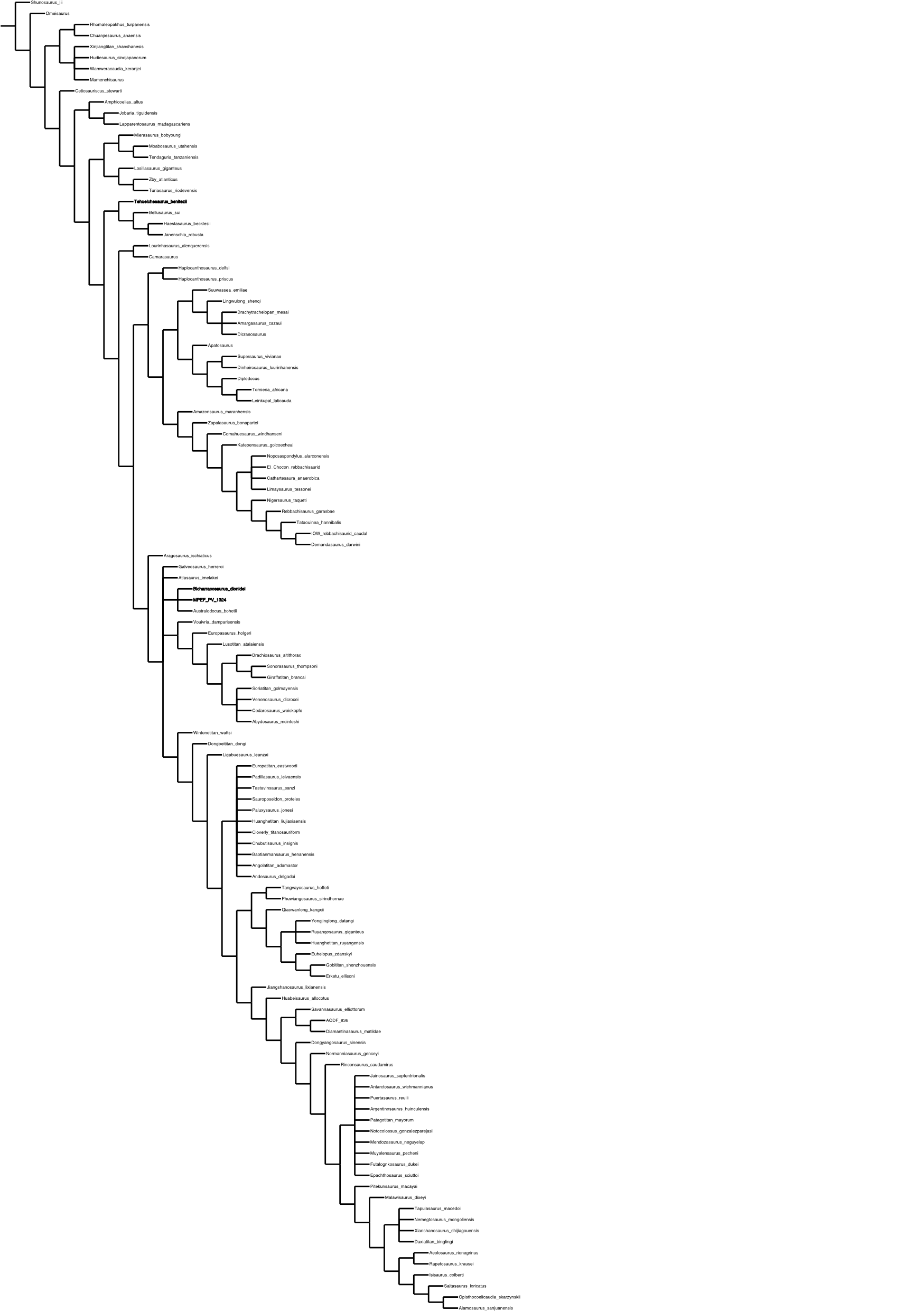
**

**Figure 14. Strict consensus tree of the extended implied weights (k = 13) analysis using the Upchurch et al. (2021) matrix.**

**
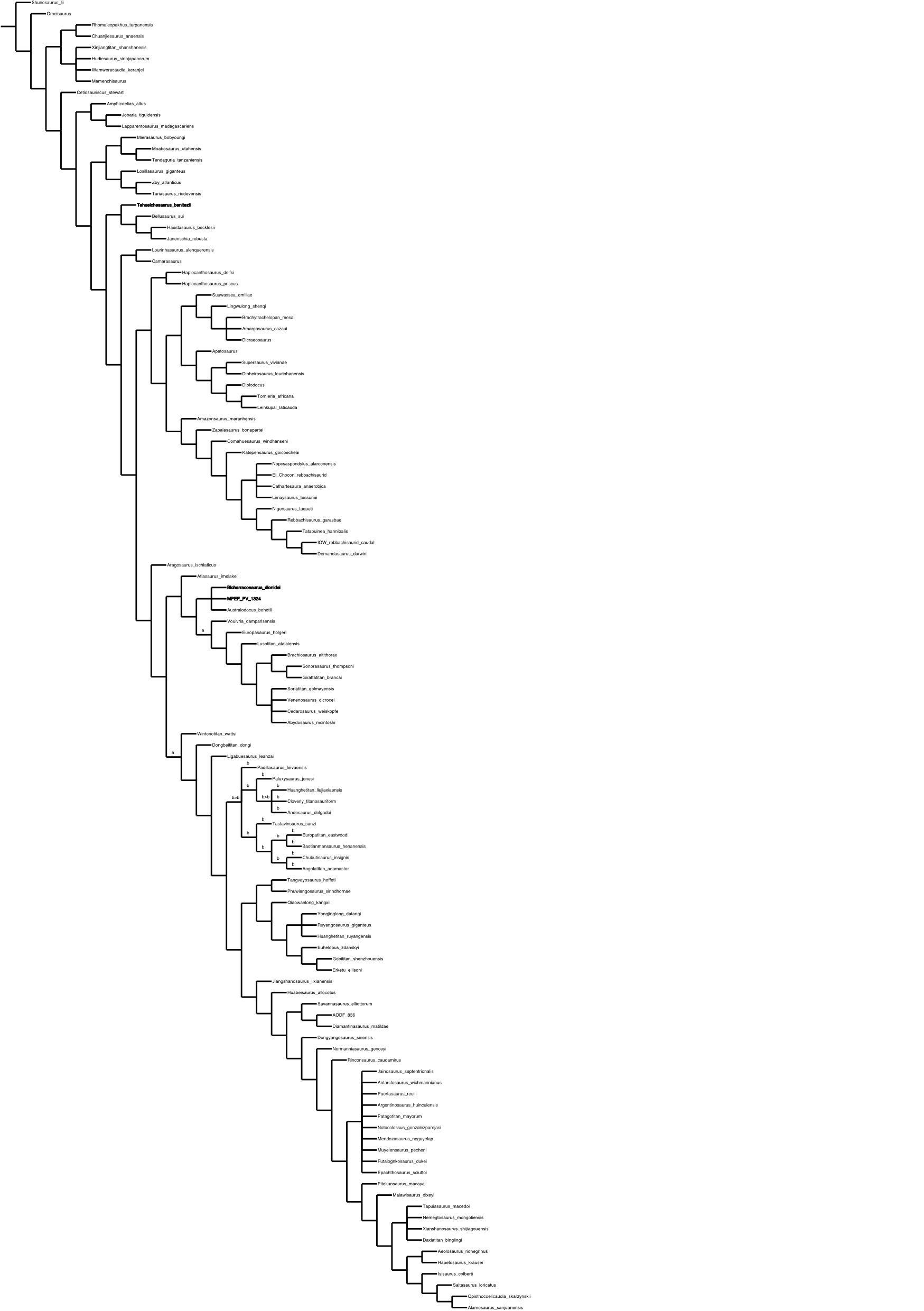
**

**Figure 15. Reduced consensus tree of the extended implied weights (k = 13) analysis using the Upchurch et al. (2021) matrix.**

*A posteriori* pruning of (a) *Galvesaurus herreroi* and (b) *Sauroposeidon proteles*.

**
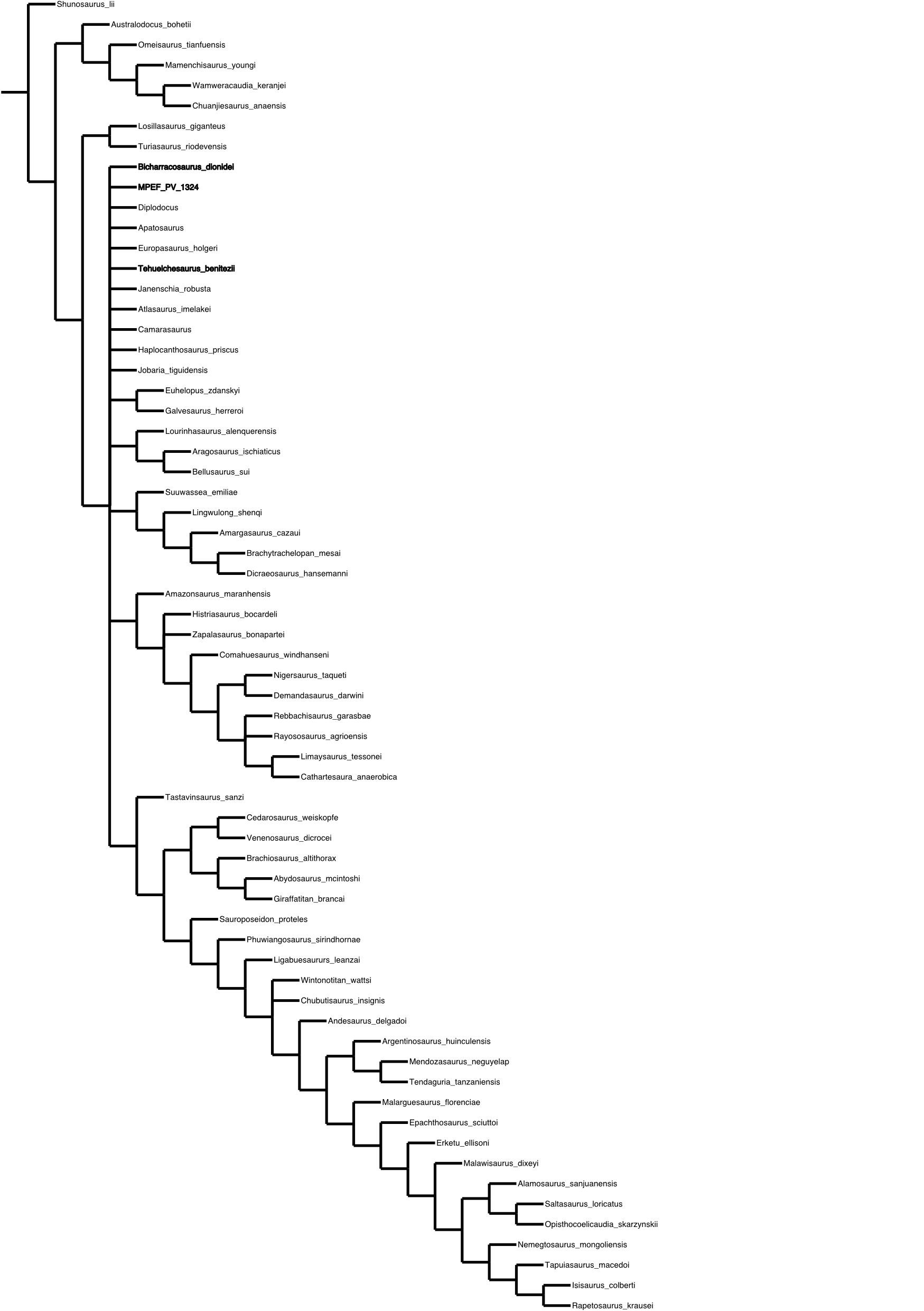
**

**Figure 16. Strict consensus tree of the shared taxonomic scope analysis using the Ren et al. (2023) matrix.**

**
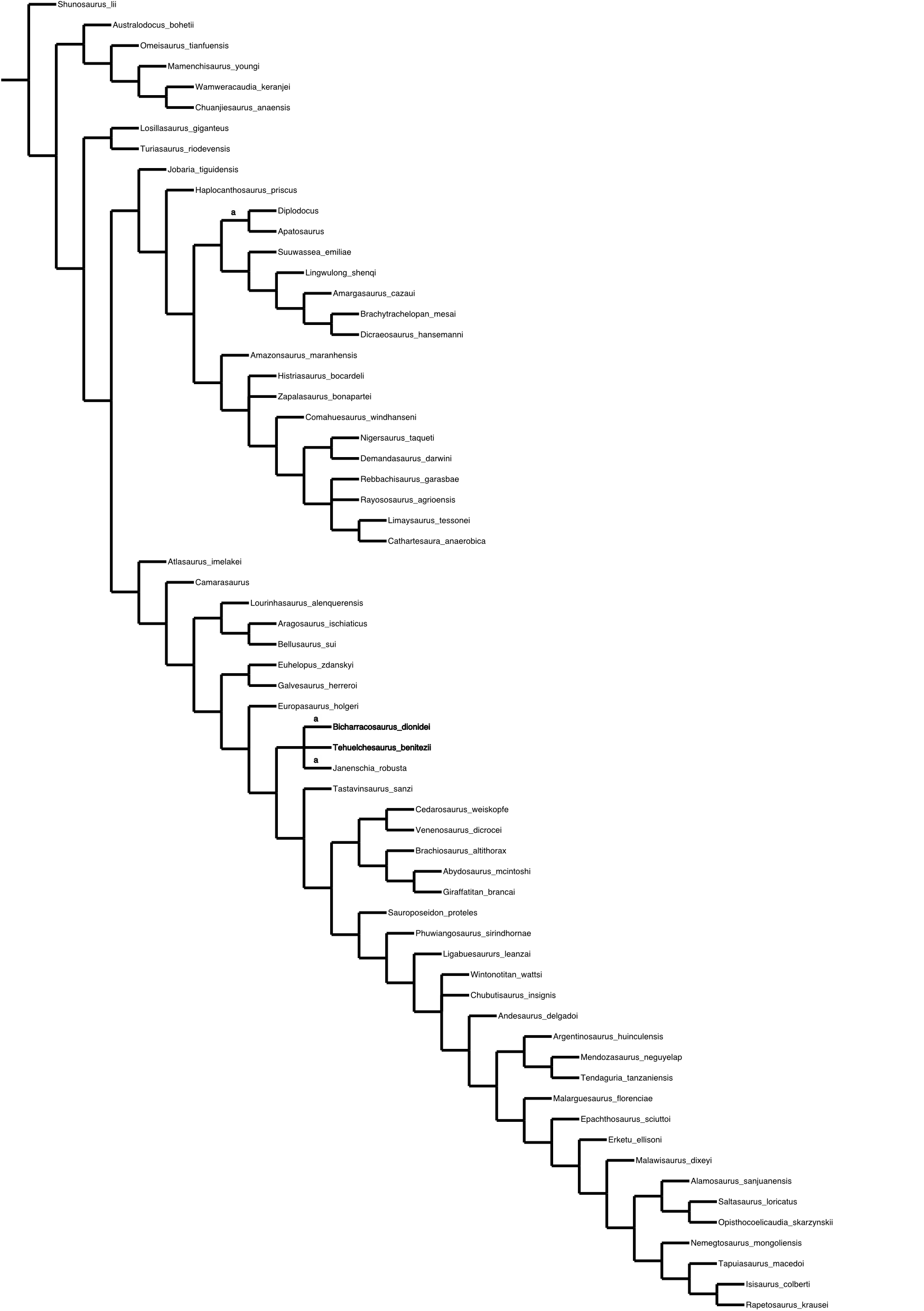
**

**Figure 17. Reduced consensus tree of the shared taxonomic scope analysis using the Ren et al. (2023) matrix.**

*A posteriori* pruning of (a) MPEF-PV 1324.

**
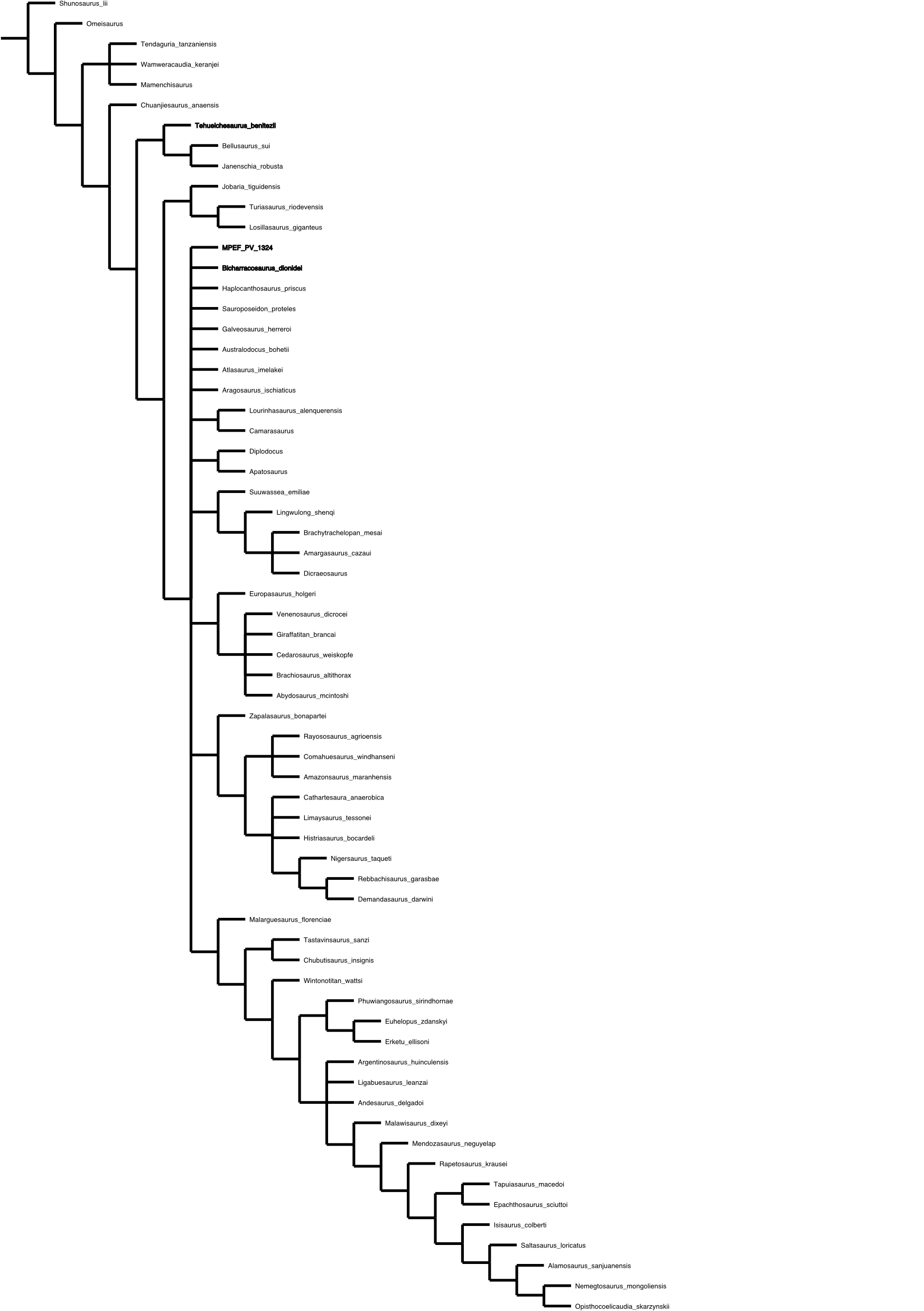
**

**Figure 18. Strict consensus tree of the shared taxonomic scope analysis using the Upchurch et al. (2021) matrix.**

**
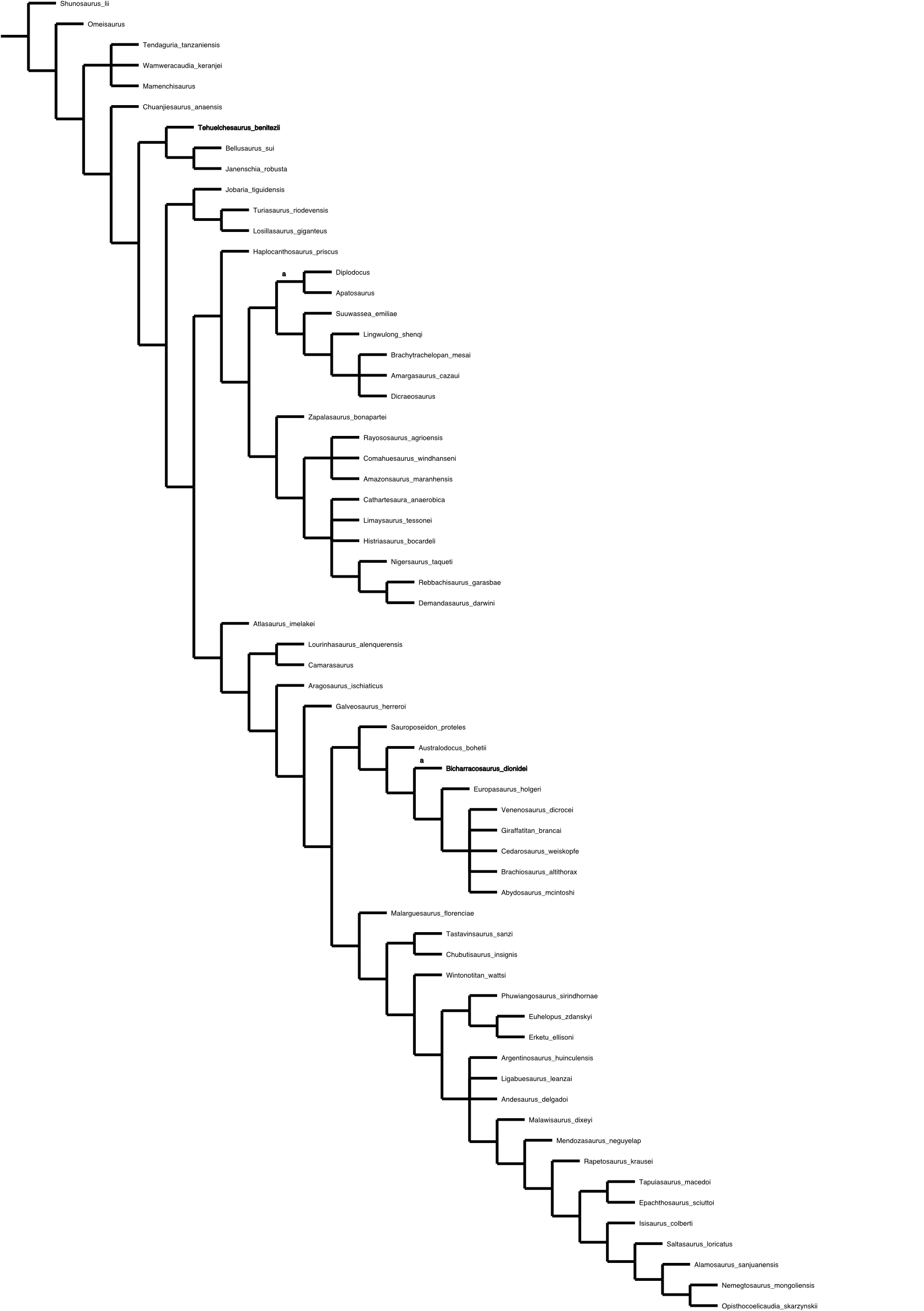
**

**Figure 19. Reduced consensus tree of the shared taxonomic scope analysis using the Upchurch et al. (2021) matrix.**

*A posteriori* pruning of (a) MPEF-PV 1324.
